# An eDNA Assay to Monitor a Globally Invasive Fish Species from Flowing Freshwater

**DOI:** 10.1371/journal.pone.0147558

**Published:** 2016-01-27

**Authors:** Irene Adrian-Kalchhauser, Patricia Burkhardt-Holm

**Affiliations:** 1 University of Basel, Department of Environmental Sciences, Basel, Switzerland; 2 Department of Biological Sciences, University of Alberta, Edmonton, Canada; Chinese Academy of Fishery Sciences, CHINA

## Abstract

Ponto-Caspian gobies are a flock of five invasive fish species that have colonized freshwaters and brackish waters in Europe and North America. One of them, the round goby *Neogobius melanostomus*, figures among the 100 worst invaders in Europe. Current methods to detect the presence of Ponto-Caspian gobies involve catching or sighting the fish. These approaches are labor intense and not very sensitive. Consequently, populations are usually detected only when they have reached high densities and when management or containment efforts are futile. To improve monitoring, we developed an assay based on the detection of DNA traces (environmental DNA, or eDNA) of Ponto-Caspian gobies in river water. The assay specifically detects invasive goby DNA and does not react to any native fish species. We apply the assay to environmental samples and demonstrate that parameters such as sampling depth, sampling location, extraction protocol, PCR protocol and PCR inhibition greatly impact detection. We further successfully outline the invasion front of Ponto-Caspian gobies in a large river, the High Rhine in Switzerland, and thus demonstrate the applicability of the assay to lotic environments. The eDNA assay requires less time, equipment, manpower, skills, and financial resources than the conventional monitoring methods such as electrofishing, angling or diving. Samples can be taken by untrained individuals, and the assay can be performed by any molecular biologist on a conventional PCR machine. Therefore, this assay enables environment managers to map invaded areas independently of fishermen’s’ reports and fish community monitorings.

## Introduction

Invasive Ponto-Caspian gobies are a flock of five invasive fish species (*Neogobius melanostomus*, *Ponticola kessleri*, *Neogobius fluviatilis*, *Proterorhinus semilunaris*, and *Babka gymnotrachelus*) that are native to the Black and Caspian Seas and their tributaries (hence the name of the group). Members of this group have colonized freshwaters and brackish waters in Europe and North America and are expected to expand further [1, 2]. All five species are small, benthic, and predatory fish. They impact native ecosystems in a variety of ways as predator, prey, and competitor [3]. Their natural home range is small [4]. Their rapid range expansion has therefore been attributed to the vector activities of ships [5, 6].

Current methods used for monitoring small, benthic fish such as invasive Ponto-Caspian gobies in major waterways are labor intense, require expensive equipment or skills and are not very sensitive. For example, fish community monitorings are often carried out by wading river banks. Ponto-Caspian gobies, however, first appear at commercial infrastructures such as harbors [6, 7]. Also, Ponto-Caspian gobies are hard to catch with the electrofishing gear usually employed in monitorings, since they hide under stones at several meters depth and lack a swim bladder [8]. The arrival of Ponto-Caspian gobies in a water body is thus usually detected by targeted approaches using alternative catch methods at putative introduction sites [7], or by attentive anglers, when the population has spread from the introduction sites into fishing grounds.

Environmental DNA (eDNA) assays detect the presence of a species from traces the organism leaves behind in the environment, without the need to actually catch the organism. eDNA is particularly well suited for species which are hard to detect with conventional sampling schemes and techniques. To detect aquatic species, water samples are collected, DNA is extracted from the water, and the presence of a species is determined with species-specific PCR assays or with community sequencing approaches [9]. eDNA samplings typically require less effort in terms of person hours and financial resources than conventional monitoring approaches [10], they cause less environmental disturbance [11], and can even be carried out at the community level by citizens [12]. The detectability of a species by eDNA depends on environmental parameters as well as on the organism’s characteristics. For example, detection from lentic waters is more straightforward than from lotic waters [13]. eDNA can be transported with currents and along rivers [14, 15], and occurs primarily close to the DNA source when water flows are low [15, 16]. Based on filtration experiments, it has been suggested that cells and mitochondria in the water are the major source for the eDNA signal in the case of carp [17]. Feeding rates as well as fish age are correlated with the amount of eDNA shed [18, 19]. The stability and, thus, detectability of eDNA depends on environmental parameters such as pH, radiation, and temperature [20–23] as well as on the species investigated [14]. eDNA decays exponentially [21] but can remain detectable for days up to months depending on the conditions. Finally, environmental samples contain substances that interfere with PCR [24]. For example, humic acids are released by decaying leaves in autumn and prevent amplification [15]. An eDNA assay must therefore be carefully designed to fit the species, sampling site, and sampling conditions of interest.

Given the above-mentioned patchy distribution, degradability, and dilution effects, several aspects need to be taken into account when designing an assay to detect Ponto-Caspian gobies at their primary introduction sites (harbors) and along their spreading routes (rivers). In harbors, Ponto-Caspian gobies live at ~4m depth. Considering the localized occurrence of eDNA, sampling the water surface may lead to false negatives. Also, harbor water is heavily disturbed by shipping during work days, which may impact the stability or availability of environmental DNA. Further, Ponto-Caspian gobies presumably prefer slow-flowing water [25], and eDNA approaches are more sensitive in slow-flowing or lentic environments [13]. Sampling sites with strong currents may produce false negatives because Ponto-Caspian gobies might be absent, or the signal might be diluted beyond detection [26]. Finally, an eDNA assay against Ponto-Caspian gobies should detect invasive goby species, but no native species and no other gobies, since Ponto-Caspian goby ranges overlap with those of native goby species, for example in the Baltic.

We have optimized eDNA protocols and sampling techniques along these lines and present a specific and sensitive PCR assay to detect Ponto-Caspian gobies from river water samples. We present primers specific for round goby *Neogobius melanostomus*, for bighead goby *Ponticola kessleri*, and for the entire group of all five invasive Ponto-Caspian goby species. We demonstrate that sampling depth matters. We find that the choice of DNA extraction and PCR protocol both have tremendous impact on the detectability of Ponto-Caspian gobies. We demonstrate an overwhelming effect of PCR inhibition on signal intensity. Finally, we use the assay to detect the invasion front of Ponto-Caspian gobies in one of the largest rivers of Europe, the Rhine.

## Methods

### Ethics statement

Field samplings performed in this study were authorized by the national office for energy and environment Basel Stadt (permit # GS-18-07-01). Sampling procedures were specifically approved as part of obtaining the field permit. Fishing does not require approval by an animal ethics committee if animals are sacrificed on site.

### Primer design

To design species-specific primers, the mitochondrial cytochrome B sequences from Ponto-Caspian gobies, other goby species, and from native Swiss fish were retrieved from the NCBI Database and aligned using Clustal Omega. Sites with unique sequence characteristics in the targeted species, in particular at the 3’ end of potential primers, were identified. For all Ponto-Caspian gobies, round goby *N*. *melanostomus*, and bighead goby *P*. *kessleri*, specific 16–20 nt long primers were designed to amplify fragments of 125, 85, and 200 bps, respectively. Alternative forward and/or reverse primers were designed by shifting the binding sites one or two nucleotides. The sequences and the alignment with primer binding sites marked can be found in S1 Supplementary Material. Primer sequences are, for the Ponto-Caspian goby primers: SL_eDNA_Gobies_F1, CCTCTAACATTTCTGCC; SL_eDNA_Gobies_F2, CCCTCTAACATTTCTGCC; SL_eDNA_Gobies_R1, GACGAGAAGGCTGTGGC; for the *N*. *melanostomus* primers: SL_eDNA_NM_F1, TATGTGATGATCGGACAGC; SL_eDNA_NM_F2, CTATGTGATGATCGGACAGC; SL_eDNA_NM_R1, GTTCTCTAGTCAGCTCGCT; and for the *P*. *kessleri* primers: SL_eDNA_PK_F1, ACTAGGCCTATGCCTG; SL_eDNA_PK_F2, CTACTAGGCCTATGCCTG; SL_eDNA_PK_R1, TAGTCCTCGTCCAATATGC; SL_eDNA_PK_R2, ATAGTCCTCGTCCAATATGC. The relative performance of the individual reverse and forward primers was tested using conventional PCR (see section “PCR conditions”) on 5 ng of pure *N*. *melanostomus* or *P*. *kessleri* DNA. Since our main goal was to provide a universal goby assay that is useful for all five invasive goby species, we decided to continue our experiments using the Ponto-Caspian goby primer pair. Nonetheless, we provide *N*. *melanostomus* and *P*. *kessleri* primers as a resource for other researchers.

### PCR conditions

Two types of PCR protocols were used throughout this paper. A conventional PCR protocol was used to determine the role of inhibition on signal intensities, and was also tested on environmental samples. The conventional PCR protocol consisted of an initial five-minute 95°C denaturation step, followed by various numbers of cycles (indicated below) of 60 seconds denaturation at 95°C, 30 seconds of annealing (temperatures indicated below), and 30 seconds of extension at 72°C. This was followed by a seven-minute final extension step at 72°C, and by cooling of the samples to 10°C. Annealing temperatures were 60°C for the Ponto-Caspian goby primers and 64°C for *N*. *melanostomus* and *P*. *kessleri* primers.

Since this protocol did not work stably on environmental samples, we tested two PCR protocol modifications. To alleviate potential PCR inhibition, which is common in environmental samples [15], we added BSA as an anti-inhibitory reagent upon personal recommendation by Kristy Deiner, EAWAG [27]. To increase sensitivity and specificity, we tested a touchdown PCR protocol. During the first cycles of a touchdown PCR, the annealing temperature is maintained above the melting temperature of the primers being used. During the subsequent cycles, annealing temperature decreases stepwise towards a more permissive annealing temperature. Any difference in melting temperature between correct and incorrect annealing will produce an exponential advantage of the correct template. In such a way, touchdown protocols increase the specificity, sensitivity and yield of PCR reactions [28]. We used the following cycling conditions for touchdown PCR: After an initial denaturation step of 5 minutes at 95°, a first set of 15 cycles was carried out. These 15 cycles started with 1 minute denaturation at 95°C, 30 seconds of annealing at 65°C, and 15 seconds of extension at 72°C. In each of these 15 cycles, the annealing temperature was lowered by 1°C compared to the previous cycle. Then, a second set of 35 cycles was carried out, with 30 seconds of denaturation at 95°C, 30 seconds of annealing at 50°C, and 15 seconds of extension at 72°C. The touchdown protocol finished with a 7 minutes extension step at 72°C followed by cooling to 10°C. The touchdown protocol was used to determine primer performance, primer specificity, and to test environmental samples.

All PCRs were carried out using Illustra Puretaq RTG PCR Beads 0.2, Fisher Scientific, 10678095. These beads consist of polymerase, buffer, and dNTPs, which are pre-aliquoted to PCR tubes in dried pelleted format as small white beads. Upon addition of water (or of a master mix containing the desired amounts of water, primer, and template), the beads dissolve to yield a ready-to-go PCR reaction. 3 μl of the environmental DNA preparation or of the indicated negative and positive controls were used as template. 0.5 μl of the forward and of the reverse primer (10 μM stock solution) were added to the PCR reactions. 1.25 μl of BSA (Molecular Biology Grade (20mg/ml), 12 mg, BioConcept, B9000S) was added as an anti-inhibitory agent whenever indicated.

### Primer specificity tests

**Fig 1:**
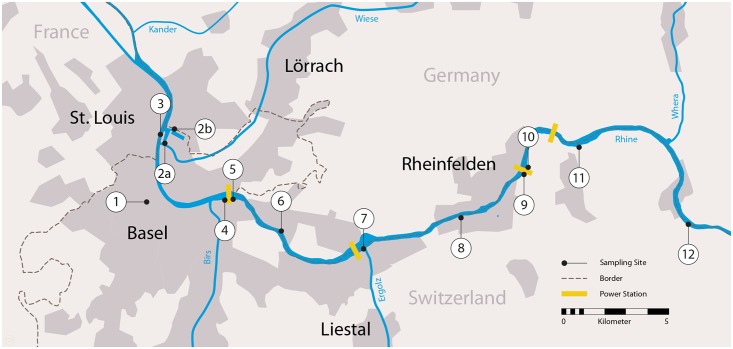
Sampling sites. Sampling sites were distributed along 36 km of the High Rhine in Switzerland between Basel (most downstream locations: sites #2a, #2b, and #3) and Mumpf (most upstream location: site #12). For detailed coordinates of sampling sites, see Table 1. In 2013, Ponto-Caspian gobies were reported, at very low densities, at a location midway between site #7 and site #8. Yellow bars, hydropower stations. Expansion of River Rhine opposite of site #7: former side arm of the river, today natural reserve. The map is oriented from North (top) to South (bottom).

We tested the specificity of Ponto-Caspian goby, *N*. *melanostomus*, and *P*. *kessleri* primer pairs on Ponto-Caspian goby samples, on all native Swiss fish we could obtain samples for, as well as on several goby species. Round goby *N*. *melanostomus* and bighead goby *P*. *kessleri* samples were obtained with minnow traps from the harbor (47°35'14.7"N 7°35'36.5"E) at sampling sites #2a and #2b (Fig 1), anesthetized with clove oil, and frozen at -20°C until lysis. Tissue samples from other fish were kindly provided by the group of Ole Seehausen (EAWAG, Zurich, Switzerland). These fish were sampled during a large Swiss lake monitoring project at various sites in Switzerland, and preserved in ethanol at -20°C. All DNA extractions were performed with the Blood and Tissue Kit from Qiagen (Catalogue number 69504). Tested fish species included *Neogobius melanostomus*, *Ponticola kessleri*, *Babka gymnotrachelus*, *Neogobius fluviatilis*, and *Proterorhinus semilunaris* for the invasive Ponto-Caspian goby species, *Pomatoschistus pictus*, *Aphia minuta*, *Pomatoschistus microps*, and *Pomatoschistus minutus* for the other goby species, and *Anguilla anguilla*, *Cottus gobio*, *Gasterosteus aculeatus*, *Gymnocephalus cernuus*, *Perca fluviatilis*, *Lota lota*, *Esox lucius*, *Barbatula barbatula*, *Cobitis bilineata*, *Blikka bjoerkna*, *Abramis brama*, *Alburnoides bipunctatus*, *Alburnus alburnus*, *Barbus barbus*, *Coregonus nasus*, *Cyprinus carpio*, *Leucaspius delineatus*, *Squalius cephalus*, *Leuciscus leuciscus*, *Phoxinus phoxinus*, *Rutilus rutilus*, *Scardinius erythrophthalmus*, *Tinca tinca*, *Thymallus thymallus*, *Salmo trutta*, and *Lampetra planeri* for Swiss native fish species. As a positive control, we used Cytochrome Oxidase I (COI) barcoding primers F1 and R1 on each species [29]. The touchdown PCR protocol with BSA was used for the specificity tests. It has to be noted that the touchdown protocol is suboptimal for the performance of COI barcoding primers, but was used nonetheless to ensure comparable and relevant conditions across the data set.

### Water sampling in the field

**Fig 2:**
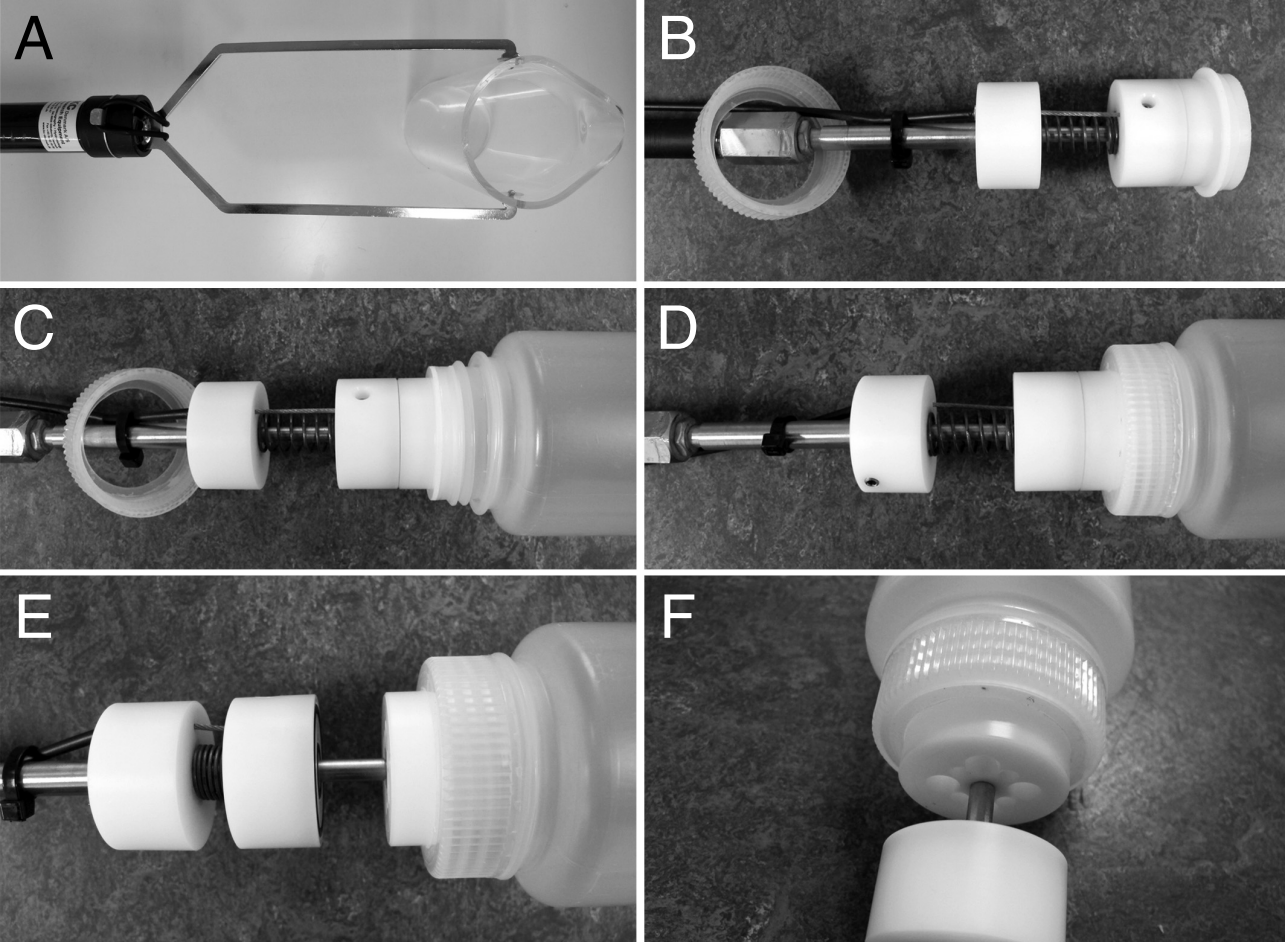
Water sampler. A commercially available water sampler was adapted for sampling benthic water. (A) Sampler as purchased. (B) Modified sampler, with plunger closed by a spring. (C) Sampler with bottle attached, plunger closed. (D) Bottle screw cap closed, plunger closed. (E) When the bottle touches ground, the plunger can be opened by pulling the attached cable. (F) When the plunger is opened, water can enter the bottle through holes in the bottle holder. See S2 Supplementary Material for building instructions.

Ponto-Caspian gobies are benthic fish that do not generally ascend into the water column, except when they drift as larvae [30, 31]. We therefore assumed that DNA concentrations would be highest in the bottom water layers. These layers can be sampled with water column samplers, which consist of a closed reservoir that can be inserted into the water and flooded at the desired depth. Most commercially available water column samplers are, however, difficult to clean in the field between individual samplings, and/or expensive, and/or not very flexible in handling in different environments. We therefore adapted a commercially available surface sampler for our purpose. The sampler from KC Denmark (24.001 Water Sampler with telescope handle) was thoroughly modified to replace the acryl beaker at the distal end with a holder for re-useable and screw-cap plastic bottles so that a 500 ml Nalgene plastic bottle could be plugged tightly to the bottle holder at the tip of the device. A plunger with a cable attached sealed the bottle during insertion into the water. Technical details of the modifications are described in S2 Supplementary Material. Once the bottle touches ground, the cap could be opened by pulling the attached cable, so that water could enter the bottle through holes in the bottle holder (Fig 2). Successful sampling could be judged from ascending water bubbles. The filled sampler was then lifted out of the water. The bottle was unscrewed from the holder and closed with the original screw-cap lid. Then, a new bottle was attached for the next sample. All environmental samples were taken using this device. After sampling, filled bottles were cooled on ice during transport (by car or in a two-wheeled bike trailer) and immediately processed after return to the laboratory.

Field sampling sites were chosen according to the following characteristics: 1) association with leisure boat shipping or commercial shipping, the suspected main long distance vectors for invasive gobies (Kalchhauser et al, submitted; [3]); 2) moderate water flow, since invasive gobies are benthic and sedentary fish preferring slow waters; 3) data or expectations on the presence/absence of invasive gobies, since this would allow us to include positive and negative controls. Sites include a goby-free carp pond on the University campus as negative control (#1), two sites in the cargo harbor that are known to be populated densely by *Neogobius melanostomus* and less densely by *Ponticola kessleri* as positive controls (#2a and #2b), a marina outside the cargo harbor (#3), two opposed sites at the commercial shipping gate at the Birsfelden hydropower dam (#4 and #5), the tank ship harbor (#6), a marina near Augst which we later confirmed to be goby-free by minnow traps and snorkeling (#7), the mooring site of a popular passenger boat “Froschkönig” (#8), the downstream and upstream leisure boat elevator station at the hydropower station Rheinfelden (#9 and #10), and two marinas further upstream (#11 and #12). Sites are indicated in Fig 1. Coordinates, characteristics and further information on the sites are given in Table 1. Goby densities were expected to decrease with increasing distance from the commercial harbor (sites #2a and #2b) because the invasion front is slowly expanding upstream in the High Rhine. No gobies were expected beyond the Rheinfelden dam (#9 and #10), because the putative major vector, commercial ship traffic, does not extend beyond this dam.

**Table 1:** Sampling sites.

Site #	Site	Description of site	Water flow*	Coordinates
1	Carp pond (negative sampling control)	Shallow concrete pool, 5x20 m	-	47.558198, 7.583823
2a	Harbor Kleinhüningen, harbor basin 1 (positive sampling control).	Commercial harbor, turning over containers and solid goods.	-	47.583065, 7.588442
3b	Harbor Kleinhüningen, harbor basin 2 (positive sampling control).	Commercial harbor, turning over containers and solid goods.	-	47.587457, 7.593517
3	Marina Kleinhüningen	Harbor for private leisure boats and yachts just outside harbor Kleinhüningen in the main river bed.	++	47.586269, 7.588172
4	Water gate Birsfelden, mainland bank	Water gate for large commercial vessels at the hydropower dam Birsfelden, sampled from the shore.	-	47.558409, 7.626634
5	Water gate Birsfelden, island bank	Water gate for large commercial vessels at the hydropower dam Birsfelden, sampled from the artificial island supporting the dam.	-	47.558975, 7.627701
6	Harbor Auhafen	Commercial harbor turning over petroleum products.	++	47.540868, 7.661943
7	Marina Augst	Harbor for private leisure boats and yachts near the town Augst, situated in a small bay separated from the main river.	-	47.537826, 7.713531
8	Mooring site "Froschkönig"	Passenger boat mooring site, situated in the main river bed.	+++	47.551201, 7.772322
9	Rheinfelden boat passage, downstream	Site to lift leisure boats travelling upstream out of the water, to lift them over the hydropower dam Rheinfelden.	-	47.566906, 7.810632
10	Rheinfelden boat passage, upstream	Site to lift leisure boats travelling downstream out of the water, to lift them over the hydropower dam Rheinfelden.	+	47.569899, 7.814248
11	Marina Möhlin	Harbor for private leisure boats and yachts near the town Möhlin, situated in the main river bed.	+	47.580878, 7.841163
12	Marina Mumpf	Harbor for private leisure boats and yachts near the town Mumpf, situated in the main river bed.	++	47.547865, 7.914662

* subjective and relative impression during water sampling

Before sampling the field sites, we thoroughly tested a number of negative controls to find a good negative control and to confirm our ability to work cleanly, without cross-contamination. To this end, we tried to amplify bands using Ponto-Caspian goby, *N*. *melanostomus*, and *P*. *kessleri* primers from ultrapure water, from tap water subjected to the filtration and Qiagen kit DNA isolation protocol (described below), from tap water rebottled in the harbor (site #2b) and subjected to the DNA isolation protocol, and from samples from the uninvaded carp pond (site #1) that were taken with the sampler before and after a field trip to an invaded site and subjected to the DNA isolation protocol.

Before sampling all field sites, we also tested whether the degree of disturbance of the water column or sampling depth would have an influence on detection. We had two assumptions on the behavior of goby eDNA in harbors. First, we figured that DNA might be differentially distributed in the water column, considering that we are dealing with a strictly benthic species and considering reports on the patchy distribution of eDNA in water bodies. Second, given that eDNA stability depends heavily on environmental parameters, we thought that ship-mediated perturbations of the harbor water may impact DNA concentrations, for example by providing oxygen to microorganisms degrading organic compounds. To test the effect of time and depth, we took samples from the bottom (4m depth) and from the surface at site #2b, in triplicates. We also took bottom samples on weekdays, when shipping is ongoing and the water column is heavily disturbed, and on the work-free weekend, when no shipping occurs.

### Good laboratory practice

eDNA has been facing skepticism because in some cases, eDNA is more sensitive than conventional methods and indicates the presence of a species while catch efforts are unsuccessful [11, 32–34], which impedes independent confirmations of eDNA results. Also, cross-contaminations may occur in a laboratory where pre- and post-PCR steps are not adequately separated, or where fish samples are handled in the same room as eDNA samples.

We took a number of measures to avoid and to control for contamination. When sampling at a certain sampling site was finished, and the last bottle was removed from the sampler, the sampler was decontaminated to remove environmental DNA traces before sampling was continued at a different location. To this end, the sampler tip without bottle attached was inserted into a bottle with sodium hypochlorite (1:5 dilution of a solution containing 13% active chlorine, Chem-Lab, CL00.1410), shaken, removed from the sodium hypochlorite bottle, and wrapped in household tissue while still moist. The sampler and tissue were then covered with a plastic bag for transport to the next sampling site. There, the bag and the tissue soaked with bleach were removed, and the sampler was rinsed with ddH_2_O to remove residual bleach prior to the next sampling.

All equipment used for DNA isolation that was re-used, such as filter housings or glass petri dishes, were decontaminated between isolations and used only once during each DNA isolation run. After each use, items were cleaned by incubating for 30 minutes in sodium hypochlorite (see above). They were then rinsed with ddH_2_O, air-dried, and kept in a sealed box until next use.

Sampling sites were visited in an ordered fashion: Samples were taken from expected least to expected highest concentration. The negative control site, the carp pond, was sampled last during each field trip to control for sampler contamination and for carry-over between sites. In the laboratory, incoming samples were handled in a separate room up to the lysis step, and were again processed from expected lowest to expected highest concentration, with negative control being processed last. Potentially contaminated equipment such as petri dishes and filter housings was immediately removed from the bench after use. Post-PCR steps were performed with dedicated pipettes which were not used for any other purpose. Also, all obtained bands were sequenced by a commercial provider of sequencing services, Microsynth AG (http://www.microsynth.ch/).

### DNA extraction from environmental samples

We screened the literature for suited DNA extraction protocols, and identified 60 publications, which differ widely in their recommendations. We analyzed the protocols step by step, and identified a wide variety of approaches. Sampling volumes vary from 15 ml to several liters. Samples are frozen or processed immediately. DNA extraction methods are based on precipitation or filtration. Filter types include nylon filters, cellulose filters, and glass fiber filters. We compiled a detailed summary of protocols available in 2014 in S1 Table.

For our assay, we chose to test three different DNA extraction methods that we adapted from the three approaches dominating literature: ethanol precipitation followed by Qiagen Blood and Tissue kit extraction, adapted from [35]; filtration followed by Qiagen Blood and Tissue kit extraction as in [27]; and filtration followed by CTAB buffer extraction and precipitation as in [36]. We decided to use glass fiber filters after personal communication with Kristy Deiner, EAWAG, Switzerland. We sampled nine water samples from a heavily invaded site in the local commercial harbor (site #2b) and assigned three each to one of the extraction methods to determine optimal conditions. For experimental details of each of those protocols, see S3 Supplementary Material.

### Correlating signal intensity with abundance estimates

To get an idea whether the relative strength of the eDNA signal would approximate relative fish density, we compared eDNA amplification results to catch data. Catch data from the harbor were available thanks to a long-term monitoring effort for a research project on population dynamics. Between 2012 and 2014, invasive goby populations in the harbor Kleinhüningen in Basel (#2a and #2b) were monitored with minnow traps. Traps were baited with dry dog food and were deployed at the harbor bottom and emptied every 3–4 days during specific monitoring windows that were identical at all sites. During this monitoring effort, less invasive gobies were caught at site #2a in the outer harbor basin than at site #2b in the inner harbor basin. Environmental DNA samples were collected at these two sites in triplicate from the bottom. Goby DNA was amplified using the touchdown PCR protocol with BSA, and band intensities were compared with the catch data.

### Inhibition testing

To see whether PCR inhibition would affect band intensities, we conducted a PCR inhibition testing experiment. We used an unrelated template, the plasmid pBlueskript SK+, together with the standard vector-specific primers T3 and T7 (T3, ATTAACCCTCACTAAAGGGA; T7, TAATACGACTCCTATAGGG). Using the conventional PCR protocol with an annealing time of 30 seconds and an annealing temperature of 50°C, the amount of DNA template and the number of PCR cycles at which the band intensities reflected amplification efficiency were determined by testing serial dilutions of the plasmid DNA and different numbers of PCR cycles. We found that 22 cycles on 0.1 ng of plasmid DNA represented parameters where more or less DNA, or more or less cycles, respectively, would result in more or less intense bands, and used these parameters for the inhibition testing PCR. We then set up replicates of this inhibition testing PCR with and without BSA, and spiked the plasmid DNA with 3 μl of environmental samples from sites #1, and #2b to #8, 70% ethanol as a positive control for inhibition, ddH_2_O as a negative control not causing inhibition, and an extraction kit control to control for inhibition potentially caused by the Qiagen Blood and Tissue kit (3 μl of an unrelated *N*. *melanostomus* DNA preparation). The band intensities were compared to check for PCR inhibition.

## Results

### Primer performance tests

Although differences in sequence between individual primers were minute, we could clearly identify an optimal primer pair for Ponto-Caspian goby (goby F1 and goby R1), for round goby *Neogobius melanostomus* (NM F1 and NM R1), and for bighead goby *Ponticola kessleri* detection (PK F2 and PK R1). For example, the primer pair PK F1—PK R1 was much less sensitive than the primer pair PK F2 –PK R1, although the primers differ only by two additional nucleotides at the 5’ end of primer F2 (S1 Fig).

**Fig 3:**
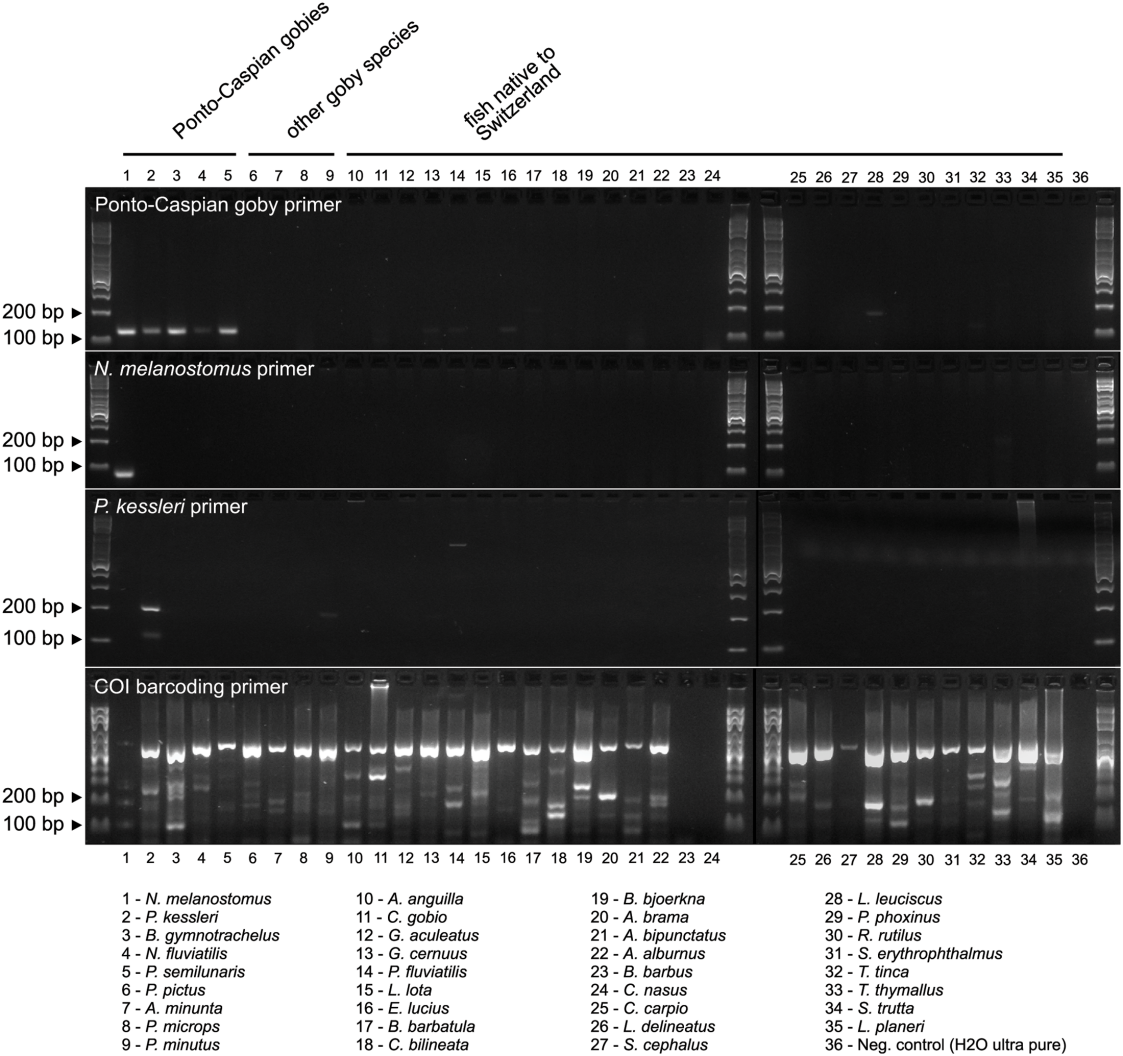
Primer specificity. Ponto-Caspian goby, round goby *N*. *melanostomus* and bighead goby *P*. *kessleri* primers were tested on samples from Ponto-Caspian gobies, from other goby species, and on fish native in Switzerland. Cytochrome Oxidase I (COI) was amplified as positive control from all samples.

All primer pairs were specific for the species they were designed for. For bighead goby *P*. *kessleri* and round goby *N*. *melanostomus* primer pairs, we did not observe relevant cross-amplification with other Ponto-Caspian gobies, other gobies, or Swiss native fish, with the exception of weak cross-reactivity with *P*. *minutus* for the *P*. *kessleri* primers. For the Ponto-Caspian goby primer pair, we observed weak cross-reactivity with *G*. *cernuus*, *P*. *fluviatilis*, *E*. *lucius*, and *T*. *tinca*, and an off-size band with *L*. *leuciscus* (Fig 3). However, when sequencing the products obtained from environmental samples, they always were identified as *N*. *melanostomus*, which indeed is the locally dominant invasive Ponto-Caspian goby species.

### PCR protocol tests

**Fig 4:**
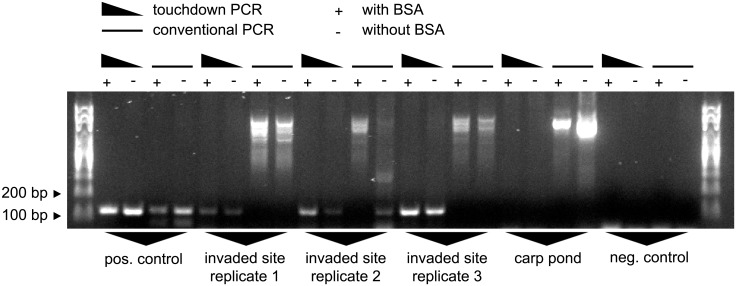
PCR protocol tests. Three water samples were taken from the invaded commercial harbor at site #2b. DNA was isolated, and each sample was subjected to conventional PCR and touchdown PCR with or without BSA. Primers: Ponto-Caspian goby primers. Experimental control: Carp pond (site #1) sampled after the field trip. Positive control: 5 ng *N*. *melanostomus* DNA as template. Negative control: ultrapure water as template.

When comparing touchdown PCR and conventional PCR, with and without BSA, on samples taken from the heavily invaded site #2b in the harbor, we found that touchdown PCR is highly superior to conventional PCR both in terms of sensitivity and specificity. Conventional PCR failed in 5 out of 6 cases to amplify the correct product and suffered from unspecific amplification of background bands. Touchdown PCR, on the other hand, resulted in one single clear band on the same samples. Importantly, BSA was a crucial additive that increased band intensities (Fig 4).

### Extraction protocol tests

**Fig 5:**
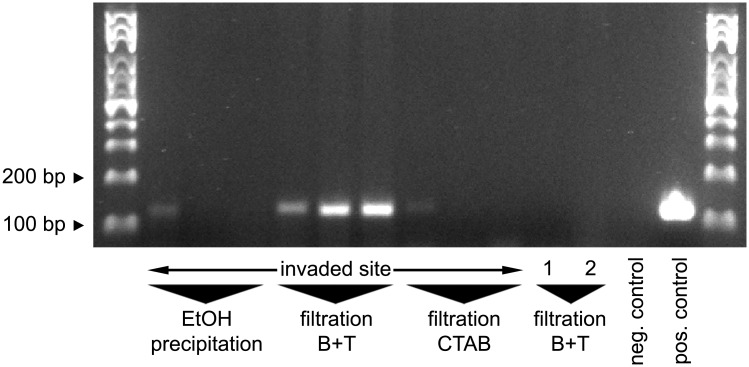
Extraction method tests. Nine samples from a heavily invaded site at the local commercial harbor (site #2b) were assigned randomly to one of three extraction methods (EtOH precipitation, glass fiber filtration followed by Qiagen Blood and Tissue (B+T) kit lysis, and glass fiber filtration followed by CTAB lysis). Control samples were processed with glass fiber filtration followed by Qiagen Blood and Tissue kit lysis. Control sample 1: tap water, re-bottled at the sampling site. Control sample 2: uninvaded carp pond (site #1), sampled after the field trip. Primers: Ponto-Caspian goby primers. Positive control: 5 ng *N*. *melanostomus* DNA as template. Negative control: ultrapure water as template.

We compiled a large body of literature on eDNA methodology (S1 Table) and found that there was no such thing as a standard protocol that worked well for everyone. We found that generally speaking, every single step, from sample volume to sample storage to sample processing to DNA isolation to amplification, depended on the setting and purpose of the respective study. However, three approaches toward DNA isolation dominated the literature: (1) EtOH precipitation, (2) filtration followed by lysis with a kit, and (3) lysis in CTAB buffer with or without filtration. Types of filters used varied widely in pore size and material. When contrasting the three most frequent isolation approaches on our samples, we found that EtOH precipitation, as well as glass fiber filtration followed by CTAB lysis, allowed only very weak detection in 1 of 3 samples from the heavily invaded site #2b. In contrast, glass fiber filtration followed by Qiagen Kit lysis resulted in a distinct signal in 3 of 3 samples (Fig 5).

### Negative controls, good laboratory practice and decontamination

**Fig 6:**
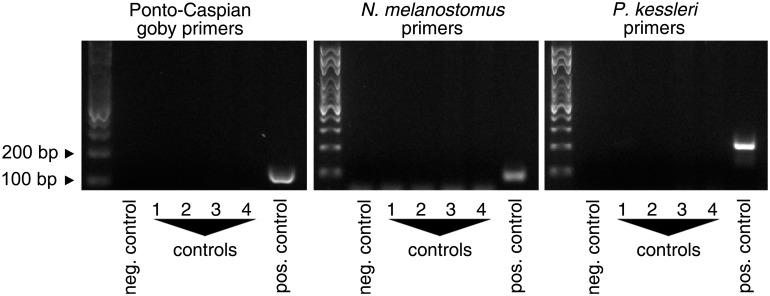
Negative Controls. None of the primer pairs amplifies bands on ultrapure water (negative control), or on DNA isolated from tap water (1), tap water rebottled in the harbor (2), samples taken from an uninvaded carp pond (site #1) before (3) or after (4) a field trip to an invaded site (site #2b). Positive control: 5ng of DNA from *Neogobius melanostomus* (Ponto-Caspian goby primers, *N*. *melanostomus* primers) or from *Ponticola kessleri* (*P*. *kessleri* primers) as used as template.

In a next step, we tested a variety of negative sampling controls. We never observed amplification from tap water samples, from tap water samples that were transported to the sampling site and re-bottled under field conditions, and from samples from an uninvaded carp pond, which we sampled before and after field trips to invaded sites using the same gear as on the field trip (Fig 6). Based on those results, we decided to sample the uninvaded carp pond (site #1) as a last step of each field trip, because it would not only serve as a negative control, but also allow us to control for cross-contamination during the field trip. Any signal in this sample would indicate carry-over of material between sampling sites. We also always included a no-template-reaction in every set of PCRs to control for contamination of PCR reagents and laboratory equipment.

### Sampling depth and time tests

**Fig 7:**
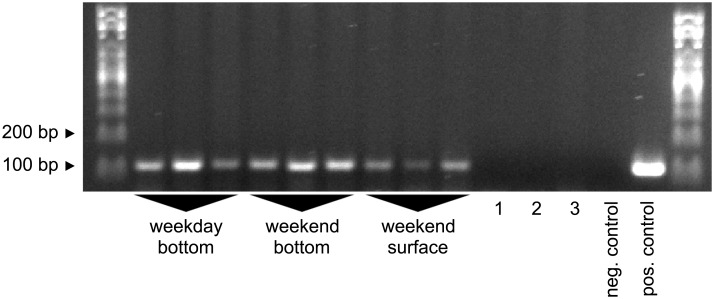
Sampling depth impacts signal strength. Nine samples were collected from the invaded commercial harbor on Wednesday (weekday) or Sunday (weekend) and from the surface or the bottom of the harbor at site #2b. 1–3: experimental controls. 1: Carp pond (site #1) sampled after weekday field trip. 2: Carp pond (site #1) sampled after weekend field trip. 3: Tap water, re-bottled during weekend field trip. Primers: Ponto-Caspian goby primers. Positive control: 5ng *N*. *melanostomus* DNA as template. Negative control: ultrapure water as template.

To optimize sampling depth for the benthic goby species, we compared signal intensities from samples taken from 4m depth in the heavily invaded harbor at site #2b with samples taken from the surface of the same site. Indeed, we found that bottom samples gave stronger bands. We then continued to compare bottom samples taken on Wednesday (with ship traffic) with bottom samples taken on Sunday (without ship traffic), and found that both performed equally well (Fig 7).

### Testing for a relation between abundance and signal intensity

**Fig 8:**
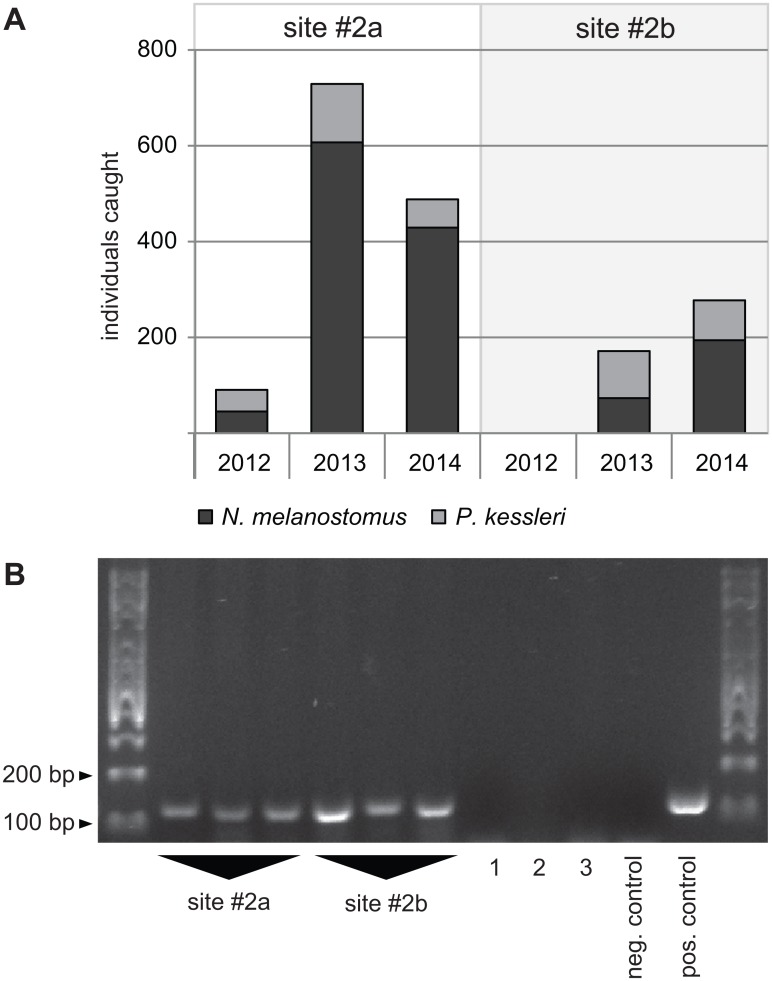
Abundance is not correlated with signal strength. (A) Abundance. Catch data from 2012 to 2014 for sites #2a and #2b. (B) Signal strength from the same site. 1–3: field controls. 1: tap water, rebottled in harbor, site #2a. 2: tap water, rebottled in harbor, site #2b. 3: carp pond (site #1) sampled after field trip. Primers: Ponto-Caspian goby primers. Positive control: 5ng *N*. *melanostomus* DNA as template. Negative control: ultrapure water as template.

We then went on to test whether signal intensities were related to abundance. At site #2a, we caught 267 round goby *N*. *melanostomus* and 181 bighead goby *P*. *kessleri* since 2012. At site #2b, we caught 1081 round goby *N*. *melanostomus* and 226 bighead goby *P*. *kessleri* during the same time. However, signal intensity was not related to the catch data. Although we had caught less gobies at site #2a, the signal was stronger in environmental water samples taken at this site (Fig 8).

### Detection of the goby invasion front from river samples

**Fig 9:**
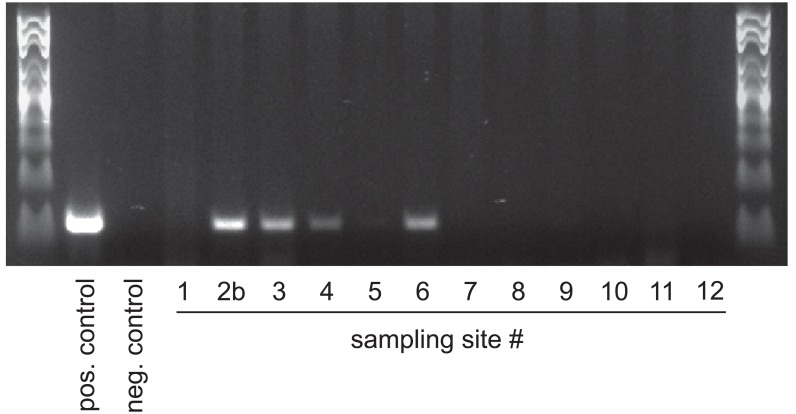
Ponto-Caspian gobies can be detected from river water. Numbers correspond to sampling sites in Fig 1, with site #1 as experimental control from the carp pond, and sites #2b-12 as field samples extending upstream from the heavily invaded harbor (site #2b). Primers: Ponto-Caspian goby primers. Positive control: 5ng *N*. *melanostomus* DNA as template. Negative control: ultrapure water as template.

**Fig 10:**
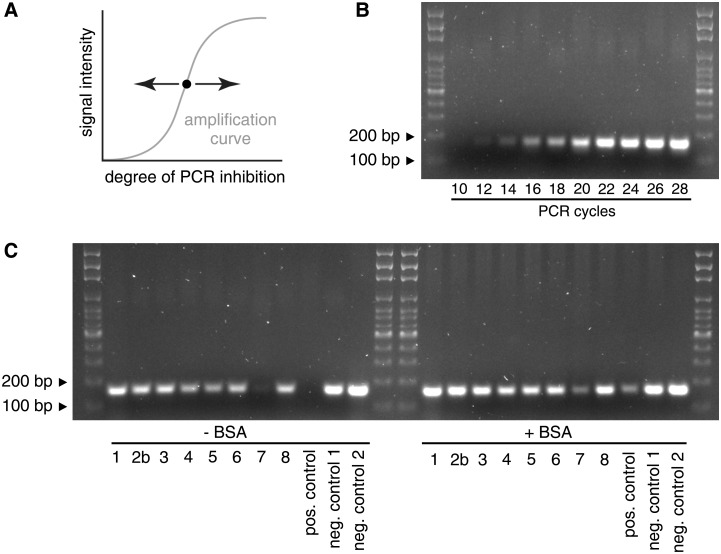
Testing environmental samples for PCR inhibition. (A) Conceptual illustration of an inhibition testing PCR. An inhibition testing PCR is set up so that its endpoint is placed in the linear phase of a PCR amplification. Changes in the degree of PCR inhibition, template or cycle number will therefore result in changes in signal intensity. For a PCR with an endpoint as indicated by a black dot, more inhibition would result in lower signal intensity, and less inhibition would result in higher signal intensity. (B) Determination of the optimal cycle number for the inhibition testing PCR. Between cycles 10 to 28, the amplification curve is in the dynamic range. (C) Inhibition tests using environmental samples. Three microliters of the indicated environmental sample were added to the inhibition testing PCR, and PCR was run for 22 cycles in the absence (left) or presence (right) of BSA as an anti-inhibitory agent. Positive control, 70% EtOH. Negative control 1, ddH_2_O. Negative control 2, independent DNA preparation isolated with the Qiagen Blood and Tissue kit.

After optimizing PCR and sampling conditions, we tested the protocol on environmental samples taken along a river stretch suspected to contain the invasion front. From these samples, we could clearly detect the presence of Ponto-Caspian gobies up to sampling site #6, which is located 10.5 km upstream of the heavily invaded commercial harbor (sites #2a and #2b). Samples further upstream (#7—#12) were negative. Signal intensities of positive samples, however, varied widely, even between closely spaced sites (Fig 9). We therefore tested the capacities of these samples to inhibit PCR reactions. When adding aliquots of the environmental samples to an inhibition testing PCR, we found that the sample from site #7 would inhibit PCR to the same extent as 70% ethanol. In contrast, samples from sites #1, #2b—#6 and from site #8 would have no or only mild effects on signal intensity. Importantly, adding BSA alleviated the inhibitory effect in all cases (Fig 10).

## Discussion

In this paper, we described a protocol for the detection of Ponto-Caspian gobies from river water samples. The protocol was based on glass fiber filtration and subsequent DNA extraction with the Qiagen Blood and Tissue kit, and on a touchdown PCR with added BSA as an anti-inhibitory agent. We presented primers specific for bighead goby, round goby, or all five invasive Ponto-Caspian goby species. We successfully applied the protocol to samples from a large stream and outlined the invasion front of Ponto-Caspian gobies in the Rhine. In the following, we discuss individual aspects of the protocol and of our approach.

### Primer design, primer performance and primer specificity

When designing our primers, we chose a species-specific and species-group-specific approach rather than a community sequencing approach. Our objective was to provide an assay that was independent of access to Next Generation Sequencing. To rule out significant cross-reactivity of the primers with other species, we used binding sites that were as little conserved as possible with native species, and we tested the primers on as many native fish samples and other goby species as we could. Our results validated this approach. We observed very little cross-reactivity. Also, all bands obtained from field samples were indeed *N*. *melanostomus*.

Yet, it is important to keep in mind that the specificity tests were performed on pure and concentrated DNA preparations obtained from ethanol-preserved tissue samples. The primers may potentially display different specificities on environmental DNA, which is present at low concentrations, degraded to various degrees, and subject to PCR inhibition. We therefore recommend sequencing obtained bands routinely. Also, we would be happy to see other goby species as well as water samples from other invaded areas tested with those primers.

### PCR conditions

When we started the project, we tried a number of conventional PCR protocols. However, only after adjusting PCR conditions to a touchdown protocol, and after adding an anti-inhibitor to the PCR mix, we were able to detect Ponto-Caspian gobies in river samples. In particular, the touchdown protocol was a key feature of the assay. The same sample would yield no band or smears with a conventional approach and a clear and distinct band with the touchdown protocol. We take this as an indication for the presence of a wide variety of other, similar sequences in the environmental samples that cause aberrant primer binding. It seems like the conditions in environmental samples promote mis-priming, even when primers are selected for specificity.

### Extraction protocol tests

We scanned a broad body of literature when searching for a suited protocol to extract DNA from environmental samples, and decided to test three approaches: precipitation, glass fiber filtration and kit lysis, or glass fiber filtration and CTAB buffer lysis. In our hands and for our samples, glass fiber filtration followed by Blood and Tissue kit extraction [27] clearly outperformed the other two methods. We recommend including this protocol in the testing phase when establishing an eDNA-assay, since it seems fairly robust. Also, glass fiber filters with their variable pore sizes are likely able to catch very different types of particles, in contrast to single-pore-size filters that are highly selective. This said, many other extraction protocols available in the literature were not tested in this paper, and those tested unsuccessfully for our purposes may work excellently after further protocol adaptations or when using different types of samples.

### Assay sensitivity

We cannot exclude that invasive gobies were present at sampling sites #7 to #12, where our assay was negative. We do not know the limit of detection of the assay in terms of minimal number of fish per unit water, or per area that can be detected. We consciously refrained from testing the limit of detection. Any lab-based approach, such as keeping invasive gobies in fish tanks and sampling the fish tank water, would fail to account for field conditions such as variable levels of inhibition or depth effects. Any field-based approach to determine limits of detection would require knowledge about exact densities of fish in a number of locations to allow the establishment of a robust signal-to-density relationship. Such an approach, even if it were possible to get such exact density measurements, would still not take different inhibition levels at individual sampling sites into account, which we have shown to be highly variable. In this light, any realizable test for limit of detection would have very limited relevance for the field outside the area where it was established.

However, we can imagine a number of modifications to render the assay more sensitive in the future. For example, the elution volume from the DNA extraction column could be reduced to achieve higher DNA concentrations. This would have to be balanced and controlled carefully, since lower dilution may have negative effects on detectability due to PCR inhibition [37]. Dilution is a valid approach to counteract PCR inhibition [37]. Also, water samples could be taken specifically during larval drift hours in the reproductive season in spring and summer [30]. When goby larvae are present in the water column, they are more likely to be included in the water sample, providing additional biological material and thus, detectable DNA, during the extraction. Also, fish are likely most active both in terms of movement and in terms of metabolism during the warm season, which may increase the amount of available DNA. An additional measure to increase sensitivity would be to subject every environmental sample to more than one round of PCR. This has been done before to reduce the number of false negatives [38]. Finally, sediment sampling may provide an alternative avenue. In some cases, sediment samples contained more, in others, less environmental DNA than water samples [16, 39]. Given that Ponto-Caspian gobies are benthic fish which occasionally hide in sandy grounds (own observation), eDNA concentrations may well be highest in sediments.

### Successful and specific detection of an invasive fish in lotic waters

eDNA assays have become more and more popular in aquatic research. Many technical aspects, such as the dynamics of eDNA in standing and flowing waters [15, 16, 18, 20–22], degradation rates [40] or shedding rates [18], have been investigated. Yet, examples for successful monitorings of specific fish in lotic freshwater environments are scarce. Proof of concept studies in flowing waters exist for *Misgurnus fossilis*, but with low detection rates in areas of known occurrence [10, 13], and for Asian carp [32]. All other approaches in running freshwaters have focused on amphibians or mammals [13, 41, 42] or co-detected fish during community sequencing approaches [27].

Admittedly, invasive gobies might represent an ideal case for eDNA monitoring. They might be comparably easy to detect, since they occur in high densities and in a defined river compartment (the bottom zone) that can be sampled in a targeted manner. In this compartment, water flow is lower, which may prevent dilution of environmental DNA and have a positive effect on detectability [15]. Also, invasive gobies’ guts are hardly ever empty [43], indicating continuous feeding, and these fish may thus shed comparatively large amounts of DNA in comparison to metabolically less active species.

Together, our results indicate the importance of taking the lifestyle of the target species into account when designing an aquatic eDNA assay. In particular, sampling depth matters in the case of a benthic fish species. On a side note, our results on sampling depth also indicate that community sequencing approaches should involve samples from as many different compartments and habitats as possible.

### Assay reproducibility

We always sampled in triplicates during the optimization steps, and found that replicates never conflicted about presence or absence when using the touchdown protocol with BSA. Signal intensity, however, varied widely between replicates, or also between different depths, or between closely spaced sites. For example, sampling sites #4 and #5 are directly facing each other at the inlet of the water gate at the Birsfelden hydropower dam. They are separated by only 70 m beeline distance, but differ strongly in signal intensity. It is known from carp that environmental DNA can be distributed in a highly patchy manner in the water [16]. Alternatively, inhibitors may have been present in sample #4 but not #5. This patchy distribution—be it of DNA or of inhibitor—needs to be taken into account in monitoring schemes. We recommend taking samples at dense intervals when trying to nail down an invasion front, or when monitoring presence/absence in vulnerable aquatic ecosystems.

### Signal intensity, fish abundance, and inhibition

We could not amplify invasive goby DNA from water samples taken at site #7. This was unexpected, since bighead goby *P*. *kessleri* had been caught a couple of hundred meters upstream from site #7 in 2013. However, we could confirm the absence of invasive gobies at site #7 by exposing a dog-food baited minnow trap for a total of two weeks in May 2015. Subsequent diving efforts were also negative. We therefore conclude that, while this result is somewhat unexpected, invasive Ponto-Caspian gobies are really absent at site #7. We do not know whether the upstream population still exists, but no eDNA reached the somewhat secluded marina at site #7, or the upstream population collapsed since 2013.

While we are confident in the presence/absence results obtained with our assay for the sampled sites, our results on inhibition as well as our comparison with catch data indicate that the assay in its present format is not suited for abundance estimates. It has been noted before that catch data and eDNA signal intensities are not necessarily correlated for aquatic species [44, 45]. In tanks, eDNA concentrations have been shown to reach an equilibrium point [46]. In heavily invaded commercial harbor basins, such an equilibrium may be reached. For most sites, however, our results indicate that signal intensity depends largely on the level of PCR inhibition, rather than on the amount of template present in the environmental sample.

On a more general note, abundance estimates from eDNA approaches should always be taken with a grain of salt. While quantitative eDNA assays for invasive species are something we all dream of, quantitative approaches are very hard to realize and to control for a number of technical and ecological obstacles. Determining the absolute abundance of aquatic species is often difficult in the field, which impedes the controlled development of such an assay on field samples. eDNA concentrations decline away from the source, which may result in mis-estimations of abundance if the DNA sources are not distributed homogeneously in the water body. The degree of PCR inhibition varies strongly between individual sampling sites, resulting in various degrees of underestimates of abundance. While inhibition could be partially controlled for by spiking each water sample with an unrelated standard PCR template prior to DNA extraction, other factors impacting signal intensity are just beyond our control. If the same amount of environmental DNA would degrade faster in one environment than in the other, abundance in the high-degradation environment would be underestimated. If the same amount of environmental DNA was present in easily purifyable form in one environment and in a difficult to purify form in another (for example in large particles versus free DNA), again, abundance estimates would be impacted. Depending on the season, daytime, or age, variations in fish metabolic parameters may impact shedding rates, so that the same number of individuals would yield a stronger or weaker signal in different seasons. For these reasons, a truly quantitative assay may remain illusory for some more time.

### Protocol simplicity

Monitoring institutions are often hesitant to adopt eDNA approaches because they lack the expertise and the equipment needed to perform the protocols. In this view, a major benefit of the presented protocol is its simplicity. The sampler can be built by any mechanic according to the instructions, can be transported in any car or on a bike, and can be cleaned in the field between samples. The PCR reagents are ready-to-use beads which can be stored at room temperature. The PCR protocol does not require quantitative real-time equipment or expertise. Although quantitative real-time PCR may be more sensitive than standard PCR [47], standard PCR is sufficient for standard monitoring purposes [48] and detects eDNA as reliably as qPCR or digital droplet PCR [49]. To further ease the implementation of the protocol, a very detailed summary of all steps is given in S4 Supplementary Material.

The most restrictive parameter we experienced during our experiments was transport capacities for water samples on ice during field trips, particularly when traveling by bike and small bike trailer. Alternative buffers, CTAB and Longmire’s, have been suggested to abolish the need for cold preservation [36]. In our hands, however, CTAB buffer was not the optimal choice. Although it complicates transport, we therefore recommend transporting samples on ice. Another bottleneck in our protocol was the same-day-filtering of all collected samples. When we collected more than 20 samples, filtering all samples after the field trip became challenging. In many studies, samples are therefore frozen to -80°C after the field trip and processed after thawing on ice on a following day (S1 Table). In a systematic survey, freezing negatively affected detection rates [50]. We did not test the effect of freezing systematically, but we occasionally froze samples (none of those were used for this paper). Signal intensities from these samples were comparable to those from samples processed immediately. Further tests may therefore indicate that samples can be frozen and do not need to be processed on the day they were sampled.

### Applications beyond monitoring distributions

Our aim was to present an eDNA tool for detecting Ponto-Caspian gobies from river samples. The presented primer pairs may however also be useful for gut content analyses. Preliminary studies on the position of invasive gobies in the food web in the Baltic Sea have suffered from the inability to discriminate half-digested Ponto-Caspian gobies, particularly at young stages, from native gobies, when analyzing gut contents (personal communications of participants of the Nordic Goby Meeting 2015, Umea). We propose that our primers may be useful for molecular analyses of predator’s guts for the presence of invasive Ponto-Caspian gobies.

## Supporting Information

S1 Fig Primer performance. For each species, one primer pair outperforms the other pairs. “Goby”, primers designed to detect all five invasive Ponto-Caspian goby species (Ponto-Caspian goby primers). “NM”, primers designed to detect *Neogobius melanostomus*. “PK”, primers designed to detect *Ponticola kessleri*. Template: 5ng of *Neogobius melanostomus* DNA (goby, NM) or *Ponticola kessleri* DNA (PK).
(EPS)

S1 Supplementary Material Sequences and alignment used to design specific primers. (DOCX)

S2 Supplementary Material Technical instructions on building the water sampler. (PDF)

S3 Supplementary Material Extraction protocol test methods. (DOCX)

S4 Supplementary Material Detailed step-by-step protocol describing every step from sampling to detection. (DOCX)

S1 Table Review of methods. For 60 publications, every step from field sampling to amplification and detection was analyzed and is presented in a tabular form.
(CSV)
