# Supplementary material for: An eDNA Assay to Monitor a Globally Invasive Fish Species from Flowing Freshwater
Source: PLoS One. 2016 Jan 27;11(1):e0147558. doi: 10.1371/journal.pone.0147558 (PMC4729461; doi:10.1371/journal.pone.0147558)
Supplement: S1 Supplementary Material — (DOCX) [file pone.0147558.s002.docx]

>NM_gi|671723846|gb|KF549990.1ATCGCAAACCACGCACTAGTAGACCTACCCGCCCCCTCTAACATTTCTGCCTGATGAAACTTCGGCTCTCTCCTAGGCCTATGTTTAATTGCCCAAATTGTAACAGGGCTTTTTCTAGCCATACACTACACCTCGGATATCGCCACAGCCTTCTCGTCAGTAACCCACATCTGCCGAGATGTTAACTTTGGCTGACTAATCCGCAATATGCACGCCAACGGAGCTTCCTTTTTCTTTATTTGCATCTACCTTCACATTGGTCGAGGCCTCTATTACGGCTCATACCTATACAAAGAGACCTGAAACATCGGAGTAGTACTACTACTCCTAGTTATGATAACTGCTTTTGTGGGCTACGTCCTCCCATGAGGACAAATGTCCTTTTGAGGGGCCACCGTGATCACCAACCTCCTCTCTGCCGTCCCTTACGTGGGCGGAAACCTAGTACAATGAATTTGGGGGGGATTTTCAGTCGATAACGCAACCCTAACCCGATTTTTCGCCTTCCATTTTATTCTGCCCTTTATTATTCTAGCTGCAACGTTACTCCACTTACTATTCCTACACGAAACGGGATCCAATAATCCAGTCGGCCTTAATTCAGATGCCGACAAAATTCCTTTTCACCCTTACTTTACCTATAAAGACCTACTAGGGTTTGCCATCATAATATTTGCTTTAACCTCTCTCGCCCTTTTTTCCCCAAACTTCCTCGGAGACCCAGACAACTTCATCCCAGCAAACCCCCTCGTCACTCCCCCCCACATCAAACCTGAATGGTACTTCTTATTTGCTTACGCCATCCTCCGCTCTATCCCTAACAAACTTGGGGGAGTCCTAGCACTGCTCGCCTCCATCTTAGTTTTAATACTAGTCCCCCTACTCCACACCTCTAAGCACCGGAGCCTCACCTTCCGCCCACTCTCACAATTCTTATTTTGAACACTAGTGGCAGACGTAATAATCCTAACCTGAATTGGCGGCATACCAGTAGAAGACCCCTATGTGATGATCGGACAGCTAGCCTCAATATTGTACTTTTCCATCTTTCTCGGCTTATTCCCCATAGTGAGCTGACTAGAGAACAAACTTTTATTA

>NM_gi|168485493|gb|EU331236.1ATGGCCAGCCTACGAAAAACACACCCGCTGCTTAAAATCGCAAACCACGCACTAGTAGACCTACCCGCCCCCTCTAACATTTCTGCCTGATGAAACTTCGGCTCTCTCCTCGGCCTATGTTTAATTGCTCAAATTGTAACAGGGCTTTTTCTAGCCATACACTACACCTCGGATATCGCCACAGCCTTCTCATCAGTAACTCACATCTGCCGAGATGTTAACTTTGGCTGACTAATCCGCAATATGCACGCCAACGGAGCTTCCTTTTTCTTTATTTGCATCTACCTTCACATTGGTCGAGGCCTCTATTACGGCTCATACCTATACAAAGAGACCTGAAACATCGGAGTAGTACTACTACTCCTAGTTATAATAACTGCTTTTGTGGGCTACGTCCTCCCATGAGGACAAATGTCCTTTTGAGGGGCTACCGTGATCACCAACCTCCTCTCTGCCGTCCCTTACGTGGGCGGAAACCTAGTACAATGAATTTGGGGTGGATTTTCAGTCGATAACGCAACCCTGACCCGATTTTTCGCCTTCCATTTTATTCTGCCCTTTATTATTCTAGCTGCAACATTACTCCACTTATTATTCCTACACGAAACGGGATCCAATAATCCAGTCGGCCTTAATTCAGATGCCGACAAAATTCCTTTTCACCCTTACTTTACCTATAAAGACCTACTAGGGTTTGCCATCATAATATTTGCTTTAACCTCTCTCGCCCTTTTTTCCCCAAACTTTCTCGGAGACCCAGACAACTTCATCCCAGCAAACCCCCTCGTCACTCCCCCCCACATCAAACCTGAATGATACTTCTTATTTGCTTACGCCATCCTCCGCTCTATCCCTAACAAACTTGGAGGAGTCCTAGCACTGCTCGCCTCCATCCTAGTTTTAATACTAGTCCCCTTACTCCACACCTCTAAGCACCGGAGCCTTACCTTCCGCCCACTCTCACAATTCTTATTTTGAACACTAGTGGCAGACGTAATAATCCTAACCTGAATTGGCGGCATACCAGTAGAAGACCCCTATGTAATAATCGGACAGCTAGCCTCAATATTGTACTTTTCCATCTTTCTCGGCTTATTCCCCATAGCGAGCTGACTAGAGAACAAACTTTTATTAACATAACTGCACTAGTAGCTCAGCGCCAGAGCGCCGGCCTTGTAAGCCGGACGCCGGAGGTTAAACCCCT

>PK_gi|589911366|gb|KC886260.1CCCCTGCTTAAAATCGCAAACCATGCACTGGTAGACTTACCTGCCCCCTCAAACATTTCTGCCTGATGGAACTTTGGCTCTCTACTAGGCCTATGCCTGATTGCTCAAATTGTCACAGGACTATTTTTAGCTATACATTATACCTCTGATACTGCCACAGCCTTTTCATCTGTGGCACACATCTGCCGAGATGTAAATTTCGGTTGACTCATCCGAAACATTCATGCCAATGGTGCCTCTTTCTTCTTTATCTGTATTTACATGCATATTGGACGAGGACTATATTACGGCTCCCATCAATATAAAGAAACTTGAAATATTGGAGTCGTACTTCTGCTCCTAGTGATAATAACCGCCTTCGTGGGCTACGTCCTGCCATGAGGACAAATATCCTTCTGAGGTGCCACCGTAATTACAAACCTTCTCTCTGCTGTTCCCTACATTGGAGGAGACTTAGTACAATGAATTTGAGGGGGCTTCTCAGTTGATAACGCAACCCTAACACGATTTTTTGCCTTTCACTTCCTTCTACCATTTGTCATTTTGGCTGCCACTCTAATTCACCTACTTTTTCTACACGAAACAGGCTCTAATAACCCTATTGGGTTGAACTCAGACGCCGACAAAATCCCCTTCCACCCCTACTTTATTTATAAAGACCTGCTAGGTTTTGCTATTATATTACTTGCCCTAACCTCCTTAGCATTATTTATCCCCAACTACCTTGGAGACCCCGACAACTTTATCCCAGCAAACCCACTAGTTACCCCACCCCACATTAAGCCTGAGTGGTACTTCCTGTTCGCCTATGCCATTTTACGCTCCATCCCTGACAAGTTAGGGGGAGTCATAGCACTACTCGCCTCCATTTTGATTCTAATACTGGTACCATTTCTCCACACCTCTAAACACCGGAGCCTCACCTTTCGTCCATTTTCTCAACTACTGTTCTGAATACTAGTAGCAGATGTAGCAATCCTCACCTGAATTGGAGGAATGCCAGTAGAACACCCCTATATCATCATCGGTCAAATTGCTTCAGTGCTATACTTCTCAATTTTTCTCGTCCTATTCCCTATTGTAGGCTGACTAGAAAATAAATTTTTATTAA

>NF_gi|257220486|gb|GQ444442.1ATGGCCAGCCTACGAAAAACACACCCACTGCTTAAAATTGCAAACCATGCACTAGTAGACCTACCCGCCCCCTCTAATATTTCGGCCTGATGAAATTTCGGCTCCCTCCTCGGCCTATGCTTAATTGCCCAAATTGTTACAGGGCTTTTCTTAGCCATACACTACACCTCTGATATTGCCACAGCCTTCTCATCAGTAGCACACATCTGCCGAGATGTAAACTTTGGCTGACTAATCCGCAATATGCACGCCAACGGGGCCTCATTCTTCTTTATCTGCATCTACCTTCACATTGGACGAGGCCTCTACTACGGCTCATACCTGTATAAAGAGACTTGAAATATTGGGGTTATTCTTTTACTCCTGGTTATAATAACTGCTTTTGTAGGCTACGTCCTCCCATGAGGACAAATATCCTTTTGAGGTGCCACTGTAATTACTAACCTTTTATCTGCTGTCCCCTACGTAGGAGGGAGCCTAGTACAATGGATCTGAGGCGGGTTTTCAGTTGACAACGCAACCCTGACACGATTTTTTGCCTTCCACTTCCTACTACCCTTCGTTATTCTAGCCGCCACGCTCCTCCACCTACTATTTCTCCACGAAACGGGCTCCAACAACCCAGTAGGTCTTAACTCAGATGCCGACAAAATTCCTTTTCACCCCTACTTTACCTATAAAGATTTGTTAGGGTTTGCCATCATATTACTAGCCCTAACCTCTCTTGCCCTCTTTACCCCAAATTACCTAGGAGATCCCGACAACTTCATCCCAGCAAACCCCCTCGTTACTCCGCCCCACATTAAACCTGAGTGATACTTCTTATTTGCTTACGCCATCCTACGCTCCATCCCCAATAAACTTGGAGGAGTCCTAGCATTACTTGCCTCTATTTTAGTTCTGATACTAGTACCTTTCCTCCACACCTCTAAGCACCGAAGCCTCACCTTCCGCCCACTTTCACAATTCCTATTTTGGATACTAGTGGCAGACGTAATGATCCTAACCTGAATTGGTGGCATACCAGTTGAGGATCCATATGTTTTAATCGGACAAGTCGCCTCTGTATTATACTTCTCCATCTTTCTCGGCCTATTCCCCATAGCGGGCTGGCTAGAGAACAAACTTTTATTAACATAG

>NF_gi|671723852|gb|KF549993.1ATTGCAAACCACGCACTAGTAGACCTACCCGCCCCCTCCAACATTTCAGCCTGATGAAACTTCGGCTCCCTCCTCGGCCTATGCTTAATTGCCCAAATTGTGACGGGACTTTTTTTAGCTATACACTATACATCTGATATTGCCACAGCCTTCTCATCAGTAGCACACATCTGCCGAGATGTAAACTTTGGATGGCTAATCCGCAACATGCACGCCAACGGGGCTTCCTTCTTCTTTATCTGCATTTACCTTCACATTGGACGAGGCCTCTATTACGGCTCATACCTGTATAAAGAAACCTGAAATATCGGTGTAATTCTTCTCCTCCTGGTTATAATGACTGCTTTTGTAGGTTACGTCCTCCCATGGGGACAAATATCCTTTTGAGGTGCCACCGTAATTACTAATCTTCTATCTGCCGTCCCTTACGTAGGAGGAAGCCTAGTACAATGAATCTGAGGGGGGTTTTCGGTTGATAACGCAACCCTAACACGATTTTTCGCCTTCCACTTCCTATTGCCCTTTGTAATCCTAGCCGCCACGCTCCTCCACCTGCTATTTCTCCACGAAACGGGCTCCAACAATCCAGTGGGGCTTAACTCAGACGCCGACAAAATTCCTTTCCACCCCTACTTTACCTATAAAGATTTATTAGGATTTGCCATCATATTACTTGCTCTTGCCTCTCTTGCCCTCTTTACCCCAAACTACCTAGGAGACCCGGACAATTTCATCCCAGCAAATCCCCTTGTCACCCCTCCCCACATTAAGCCTGAGTGGTACTTCTTATTTGCTTACGCCATTCTCCGCTCCATCCCCAATAAACTTGGGGGAGTACTAGCATTGCTTGCCTCCATCTTAGTTTTGATGCTTGTGCCTTTTCTCCACACCTCTAAACACCGAAGCCTCACCTTCCGCCCACTCTCACAATTCCTATTCTGAACACTAGTAGCAGACGTAGTCATCCTAACCTGAATTGGCGGCATGCCAGTCGAAGACCCATATGTTTTAATCGGACAACTAGCCTCTGTTTTATACTTCTCCATCTTTCTCGTATTCTTCCCAATAGCCGGCTGACTAGAGAACAAACTTTTATTA

>BG_gi|526850801|gb|KF415509.1ATGGCCAGCCTACGAAAAACACACCCCCTACTAAAAATTGCTAACCATGCACTGGTAGACTTACCTGCCCCCTCGAACATTTCTGCCTGATGGAACTTTGGTTCACTACTGGGCTTATGCCTTATTGCTCAAATTATCACAGGACTATTTCTAGCTATACATTATACCTCTGATATTGCCACAGCCTTCTCATCCGTAGCACATATCTGCCGAGATGTAAATTTTGGATGACTTATCCGAAATATTCACGCCAACGGGGCCTCCTTCTTCTTTATCTGTATTTATATACACATTGGACGAGGCCTTTATTATGGATCCCACCAATACAAAGAAACCTGAAATATTGGAGTCGTCCTACTGCTCCTAGTAATAATAACCGCCTTCGTGGGTTACGTCTTACCATGAGGGCAAATATCTTTCTGAGGTGCAACCGTAATTACAAATCTTCTCTCTGCTGTTCCCTACATCGGGGGAGACTTAGTGCAATGAATTTGAGGGGGCTTCTCAGTCGACAACGCAACCCTAACACGATTTTTTGCCTTTCACTTCCTTTTCCCCTTTGTGATTTTAGCCGCCACCCTAATTCACCTTCTCTTTTTACACGAAACAGGATCTAATAACCCTCTTGGCCTTAACTCAAACGCCGACAAAATCCCCTTTCACCCCTATTTTATCTATAAAGACCTGCTAGGTTTTGTTATTATATTATTTGCCCTAACCTCCCTAGCACTATTTTCCCCTAATTATCTCGGAGACCCCGACAACTTTATTCCAGCAAATCCCCTAGTTACCCCGCCCCACATCAAGCCTGAATGATACTTCCTATTTGCTTACGCCATTCTACGCTCCATCCCTGATAAATTGGGAGGAGTAATAGCACTACTCGCCTCCATCCTAATTTTAATACTAGTGCCATTTCTTCATACCTCCAAACATCGAAGCCTCACCTTTCGTCCACTCTCTCAACTACTATTCTGAACGCTAGTAGCAGATGTAGCAATCCTTACCTGAATTGGGGGAATACCAGTAGAACACCCCTATATTATTATTGGTCAAATTGCTTCAGTGCTATACTTTTCCATTTTTCTCGTCCTATTTCCTATTGTAGGCTGACTAGAAAATAAATTTTTATTAAACT

>BG_gi|188988554|gb|EU444667.1ATGGCCAGCGTACGAAAAACACACCCCCTACTAAAAATTGCTAACCATGCACTGGTAGACTTACCTGCCCCCTCGAACATTTCTGCCTGATGGAACTTTGGTTCACTACTGGGCTTATGCCTTATTGCTCAAATTATCACAGGACTATTTCTAGCTATACATTATACCTCTGATATTGCCACAGCCTTCTCATCCGTAGCACATATCTGCCGAGATGTAAATTTTGGATGACTTATCCGAAATATTCACGCCAACGGGGCCTCCTTCTTCTTTATCTGTATTTATATACACATTGGACGAGGCCTTTATTATGGATCCCACCAATACAAAGAAACCTGAAATATTGGAGTCGTCCTACTGCTCCTAGTAATAATAACCGCCTTCGTGGGCTACGTCTTACCATGAGGGCAAATATCTTTCTGAGGTGCAACCGTAATTACAAACCTTCTCTCTGCTGTTCCCTACATCGGGGGAAACTTAGTGCAATGAATTTGAGGGGGCTTCTCAGTCGACAACGCAACCCTAACACGATTTTTTGCCTTTCACTTCCTTTTCCCCTTTGTGATTTTAGCCGCCACCCTAATTCACCTTCTCTTTTTACACGAAACAGGATCTAATAACCCTCTTGGCCTTAACTCAAACGCCGACAAAATCCCCTTTCACCCCTATTTTATCTATAAAGACCTGCTAGGTTTTGTTATTATATTATTTGCCCTAACCTCCCTAGCACTATTTTCCCCTAATTATCTCGGAGACCCCGACAACTTTATTCCAGCAAATCCCCTAGTTACCCCGCCCCACATCAAGCCTGAATGATACTTCCTATTTGCTTACGCCATTCTACGCTCCATCCCTGATAAATTGGGAGGAGTAATAGCACTACTCGCCTCCATCCTAATTTTAATACTAGTGCCATTTCTTCATACCTCCAAACATCGAAGCCTCACCTTTCGTCCACTCTCTCAACTACTATTCTGAACGCTAGTAGCAGATGTAGCAATCCTTACCTGAATCGGGGGAATACCAGTAGAACACCCCTATATTATTATTGGTCAAATTGCTTCAGTGCTATACTTTTCCATTTTTCTCGTCCTATTTCCTATTGTAGGCTGACTAGAAAATAAATTTTTATTAAACT

>PM_gi|526851063|gb|KF415640.1ATGGCCAGCCTACGAAAAACGCATCCCCTACTTAAAATTGCAAACCATGCACTAGTGGATTTACCTGCCCCTTCAAACATTTCTGCCTGATGAAACTTCGGCTCTCTTCTGGGCTTATGTCTTATTGCTCAAATTATTACAGGACTATTTTTAGCTATACACTATACCTCTGATATCGCCACAGCCTTTTCATCCGTGGCACACATCTGCCGAGATGTTAATTTTGGATGACTTATCCGAAATCTTCACGCCAACGGGGCCTCTTTTTTCTTCATTTGTTTGTACCTGCACATCGGACGGGGACTCTACTACGGCTCATACTTATTTAAAGAAACATGAAATATTGGAGTAGTCCTATTTCTCCTAGTAATAATAACCGCCTTCGTGGGCTATGTACTTCCATGAGGACAAATATCTTTCTGAGGAGCAACCGTAATTACAAATCTCCTCTCTGCTATCCCTTACATTGGCGGGGATTTAGTACAATGAATCTGGGGAGGCTTTTCAGTTGATAATGCAACCCTAACACGATTTTTTGCCTTTCACTTTCTGCTACCCTTCGTTATCTTGGCCGTCACCCTAGTTCACCTACTCTTTCTACACCAAACAGGATGCAATAACCCACTGGGCCTTAACTCTAATGCAGATAAAATCCCATTCCACCCCTACTTCACTTACAAAGACTTACTAGGGTTTGCCCTCATACTAATTTCCCTAACCTGTCTGGCACTATTTTTACCTAACTATCTAGGAGACCCTGACAACTTCATCCCCGCAAACCCCTTAATAACTCCCCCCCATATCAAACCCGAGTGGTACTTCTTATTTGCCTACGCCATCTTACGCTCCATTCCCAACAAACTTGGGGGAGTTATAGCACTACTAGCCTCTATCTTAATCTTAATACTAGTGCCCTTTCTTCATACCTCTAAACATCGCAGCCTCACCTTTCGCCCATTTTCCCAACTATTATTCTGAACACTAGTTGCAGACGTAGCAATCCTTACCTGAATTGGAGGCATACCAGTAGAACATCCTTATATTATTATTGGGCAAATTGCCTCAGTACTATACTTTTCCCTCTTCCTAGTCCTATTTCCTATTGTAGGATGACTAGAAAACAAACTCTTACTGAACT

>PM_gi|188988552|gb|EU444666.1ATGGCCAGCCTACGAAAAACACATCCCCTGCTTAAAATTGCAAACCACGCACTAGTGGACCTACCTGCCCCCTCAAATATTTCCGCCTGATGAAACTTCGGCTCTCTCCTTGGCTTATGCTTAATTGCTCAAATTGTTACAGGACTGTTTTTAGCTATACACTACACCTCTGATATCGCCACAGCCTTTTCATCTGTAGCACATATTTGTCGAGATGTAAACTTTGGATGACTTATTCGAAACCTTCACGCCAATGGAGCCTCCTTCTTTTTCATTTGTTTGTACCTTCACATTGGGCGAGGCCTCTACTACGGCTCATACTTATATAAAGAGACATGATTTATTGGGGTAGTACTATTCCTCCTAGTTATAATAACCGCATTCGTGGGCTATGTTCTTCCATGGGGGCAAATATCCTTCTGAGGGGCAACCGTAATTACAAACCTCCTCTCTGCTATCCCTTATGTAGGAACTGATCTAGTACAGTGAATTTGAGGGGGCTTCTCAGTTGATAACGCAACCCTCACACGATTCTTCGCCTTTCATTTCCTACTGCCATTTGTTGTCTTGGCCATCACCTTAATTCACCTACTTTTTCTACACGAAACAGGGTCCAATAACCCCCTTGGCCTTAACTCAAACGCAGACAAAATCCCATTTCACCCCTACTTCATCTATAAAGACTTATTAGGATTTGCCATCATATTAATTTCCCTAACCTGCTTAGCACTGTTTTTACCTAACTACCTTGGGGACCCTGACAACTTCATTCCCGCAAATCCCTTAATAACCCCACCTCATATTAAGCCCGAATGATATTTCTTATTTGCCTACGCCATCTTGCGCTCTATTCCTAATAAACTTGGGGGAGTAATAGCACTGCTCGCTTCTATTTTAATCTTATTATTAGTGCCCTTTCTTCACACCTCTAAACATCGAGGCCTCACCTTTCGCCCGTTTTCCCAAATACTATTCTGAACACTAGTAGCAGATGTGGCAATCCTTACCTGAATTGGGGGGATGCCAGTAGAACACCCCTATATTATTATCGGACAAATTGCCTCAGCACTTTACTTTTCTATTTTTCTTTTCCTATTTCCCGTTGTAGGATGACTAGAAAATAAACTTTTATTAAACT

>G_niger_gi|526850949|gb|KF415583.1|ATGGCCCCCCTACGAAAGACTCACCCCCTGCTTAAAATCGCAAACCACGCACTGGTTGACCTCCCCGCCCCCTCTAACATTTCTGCCTGATGGAACTTCGGCTCCCTCCTCGGGCTCTGCCTTATTGCCCAAATCCTCACTGGCCTTTTCTTAGCCATACATTACACCGCAGACATTACAATGGCCTTCTCCTCCGTAGCGCACATCTGCCGAGACGTCAACTTCGGGTGGCTTATCCGGAATATGCATGCCAACGGCGCCTCATTCTTTTTTATCTGCATTTACCTTCACATCGGACGAGGCCTCTACTACGGGTCCTACCTTTATAAGGAAACCTGGACTATTGGAGTGGTACTGCTCCTTCTTGTCATGATGACCGCCTTCGTCGGGTACGTCCTCCCCTGAGGACAGATATCTTTCTGGGGTGCAACCGTGATTACGAACCTACTTTCCGCCGTGCCCTACGTCGGAGGAACCCTTGTGCAATGAATCTGGGGCGGCTTCTCAGTCGACAACGCAACGCTAACTCGATTCTTTGCCTTCCACTTCCTCTTTCCATTCGTTATCCTTGCCGTTACCCTCCTTCACCTTCTCTTTCTCCACGAGACCGGCTCCAACAACCCCACCGGGCTTAATTCTGACGCGGACAAGGTTCCCTTTCACCCTTACTTCTCCTACAAGGACCTCCTTGGATTCGCCATCATACTCTTCGCCCTCACCTCCTTGGCTCTTTTTACCCCCAACTACCTAGGGGATCCGGACAACTTCATTCCTGCCAACCCCCTTGTTACCCCGCCTCACATTAAACCAGAGTGATACTTTCTTTTTGCCTACGCAATCTTGCGCTCCATTCCTAACAAACTTGGAGGAGTCCTTGCCCTTCTGGCCTCTATTTTGGTCCTTTTAGTCGTCCCCTTCCTTCACACTTCTAAGCAGCGGGGGCTCACCTTCCGTCCTCTTTCCCAGCTTTTATTCTGAGTCTTAGTGGCCGACGTCATCATCCTCACCTGAATCGGCGGCATACCCGTCGAGCACCCCTATGTTATCATTGGACAAATCGCATCCGTATTGTACTTCTCCATTTTTCTCCTCCTTTTCCCCACAGCTGGGTGGCTAGAGAACAAGATTCTATTCAGCGCCT

>P_minutus_gi|237638806|gb|FJ526776.1|ATGACCAGCCTACGGAAAACCCACCCGCTCCTTAAGATTGCAAATGGTGCACTTGTAGACCTCCCTGCACCCTCAAATATCTCTGCCTGATGGAATTTTGGCTCTTTACTGGGTATCTGCCTTATTGCTCAAATTCTGACTGGCCTCTTTCTGGCGATACACTACACATCTGATATCGCCACTGCTTTTTCATCTGTCGCCCACATTTGCCGTGATGTCAACTTTGGCTGACTGATCCGGAACATGCACGCCAACGGTGCCTCCTTCTTCTTCATCTGCCTTTACCTCCATGTGGGGCGGGGCCTCTACTACGGCTCCTACCTCTATAAGGAAACGTGAAATATCGGCGTCGTTCTGCTTCTCCTGGTAATGATGACTGCCTTCGTCGGCTACGTCCTCCCCTGGGGACAGATGTCGTTTTGGGGGGCAACTGTCATTACCAACCTCCTCTCCGCGGTCCCCTACGTGGGCAATAGCCTTGTGCAGTGAATCTGGGGGGGCTTCTCGGTTGACAATGCTACCCTCACGCGCTTCTTTGCCTTCCACTTTCTGTTCCCCTTTGTAGTATTAGCAGCTACTATTCTCCACTTGCTATTCCTTCACGAGTCGGGGTCGAACAACCCCACGGGCCTGAACTCCGACGCCGATAAGGTTCCCTTCCACCCATACTTCTCCTACAAGGATCTTATCGGTTTCGCCGTAATGCTAGTTGCGCTCACCTCACTCGCCCTGTTCTCCCCCAACTACCTTGGCGACCCAGACAACTTCACCCCCGCCAATCCTCTGGTTACCCCACCCCACATCAAGCCTGAGTGGTACTTCTTGTTTGCCTACGCCATCCTGCGATCTATCCCTAACAAGCTTGGAGGAGTCCTCGCCCTACTAGCCTCCATTCTTGTACTACTCCTAGTACCATTCCTCCACACCTCGAAGCAACGAGGGCTTACCTTCCGCCCCCTTTCGCAGTTCTTGTTCTGAACACTCGTGGCTGATGTGCTCATTCTAACCTGAATCGGTGGCATGCCGGTCGAGCACCCCTACATCATCATCGGACAGCTCGCGTCCCTCCTCTACTTCTCCATCTTCCTCGCCCTCTTTCCCCTCGCCGGGCTCGCGGAGAACAAACTTCTTAAACTAGCCT

>CG1_gi|27762429|gb|AY116366.1ATGGNCANCCTACGAAAAACCCACCCCTTACTAAAAATCGCAAACAATGCACTAGTTGACCTTCCAGCCCCCTCAAATATTTCGGTATGATGAAACTTTGGTTCCCTCCTCGGTCTTTGCTTAATTATCCAAATCCTAACCGGGCTCTTCCTCGCCATACACTACACCTCTGACATCGCAACAGCCTTCTCATCAGTCGGCCACATCTGCCGAGATGTCAACTACGGATGACTTATCCGTAACCTCCACGCCAACGGTGCCTCTTTCTTCTTTATTTGCATCTATATGCACATCGGACGGGGCCTTTACTACGGCTCCTACCTCTATAAAGAAACTTGAAACATTGGGGTCGTCCTCCTGCTCCTTGTAATAATAACCGCTTTCGTAGGGTACGTCCTCCCCTGAGGACAGATGTCATTCTGAGGGGCCACTGTCATCACAAACCTTCTTTCTGCAGTTCCTTACATCGGCAACGCCCTGGTCCAATGAATCTGAGGAGGCTTCTCAGTAGACAACGCCACACTCACACGATTCTTTGCCTTTCACTTCCTCTTCCCCTTCGTAATTGCAGGTGCAACCCTCATCCATCTGCTCTTCCTACATGAAACTGGCTCAAACAACCCCCTTGGTTTAAACTCAGATGCAGACAAAATCTCTTTCCACCCTTACTTTTCTTACAAAGACCTGTTAGGCTTTGCAGCACTCTTAATTGCCCTCACAGCCCTAGCACTCTTCTCCCCTAACCTCTTAGGAGACCCAGATAACTTTACTCCTGCTAACCCCCTGGTAACCCCTCCCCACATCAAGCCTGAATGATACTTCTTGTTTGCCTACGCCATCCTTCGCTCTATCCCCAACAAGCTTGGAGGCGTCCTAGCCCTCCTGGCATCCATCCTGGTACTCATAGTAGTACCCATCCTCCACACATCAAAACAACGAGGCCTAACCTTTCGCCCCATGACCCAATTTCTATTTTGAACACTCATCGCAGACGTCGCCATTCTCACATGAATTGGAGGCATGCCCGTCGAACACCCCTTTATTATCATTGGCCAAATTGCATCTCTCCTATACTTCTTTCTTTTCTTAGCCTTATTCCCCCTAGCAGGCTGAATAGAAAACAAAGCCTTAGGATGAACTT

>GA1_gi|25140372|gb|AY116004.1ATGGCAAGCCTACGAAAAACGCACCCCCTACTAAAAATCGCTAACAATGCACTAGTCGACCTCCCCGCCCCCTCAAATATTTCAGTATGATGAAACTTTGGTTCCCTCCTTGGACTTTGCTTAATTATCCAAATTCTCACTGGGCTTTTCCTTGCAATACACTACACTTCCGATATTGCTACAGCTTTTTCCTCCGTCGGACACATCTGCCGAGATGTAAATTACGGCTGACTAATTCGAAACCTCCATGCCAATGGCGCATCCTTTTTCTTCATCTGTATCTATATGCATATCGGCCGAGGACTTTACTATGGCTCTTACCTATACAAAGAAACCTGAAACATCGGAGTAATTCTTCTACTTTTAGTCATGATAACAGCCTTCGTTGGTTATGTCCTTCCATGAGGACAAATATCTTTCTGAGGAGCTACAGTTATTACCAACCTACTTTCAGCCGTCCCATACGTTGGCAACTCATTAGTTCAATGAATTTGAGGGGGCTTTTCCGTTGACAACGCCACCTTAACACGTTTCTTTGCCTTTCACTTCTTATTCCCATTTGTCATTGCTGGTGCCACACTTGTTCACCTTCTTTTCCTTCACCAGACAGGCTCTAATAACCCTCTTGGCCTGAACTCAGACGCTGACAAAATCTCCTTTCACCCCTACTTTTCTTATAAAGACCTCCTTGGCTTCGCAGCCCTACTAATTGCCCTTACATCCCTAGCACTATTTGCTTCTAACCTGCTTGGAGATCCCGATAACTTCACCCCTGCAAACCCATTAGTCACTCCACCTCACATCAAGCCTGAATGGTACTTCTTGTTTGCCTACGCCATTCTTCGATCTATCCCAAATAAACTTGGAGGCGTCCTTGCACTCCTTGCCTCCATCCTAGTCTTGATAGTAGTCCCCATCCTTCATACATCTAAACAACGTGGCCTTACATTTCGACCCCTTACGCAATTTTTATTTTGAACTCTTATTGCAGATGTAGCCATTCTTACCTGAATTGGCGGTATACCCGTTGAACACCCCTTCATCATTATTGGACAAGTTGCATCCGTACTTTACTTCTCTTTATTCCTAGTTTTATACCCAGGAGCAGCAGTAATAGAGAACAAAATGCTTGAATGAACA

>GC3_gi|20339610|gb|AF386598.1ATGGCAAGCCTCCGAAAAACTCACCCCCTACTAAAAATTGCAAACGGCGCACTAGTTGACCTACCCGCCCCCTCTAATATTTCAGTATGATGAAACTTTGGTTCCTTACTTGGCCTCTGTTTAATCACCCAAATCCTTACAGGCCTCTTTTTAGCAATACACTATACCGCAGACATCGCCACAGCCTTTTCATCAGTAGCCCATATCTGCCGAGATGTAAATTATGGATGACTTATTCGAAACCTTCATGCCAACGGAGCATCATTCTTTTTCATTTGCATTTATATACACATTGGCCGAGGCCTATACTACGGATCCTACCTTTATAAAGAAACATGAAACATCGGAGTTGTCCTATTGCTCCTAGTAATAATGACCGCCTTTGTAGGATACGTTTTACCCTGAGGACAGATATCTTTCTGAGGAGCAACTGTCATCACCAACCTCCTGTCCGCAGTACCTTATATTGGCAACACCCTAGTACAATGAATCTGGGGAGGCTTCTCAGTTGATAATGCCACCCTCACCCGATTCTTTGCTTTCCATTTCCTCTTCCCCTTTGTCATTGCCGGCGCTACCCTTATTCACCTCCTCTTTCTACACGAAACAGGCTCGAACAACCCCTTAGGTTTAAACTCTGACGCCGACAAAGTCTCTTTCCACCCCTATTTCTCATATAAAGACCTCTTAGGCTTTGCAGTCCTCCTTATTACCTTAACATCTCTAGCCCTTTTCTCCCCTAACCTCTTAGGAGACCCAGATAATTTTACCCCTGCAAACCCCCTAGTTACACCACCTCACATCAAACCTGAGTGATACTTCCTATTTGCATACGCAATCCTTCGCTCCATCCCCAACAAGCTAGGAGGAGTTTTAGCCCTACTAGCCTCAATTCTTATCCTTATGGCTGTCCCAATTCTTCACACCTCCAAACAACGAGGCATCACTTTCCGACCGCTCTCACAGTTCCTCTTTTGAACGCTAATTGCAGACGTTGCTATCCTCACCTGAATTGGAGGAATACCAGTCGAACACCCTTTCATCATCATTGGCCAAATCGCATCTTTCCTATATTTCTTCCTATTCCTCGTTCTCGCCCCACTAGCAGGATGAGTCGAAAACAAGGCCCTCGGATGATTC

>PF3_gi|164562184|gb|EU348846.1ATGGCAAGCCTCCGAAAAACTCACCCTCAACTAAAAATTGCAAACAACGCACTACTTGACTTACCCGCCCCTTCTAATATTTCAGTATGATGAAATTTTGGTTCCCTACTTGGCCTCTGTTTAATTACCCAAATCCTAACTGGCCTTTTTCTGGCAATACACTATACCGCAGACATCGCAACAGCCTTTTCATCAGTCGCCCACATTTGCCGAGACGTAAACTACGGCTGACTCATTCGAAATATTCATGCCAATGGTGCATCCTTCTTCTTTATTTGCATCTATATGCACATCGGCCGGGGTTTATATTACGGCTCCTATCTTTACAAAGAAACATGAAACATTGGAGTTGTTCTCCTTCTCCTAGTAATAATAACCGCCTTTGTTGGGTACGTCCTGCCCTGAGGACAAATATCTTTCTGGGGTGCGACCGTCATTACCAACCTTCTGTCAGCAGTCCCTTACATTGGCAATACCCTTGTTCAATGGATCTGAGGCGGCTTCTCAGTAGATAACGCCACCCTCACTCGATTCTTTGCCTTCCACTTCCTATTCCCCTTCGTCATTGCAGGTGCCACCCTCATCCACCTGCTTTTCCTTCATGAAACAGGCTCAAACAATCCCTTAGGTTTAAACTCTGACGCAGATAAAGTTTCCTTTCATCCTTATTTTTCTTACAAAGACCTCTTAGGATTTGCAGTACTACTAATCGCTCTAACAGCTCTAGCTCTCTTCTCCCCTAATTTATTAGGGGATCCTGACAACTTTACCCCAGCCAACCCCTTAGTTACCCCACCACACATCAAGCCTGAATGATACTTCTTGTTTGCCTACGCCATCTTACGCTCGATTCCAAACAAACTAGGAGGGGTGTTAGCCTTACTTGCCTCCATCCTGGTTCTTATAGTTGTCCCCATCCTTCACACTTCTAAACAACGTGGCATTACATTCCGACCGCTCTCCCAATTCCTCTTTTGAACATTAATCGCAGATGTTGTTATCCTTACCTGAATTGGAGGAATACCTGTAGAACACCCCTTCATCATTATTGGCCAAGTCGCATCTTTCTTGTACTTCTCCCTTTTCCTCGTCCTCACGCCACTAGCAGGATGGGCCGAAAACAAAGCCCTCGGA

>A_anguilla1_gi|159461951|gb|EU223997.1ATGGCAAACCTACGAAAAACCCACCCACTTCTAAAAATTGCTAACGATGCCCTAGTGGATCTACCAACCCCATCCAATATTTCAGCATGATGAAATTTTGGCTCTCTTCTAGGATTATGTCTTATTTCACAAATCCTTACAGGACTATTCCTAGCCATACATTATACATCAGACATCTCAACTGCCTTCTCCTCAGTAGCTCACATCTGCCGAGACGTTAACTATGGATGACTAATTCGCAACCTACATGCAAATGGAGCCTCATTCTTCTTTATCTGCCTATACCTCCACATTGCCCGAGGACTTTACTACGGCTCATACCTTTACATAGAAACATGAAACATTGGAGTTGTATTATTCCTATTAGTAATAATAACAGCATTCGTAGGATATGTGCTTCCATGAGGACAGATATCATTCTGAGGTGCTACAGTAATTACCAACCTACTATCTGCCGTCCCATATGTGGGGAACTCCCTAGTCCAATGAATCTGAGGGGGATTCTCAGTTGACAACGCCACATTAACCCGATTCTTCGCATTCCACTTCCTATTTCCATTTGTAGTTGCTGGAGCCACAATACTTCACCTCCTATTCCTCCATGAAACAGGATCAAACAATCCGGTAGGATTAAACTCCGACGCAGACAAAATCCCATTCCACCCATACTTCTCCTACAAAGATCTACTGGGGTTCATTATCATGCTCACCGCCCTAACAATACTTGCCCTATTCTACCCGAACCTGCTTGGAGACCCAGACAACTTCACCCCGGCAAATCCAATAGTTACTCCGCCACACATTAAGCCAGAGTGGTATTTTCTATTTGCCTACGCCATTCTACGATCAATTCCTAATAAACTAGGCGGGGTATTAGCCTTGTTATCCTCCATCCTAGTTCTAATAGTAGTACCAATTCTTCACACCTCAAAACAACGAGGACTTACATTCCGACCTGCCTCCCAACTACTATTCTGAATTTTAGTAGCAGATATACTAGTACTAACATGAATCGGAGGAATACCAGTAGAACATCCGTACATTATCATCGGCCAAGTAGCATCAGTACTTTATTTTTCCCTATTCCTGGTGCTAAACCCCTTAGTCGGCTGACTAGAAAACAAAGTAATAAACTGATACG

>B_barbatula2_gi|73671964|gb|DQ105255.1| ATGGCCACCCTGCGGAAAACACACCCCCTCATCAAAATCGCCAACCATGCACTGGTTGATCTACCAGCCCCATCCAACATCTCAGTATGATGAAACTTCGGATCACTCCTTGGATTATGCCTAGCTACACAAATCCTAACCGGATTATTCCTGGCTATGCACTACACATCTGACATCTCCACCGCCTTCTCCTCAGTGGCACACATCTGCCGCGACGTCAACTACGGATGACTAATCCGAAACATACACGCTAATGGTGCCTCCTTCTTTTTCATCTGCCTTTACATACACATCGCCCGAGGACTATATTATGGGTCCTACCTATACAAAGAAACCTGAAATATTGGGGTAGTCATTTTCCTATTGGTAATAATGACAGCTTTTGTAGGCTATGTATTACCATGGGGTCAAATATCTTTTTGAGGTGCCACAGTCATCACCAACCTATTGTCAGCCGTACCTTATGTAGGAGATGTTTTAGTACAATGGATTTGAGGGGGCTTCTCGGTGGACAATGCAACCCTAACACGATTCTTTGCATTTCACTTTCTCTTCCCATTCGTCATTGCCGGAGCAACCATCCTCCATATGTTGTTTCTTCATGAAACAGGGTCAAACAACCCGACCGGCCTAAACTCAGACGCAGACAAAATCACCTTCCACCCCTATTTCTCCTATAAAGACCTACTTGGCTTCGTAGTTATGCTACTAGCCCTTACATCCTTAGTATTATTTACCCCCGGCCTTTTAGGAGACCCAGAGAACTTCACACCTGCAAACCCCCTAGTCACCCCACCACACATTCAACCTGAATGATATTTCCTTTTCGCCTATGCCATCCTGCGATCGATCCCAAACAAGCTAGGGGGCGTGCTAGCCCTCCTCTTTTCCATCCTAATCCTAATGATTGTACCCATTCTTCACACGTCAAAACAGCGAGGGCTGACATTCCGACCAATTACTCAACTCCTCTTCTGAACCTTGGTCGCGGACATACTTATTTTAACGTGAATCGGGGGCATACCCGTAGAACACCCCTTTATTATCATCGGGCAAATCGCATCCGCCCTATATTTTGCGCTATTCTTAGTCTTTATGCCAATGGCAGGATGACTAGAGAATAAAGCGCTAGAATGAGCT

>C_taenia1_gi|62912083|gb|AY940214.1AAAAACACACCCTTTAATTAAAATTGCTAATGATGCACTAGTTGACCTCCCAGCTCCTTCCAACATTTCAGTATGATGAAATTTTGGTTCACTATTAGGATTATGCCTAATTGCCCAAATCTTAACAGGATTATTTCTAGCTATACATTATACATCCGATATCACCACAGCCTTCTCATCTGTGGCTCACATCTGCCGTGATGTAAATTACGGATGACTTATCCGTAACATTCATGCCAACGGCGCATCATTTTTCTTTATTTGTATTTATATTCACATCGCCCGAGGACTATATTATGGATCTTATCTCTACAAAGAGACTTGAAATATTGGAGTAGTTCTCCTTCTACTCGTTATAATAACAGCATTCGTTGGCTATGTCCTTCCATGAGGTCAAATATCCTTTTGAGGGGCCACAGTAATTACCAATCTCCTATCAGCAGTCCCTTATGTAGGAAATGCCCTAGTCCAGTGAATTTGAGGTGGATTCTCGGTGGATAATGCTACACTAACACGATTCTTTGCCTTCCACTTTCTTCTGCCCTTTATTATTGCTGCGGGCACTATTCTTCACCTACTTTTTCTCCCCGAAACAGGCTCAAACAACCCTATAGGACTAAATTCAGACGCAGATAAAGTATCATTTCACCCATATTTCTCATATAAAGACTTATTAGGCTTTGCAGTAGTCCTTCTGGCATTGACCTCACTTTCACTATTTTCCCCTAATCTTCTTGGAGACCCCGATAACTTCACCCCCGCAAATCCCCTAGTGACACCTCCCCATATTAAACCAGAATGATATTTTCTATTTGCATACGCTATCCTTCGATCAATCCCCAATAAACTAGGAGGAGTTCTAGCCTTATTATTTTCAATCCTAGTTTTAATAGTAGTACCTATTCTTCATACGTCAAAACAACGAGGCCTAGCATTCCGACCAATCACTCAATTCCTCTTTTGAACCCTAGTTGCAGACATACTTATCCTTACATGAATTGGAGGAATACCAGTAGAACACCCATTCATTATTATTGGACAACTTGCGTCTGTTCTCTATTTTACACTATTTCTAGTACTAATCCCACTGGCGGGATGACTAGAAAACAAGGCATTAGAATGAGCT

>M_fossilis1_gi|117959984|gb|DQ915197.1| ATGGCAAGCCTACGAAAAACACACCCCCTAATTAAAATTGCTAATGATGCACTTATTGATCTCCCAGCCCCCTCCAATATTTCAGTATGATGAAATTTTGGCTCACTACTAGGACTATGCTTAGTAGCTCAAATTCTTACAGGACTATTCCTGGCTATGCACTATACATCTGACATTTCCACTGCCTTCTCATCCGTAGCACACATCTGCCGAGATGTGAACTATGGATGATTTATCCGCAACATTCATGCTAACGGAGCATCACTCTTTTTCATCTGCATCTATATTCATATTGCCCGAGGACTATACTATGGGTCATATCTCTATAAAGAGACCTGAAATATTGGAGTTATTCTCCTCCTATTAGTTATAATAACAGCATTTGTAGGCTATGTACTCCCATGGGGGCAAATATCCTTCTGGGGTGCCACAGTAATCACCAACCTTCTATCTGCAGTTCCCTACATAGGAAACACCCTAGTCCAATGAATTTGGGGGGGCTTCTCAGTAGATAATGCCACACTAACACGATTCTTTGCTTTCCACTTCCTCTTCCCATTTATCATTGCTGCAGCCACCATCCTTCACCTACTTTTCCTCCATGAAACAGGCTCAAACAATCCCATGGGTATTAACTCAGACGCAGATAAAGTCTCATTTCACCCCTACTTTTCATATAAAGATTTGCTAGGATTTGCAGTTGTTCTACTTGCCCTTACATCTCTATCACTGTTCTCCCCAAACCTCCTAGGCGACCCGGACAACTTTACCCCCGCCAACCCTTTAGTAACACCCCCTCATATTAAACCAGAATGATACTTTTTATTTGCCTATGCCATCCTGCGGTCAATCCCCAATAAATTAGGTGGAGTCCTGGCCCTATTATTCTCTATCCTGGTCTTAATAGTAGTCCCCATCTTACACACCTCAAAACAACGAAGCTTAACATTCCGACCAATCACCCAATTTCTCTTTTGAGCCTTAGTGGCAGACATACTTATCCTAACATGAATTGGGGGCATGCCGGTAGAACATCCATTTGTCATCATCGGGCAACTCGCATCCCTCCTATACTTTTTATTATTCCTGGCTCTAATCCCACTGGCGGGATGGCTAGAAAACAAGGCAATAGAATGAGC

>B_bjoerkna1_gi|133872335|gb|EF137863.1ATGGCAAGCCTACGAAAAACCCACCCACTAATAAAAATCGCTAATGACGCACTAGTCGACCTCCCAACACCATCTAACATTTCCACACTATGAAACTTCGGATCCCTCCTAGGATTATGTTTAATTACCCAAATCCTCACGGGATTATTTCTAGCCATACACTACACCTCTGATATCTCCACCGCATTTTCATCAGTAACCCACATCTGCCGAGACGTTAACTACGGCTGACTTATTCGAAACTTACATGCTAATGGAGCATCATTCTTCTTTATCTGCCTTTATATACATATTGCACGAGGCCTATACTACGGGTCATATCTTTACAAAGAAACCTGAAATATTGGCGTAGTCCTATTTCTTCTAGTTATAATAACAGCCTTCGTCGGCTACGTACTTCCATGAGGACAAATGTCCTTTTGAGGTGCCACCGTAATTACAAATCTCCTCTCAGCAGTCCCTTATATAGGAGACACTCTTGTCCAATGAATCTGAGGCGGCTTTTCAGTAGACAACGCAACTCTCACACGATTCTTCGCATTCCACTTCCTCCTGCCATTCGTTGTAGCCGGCGCAACCCTCCTACACCTACTATTTCTACACGAAACGGGATCAAATAACCCGACCGGATTAAACTCCGACGCAGATAAAATTTCTTTCCACCCATACTTCTCATATAAAGACCTTCTTGGCTTTGTAATCATGTTACTAGCCCTCACTTCGCTAGCATTATTTTCACCTAATCTACTAGGTGACCCAGAAAATTTTACCCCAGCAAACCCACTCGTGACACCCCCGCATATTCAGCCAGAATGATACTTCTTATTTGCCTACGCTATTCTCCGATCCATTCCAAATAAATTAGGAGGGGTTCTTGCATTATTATTCAGTATTTTAGTGCTAATAGTTGTGCCAATCTTACATACCTCAAAGCAACGAGGACTAACTTTCCGTCCTATAACACAATTCTTATTCTGAACCCTAGTTGCAGACATGGTCATTCTAACATGAATTGGAGGCATACCCGTAGAACACCCATATATTATTATTGGCCAAGTCGCATCCATTCTATACTTTGCACTCTTCCTCATTCTTATTCCACTAGCAGGGTGAATGGAAAACAAAGCATTGAAATGAGCCTGCCCTAGTAGCTTAGTTTTAAAGCATCGGT

>B_bjoerkna2_gi|2281719|emb|Y10442.1ATGGCAAGCCTACGAAAAACCCACCCACTAATAAAAATCGCTAATGACGCACTAGTCGACCTTCCAACACCATCTAACATTTCAGTATTATGAAACTTCGGGTCCCTCCTAGGATTGTGTTTAATTACCCAAATCCTCACGGGATTATTTCTAGCCATACATTACACCTCTGATATTTCCACCGCATTTTCATCAGTAACCCACATCTGCCGAGACGTTAACTACGGCTGGCTCATTCGAAATTTACACGCTAACGGAGCATCATTCTTCTTCATCTGTCTTTATATACATATTGCACGAGGCCTATACTACGGATCATATCTTTACAAAGAAACCTGAAATATTGGTGTAGTCCTATTTCTTCTAGTTATAATGACAGCCTTCGTCGGCTATGTACTTCCATGAGGGCAGATATCTTTTTGAGGTGCCACCGTAATTACAAACCTCCTCTCAGCAGTCCCTTATATAGGCGACACCCTCGTCCAATGAATCTGAGGCGGTTTCTCAGTAGATAACGCAACTCTCACACGATTCTTCGCATTCCACTTCCTTCTACCATTCGTTGTCGCCGGCGCAACCCTCTTACACCTACTATTCCTACACGAAACAGGGTCGAACAACCCAGCCGGCTTAAACTCCGACGCAGATAAAATTTCCTTCCACCCGTACTTCTCATATAAAGACCTTCTTGGCTTTGTAATCATATTACTGGCCCTTACTTCCCTGGCATTATTTTCCCCCAACCTACTAGGTGACCCAGAAAATTTTACCCCAGCAAACCCACTCGTGACACCCCCACATATTCAGCCAGAGTGATATTTCCTATTTGCCTACGCCATTCTCCGATCCATTCCAAATAAACTAGGAGGGGTCCTTGCATTATTATTCAGTATTTTAGTGCTAATAGTTGTGCCAATCTTACATACCTCAAAACAACGAGGACTAACTTTCCGCCCTATAACGCAATTCTTATTTTGAACCTTAGTTGCAGACATAATCATCCTGACATGAATCGGAGGCATACCCGTAGAACACCCATATATTATTATTGGCCAAGTCGCATCCATTTTATATTTTGCACTCTTCCTCATTCTTATCCCGCTAGCAGGATGAATAGAGAACAAAGCACTGAAATGAGCC

>A_brama2_gi|549466030|gb|KF552103.1ATGGCAAGCCTACGAAAAACCCACCCACTAATAAAAATCGCTAATGACGCACTAGTCGACCTCCCAACACCATCTAACATTTCAACACTATGAAACTTCGGATCCCTCCTAGGATTATGTTTAATTACCCAAATCCTCACGGGATTATTTCTAGCCATACACTACACCTCTGATATCTCCACCGCATTTTCATCAGTAACCCACATCTGCCGAGACGTTAACTACGGCTGACTTATTCGAAACTTACATGCTAATGGAGCATCATTCTTCTTTATCTGCCTTTATATACATATTGCACGAGGCCTATACTACGGGTCATATCTTTACAAAGAAACCTGAAATATTGGCGTAGTCCTATTTCTTCTAGTTATAATAACAGCCTTCGTCGGCTACGTACTTCCATGAGGACAAATGTCCTTTTGAGGTGCCACCGTAATTACAAATCTCCTCTCAGCAGTCCCTTATATAGGAGACACTCTTGTCCAATGAATCTGAGGCGGCTTTTCAGTAGACAACGCAACTCTCACACGATTCTTCGCATTCCACTTCCTCCTGCCATTCGTTGTAGCCGGCGCAACCCTCCTACACCTACTATTTCTACACGAAACGGGATCAAATAACCCGACCGGATTAAACTCCGACGCAGATAAAATTTCTTTCCACCCATACTTCTCATATAAAGACCTTCTTGGCTTTGTAATCATGTTACTAGCCCTCACTTCGCTAGCATTATTTTCACCTAATCTACTAGGTGACCCAGAAAATTTTACCCCAGCAAACCCACTCGTGACGCCCCCGCATATTCAGCCAGAATGATACT

>A_bipunctatus2_gi|2281713|emb|Y10445.1ATGGCAAGCCTACGAAAAACTCCCCCACTAATAAAAATCGCGAATGGTGCACTAGTTGACCTTCCAACCCCATCTAATATTTCAGCACTCTGAAACTTCGGCTCCCTGCTAGGGCTGTGTTTAATTACCCAAATTCTAACAGGACTATTTTTAGCCATGCACTATACCTCCGATATCTCAACCGCATTTTCATCAGTAACCCACATCTGTCGAGACGTCAACTACGGCTGACTTATCCGAAGCCTTCACGCTAACGGAGCATCTTTTTTCTTCATCTGCCTTTACTTGCACATTGCACGAGGCCTATATTATGGCTCCTACCTTTACAAAGAAACCTGAAACATCGGCGTAGTCCTATTCCTTCTAGTAATAATGACAGCCTTCGTCGGCTACGTACTTCCATGGGGACAAATATCATTTTGGGGCGCCACCGTCATCACGAATCTCCTTTCAGCAGTACCTTACATGGGAGACATACTTGTTCAATGAATCTGGGGCGGCTTCTCAGTAGATAACGCAACCCTCACACGATTCTTCGCGTTCCACTTCCTCCTCCCATTCGTCGTCGCCGGCGCAACCATTCTCCACTTGCTATTCCTACACGAGACAGGATCAAACAACCCGGCCGGATTAAATTCCGACGCAGACAAAATTTCTTTCCACCCATACTTCTCATATAAAGACCTTCTTGGCTTTGTAATCATGTTACTAGCCCTCACCTCCCTAACGCTATTTTCTCCCAACCTACTAGGTGACCCAGAAAATTTTACCCCTGCGAACCCCCTTGTGACACCCCCACACATCCAACCTGAGTGATACTTCCTGTTTGCCTATGCCATCCTACGATCTATCCCAAATAAACTTGGAGGGGTCCTTGCGCTGCTATTTAGCATCTTGGTGCTAATAGTCGTGCCAATTCTACACACCTCAAAACAACGGGGACTAACTTTCCGCCCGCTAACACAATTCCTATTCTGAACCCTAGTTGCAGATATAATTATTTTAACATGAATTGGGGGCATACCTGTAGAGCACCCGTACATTATTATTGGCCAAATTGCATCAATTTTATACTTTGCTCTTTTTCTCGTTCTTGCCCCACTAGCAGGATGAGTAGAAAATAAAGCACTGAAATGAGCC

>A_bipunctatus_gi|5832982|gb|AF090742.1ATGGCAAGCCTACGAAAAACCCACCCACTAATAAAAATCGCGAATGGTGCACTAGTTGACCTTCCAACCCCCTCTAATATTTCGGCACTTTGAAACTTCGGATCCCTGCTGGGATTATGTTTAATTACCCAAATTCTAACGGGATTGTTTTTAGCCATACACTACACCTCTGATATCTCAACCGCATTTTCATCAGTGACCCACATCTGTCGAGACGTTAACTACGGCTGACTTATCCGAAGCCTTCACGCTAACGGCGCATCTTTTTTCTTCATCTGCCTTTATTTACACATTGCACGAGGCCTATATTATGGCTCCTACCTTTACAAAGAAACCTGAAACATCGGCGTAGTCCTATTTCTTTTAGTGATAATAACAGCCTTCGTCGGCTACGTGCTCCCATGGGGACAAATATCATTTTGAGGTGCCACCGTCATTACAAATCTCCTTTCAGCAGTACCCTACATGGGAGACATACTTGTTCAATGAATCTGAGGCGGCTTCTCAGTAGACAACGCAACCCTCACACGATTCTTCGCGTTCCACTTCCTTCTCCCATCTGTCGTCGCCGGCGCAACCATTCTGCACTTGCTATTTCTACACGAGACGGGGTCGAACAATCCGGCCGGATTAAACTCCGATGCAGACAAGATTTCTTTCCACCCATACTTCTCATATAAAGATCTCCTTGGCTTTGTAATCATGTTACTAGCCCTCACCTCCCTGACCCTATTTTCTCCGAACCTATTAGGTGACCCAGAAAATTTTACCCCTGCGAACCCACTTGTAACGCCCCCACACATCCAACCAGAGTGATACTTCTTGTTTGCCTATGCTATCCTGCGATCTATTCCAAATAAACTTGGAGGGGTCCTTGCGCTACTATTTAGCATCTTGGTATTAATGGTCGTGCCAGTCTTACACACCTCAAAACAACGAGGACTAACTTTCCGCCCTTTGACACAATGCCTATTCTGAACCCTCGTTGCAGATATGATTATCTTAACATGAATTGGGGGCATACCCGTAGAGCACCCGTACATTATTATTGGCCAAATTGCATCAATTTTATACTTTGCACTTTTTCTCGTTCTTGCCCCGCTAGCAGGATGAATAGAAAATAAAGCACTGAAATGAGCCT

>A_alburnus1_gi|386367699|gb|JQ436541.1ATGGCAAGCCTACGAAAAACCCACCCACTAATAAAAATCGCTAATGACGCGCTAGTCGATCTTCCAACACCATCTAACATTTCAGCAATATGAAATTTCGGATCCCTTCTAGGGTTATGTTTAATTACCCAAATCCTAACAGGGTTATTCCTAGCCATACACTACACCTCCGATATCTCAACCGCATTCTCATCAGTCACCCATATTTGCCGGGACGTTAACTACGGCTGGCTCATTCGAAACCTACATGCCAACGGAGCATCCTTCTTCTTCATCTGCCTATATATGCATATCGCACGAGGTCTATATTACGGCTCATATCTTTATAAAGAGACCTGAAACATTGGGGTAGTACTATTTCTTCTGGTTATGATAACAGCCTTCGTGGGCTATGTACTCCCATGAGGACAAATATCCTTTTGAGGCGCTACCGTAATCACGAACCTCCTCTCAGCAGTTCCCTACATGGGAGATACCCTTGTTCAATGAATTTGGGGCGGTTTCTCAGTAGATAACGCGACTCTTACGCGATTCTTCGCGTTCCACTTCCTCCTGCCGTTCGTCGTTGCAGGCGCAACCGTCCTCCACTTACTATTCCTACACGAGACAGGATCAAACAACCCAGCCGGGTTAAACTCTGACGCGGATAAAATTTCTTTCCACCCATACTTCTCCTACAAAGACCTTCTCGGCTTCGTAATCATGCTGCTCGCCCTCACCTCGCTGGCGCTATTTTCCCCCAACCTCCTAGGTGATCCAGAGAACTTTACCCCAGCAAACCCACTTGTGACACCCCCACATATCCAACCAGAGTGATACTTCTTGTTTGCATACGCCATCCTCCGGTCTATTCCTAATAAACTAGGCGGGGTTCTTGCACTACTGTTTAGTATTCTAGTGCTAATAGTTGTGCCAATTCTACATACCTCAAAACAACGAGGACTAACTTTCCGCCCCGTGACACAATTCCTATTTTGAACCCTAGTCGCAGATATGATTATCTTAACATGAATTGGGGGCATGCCTGTAGAGCACCCATACATTATTATTGGTCAGGTCGCATCCGTCCTATACTTTGCACTCTTCCTTATCCTTATTCCACTAGCAGGGTTAATAGAGAATAAAGCATTGAAATGAGCTT

>A_alburnus2_gi|85679841|gb|DQ350254.1AAAACCCACCCACTAATAAAAATCGCTAATGACGCGCTAGTCGATCTTCCAACACCATCTAACATTTCAGCAATGTGAAATTTCGGATCCCTTCTAGGGTTGTGTTTAATTACCCAAATCCTAACAGGACTATTCCTAGCCATGCACTACACCTCTGATATCTCAACCGCATTCTCATCAGTCACCCATATTTGCCGAGACGTTAACTACGGCTGACTCATTCGAAACCTACATGCCAACGGAGCATCCTTCTTCTTTATCTGCCTATATATGCATATTGCACGGGGTCTATATTACGGCTCATACCTTTATAAGGAGACCTGAAACATCGGGGTAGTACTATTTCTTCTGGTTATGATAACAGCCTTCGTGGGCTATGTACTCCCATGAGGACAAATGTCCTTTTGAGGTGCTACCGTAATCACAAATCTCCTCTCAGCAGTTCCCTACATGGGAGACACCCTCGTTCAATGAATTTGAGGCGGTTTCTCAGTAGATAACGCGACTCTTACGCGATTCTTCGCGTTCCACTTTCTCCTGCCGTTCGTCGTTGCAGGCGCAACCGTCCTCCACTTACTATTCCTACACGAGACAGGATCAAACAACCCAGCCGGGTTAAACTCTGACGCGGACAAAATTTCTTTCCACCCATACTTCTCCTACAAAGACCTTCTCGGCTTCGTAATCATGCTGCTGGCCCTCACCTCGCTGGCGCTATTTTCCCCTAACCTCCTAGGTGATCCGGAGAACTTTACCCCAGCAAACCCACTTGTGACACCCCCACATATTCAACCAGAGTGATACTTCTTGTTTGCATACGCCATCCTCCGGTCTATTCCTAATAAACTAGGCGGGGTTCTTGCACTATTATTTAGTATTCTAGTGCTAATAGTTGTGCCAATTCTACACACCTCAAAACAACGAGGACTAACTTTCCGCCCCGTGACGCAATTCCTATTTTGAACCCTAGTCGCAGATATGATTATCTTAACATGAATTGGGGGCATGCCCGTAGAGCACCCATACATTATTATTGGTCAGGTCGCATCCGTCCTATACTTTGCACTCTTCCTTATCCTT

>B_barbus1_gi|462648905|gb|KC465927.1| ATGGCAAGCCTACGAAAAACGCACCCCCTAATTAAAATTGCTAACAACGCACTAGTTGACCTACCAGCACCATCTAATATTTCAGCTTGATGAAATTTTGGTTCTCTTCTGGGACTATGCTTAGCTACTCAAATCCTTACCGGCCTATTCTTAGCCATGCATTACACCTCAGATATTTCAACCGCATTTTCATCAGTTGTCCATATCTGCCGAGATGTAAACTACGGCTGGCTGATCCGTAACATGCACGCCAACGGAGCATCATTCTTTTTCCTTTGCATCTACATACACATTGCCCGAGGACTGTACTACGGCTCTTACCTCTACAAGGAAACCTGAAACATCGGCGTAATTCTCCTACTACTAGTCATAGCGACAGCCTTCGTTGGCTACGTACTTCCATGAGGCCAAATATCTTTCTGAGGCGCTACAGTAATTACAAATCTCCTCTCCGCCGTACCATATATAGGAGACATACTAGTCCAATGAATCTGAGGCGGATTTTCGGTAGATAACGCAACACTGACACGATTCTTCGCATTTCACTTCCTACTACCATTTATTATTGCTGCCGCAACCATTCTACATCTCCTATTCCTCCACGAAACAGGGTCAAATAACCCAATTGGACTAAACTCAGACGCAGACAAAATCTCCTTCCACCCATACTTTACCTACAAAGACCTACTCGGGTTCGTAATTATACTACTAGCCCTTACACTACTAGCATTATTTTCCCCTAACCTACTAGGAGACCCGGAAAACTTTACCCCCGCCAACCCCCTAGTCACCCCTCCACATATCAAACCAGAATGATACTTCCTATTTGCCTATGCTATTTTACGGTCGATCCCGAACAAACTAGGAGGGGTTCTCGCTTTACTATTCTCCATTCTAGTACTAATAGTGGTACCGCTCCTACACACCTCAAAACAACGGGGACTAACCTTCCGCCCAATCACCCAGTTCCTGTTCTGAACCCTAGTAGCAGATATAATTATCTTAACATGAATCGGGGGTATACCAGTAGAACACCCATTCATCATCATCGGACAAATCGCATCCGTCCTATACTTCGCACTATTCCTCATTCTCATTCCACTGGCAGGATGGTTAGAAAATAAAGCACTAGAATGAGCTT

>C_nasus1_gi|18460951|gb|AY026402.1ATGGCAAGCCTACGAAAAACCCACCCACTAATAAAAATCGCTAACGACGCGCTAGTCGACCTCCCAACACCATCTAATATCTCAGTAATGTGAAACTTCGGATCTCTCCTAGGATTATGTTTAATTACCCAAATTCTGACAGGATTATTCTTAGCCATACATTACACCTCTGATATCTCAACCGCATTCTCATCAGTGACCCACATCTGCCGAGATGTCAACTACGGCTGACTTATCCGAAGCCTGCATGCCAATGGAGCATCCTTTTTCTTCATCTGTCTTTACATGCATATCGCACGGGGCCTATATTATGGGTCATACCTTTATAAAGAAACCTGAAACATCGGTGTAGTCCTATTCCTTCTGGTTATGATAACGGCCTTTGTAGGCTACGTTCTTCCGTGGGGACAAATATCCTTTTGAGGTGCTACCGTAATTACAAACCTCCTCTCTGCCGTCCCCTACATGGGGGATACCCTTGTTCAATGAATCTGAGGCGGCTTCTCAGTAGACAATGCGACTCTCACACGGTTCTTCGCATTCCACTTCCTACTACCCTTTGTAGTTGCCGGCGCAACCATCCTGCACTTGTTGTTTTTACACGAAACGGGGTCGAACAACCCGGCCGGACTAAATTCAGACGCCGACAAAATTTCTTTCCACCCGTACTTCTCATATAAAGATCTCCTTGGTTTTGTGGCAATGTTACTAGCCCTTACCTCTCTGACATTATTTTCCCCTAACCTATTAGGTGACCCGGAAAACTTTACCCCAGCAAACCCGCTCGTGACACCACCGCATATCCAGCCAGAATGATACTTCTTATTTGCCTACGCCATCCTTCGGTCTATCCCAAATAAACTAGGAGGGGTTCTTGCACTACTATTCAGCATCCTAGTGCTATTAGTCGTGCCAATTTTACACACATCCAAACAACGAGGACTAACTTTCCGCCCAGTGACCCAATTCCTATTCTGAACCCTGGTTGCAGATATATTTATTTTGACATGAATCGGGGGTATACCCGTAGAACACCCATACATTGTTATTGGCCAAGTCGCATCCATCCTATACTTTGCACTATTCCTCATTCTTGTCCCACTAGCAGGATGGGTGGAAAATAAAGCATTGAAATGAGCC

>C_carpio1_gi|68532783|dbj|AB158807.1ATGGCAAGCCTACGAAAAACACACCCTCTCATTAAAATCGCTAACGACGCACTAGTTGACCTACCAACACCATCCAACATCTCAGCATGATGAAACTTTGGATCCCTCCTAGGACTATGCTTAATTACCCAAATTTTAACCGGCCTATTCCTAGCCATACACTACACCTCAGACATTTCAACCGCATTCTCATCTGTTACCCACATCTGCCGAGACGTAAATTACGGCTGACTAATCCGTAATGTACACGCCAACGGAGCATCATTCTTCTTCATTTGCATTTACATACACATCGCCCGAGGCCTATACTACGGATCATACCTTTACAAAGAAACCTGAAACATTGGTGTAGTCCTTCTACTACTAGTCATGATAACAGCCTTCGTTGGCTATGTTCTTCCATGAGGACAAATATCCTTTTGAGGCGCCACAGTAATCACAAACCTCCTATCTGCCGTACCATACATGGGAGACATGTTAGTCCAATGAATCTGAGGTGGGTTCTCAGTAGACAATGCAACACTAACACGATTCTTCGCATTCCACTTCCTACTACCATTTGTTATTGCCGCCGCAACCATCATCCACCTACTGTTCCTCCACGAAACAGGATCAAACAACCCGATCGGACTAAACTCAGACGCAGACAAAGTCTCTTTCCACCCGTACTTCTCATACAAAGACCTCCTTGGGTTCGTAATTATACTCCTAGCTCTTACACTACTAGCACTATTCTCCCCTAACTTACTAGGAGACCCAGAAAACTTCACCCCCGCAAACCCTCTAGTCACACCACCCCACATCAAACCAGAATGATACTTCCTATTTGCCTACGCCATCCTACGATCAATTCCAAACAAACTCGGAGGTGTCCTTGCACTCCTATTCTCCATTCTGGTATTAATAGTAGTACCACTACTACACACCTCAAAACAACGAGGACTAACATTCCGCCCCATCACCCAATTCCTATTCTGAACCCTAGTAGCGGACATAATTATCCTAACATGAATTGGAGGCATACCAGTAGAACATCCCTTCATCATTATTGGACAAATTGCATCCGTCCTATACTTCGCACTATTCCTCATTTTTATGCCACTAGCAGGATGGTTAGAAAATAAAGCACTAAAATGAGCTT

>G_gobio1_gi|37904500|gb|AY426592.1ATGGCAAGCCTACGAAAAACCCACCCCCTAATAAAAATCGCTAATGACGCACTAGGTGACCTACCAACACCATCCAATATTTCAGTCTGATGAAACTTTGGGTCCCTCCTCGGACTCTGTCTAATTACGCAAATCTTAACAGGACTGTTCCTGGCTATGCACTATACCTCAGACATTTCAACTGCATTCTCATCAGTTGCCCACATCTGCCGGGACGTAAACTACGGCTGATTTATTCGTAACGTACACGCCAACGGAGCTTCATTCTTTTTCATCTGCATTTACATGCACGTAGCCCGAGGCCTCTATTACGGATCCTACCCCTACAAAGAAACCTGAAACATTGGAGTGGTTCTGCTGCTGCTAGTTATAATAACGGCCTTCGTTGGCTATGTTCTCCCATGGGGTCAAATATCCTTCTGAGGTGCCACAGTCATTACTAACCTTTTATCAGCAGTCCCTTATATGGGGGACACCCTTGTCCAATGAATTTGAGGCGGGTTTTCAGTTGATAACGCAACACTAACACGATTCTTCGCTTTTCACTTTCTCCTACCATTTGTTATTGCCGCCGCAACCGTCCTCCACCTACTGTTTTTGCACGAGACAGGATCAAATAACCCAGCCGGACTAAACTCTGACGCAGACAAAATCTCATTCCACCCATACTTCTCTTACAAGGACCTTCTTGGTTTTGTCCTAATACTCCTAGCTCTTACATCATTAGCACTGTTCTCCCCTAACTTGCTTGGGGACCCGGACAACTTCACCCCCGCAAACCCGATAGTTACCCCTCCCCACATTAAACCCGAGTGGTATTTCCTATTTGCTTACGCCATTCTACGATCTATCCCTAACAAATTAGGAGGCGTCCTTGCACTATTGTTTTCTATTCTGATTCTCATGGTGGTCCCAATTTTACACACCTCGAAACAACGAGGACTAACCTTCCGCCCCCTAACACAATTCTTATTCTGAACCCTCGTAGCAGACATACTTATCCTAACATGAATTGGAGGCATGCCGGTAGAACATCCATATGTTGTCATCGGCCAAGTCGCTTCAATTCTGTATTTTGCACTTTTCCTTGTCCTTGTCCCAATGGCAGGCTGACTGGAAAATAAAGCATTAAAATGAGCTT

>L_delineatus1_gi|2281725|emb|Y10447.1ATGGCAAGCCTACGAAAAACCCACCCACTAATAAAAATCGCTAATGACGCTCTAGTTGACCTTCCAACACCATCTAATATTTCAGCAATATGAAACTTTGGATCCCTTTTAGGATTATGCTTAATTGCCCAAATCCTAACGGGATTATTCCTGGCCATGCACTACACCTCTGACATCTCGACCGCATTCTCATCAGTTACTCACATCTGCCGAGACGTCAACTACGGTTGGCTCATTCGAAATCTACATGCTAATGGGGCATCCTTCTTCTTCATCTGTCTGTATATACACATCGCACGAGGTTTATACTACGGCTCATATCTTTATAAAGAGACCTGAAATATTGGAGTAGTACTATTTCTTCTGGTTATGATGACAGCCTTCGTGGGCTATGTATTACCATGAGGACAAATGTCCTTCTGGGGCGCTACCGTGATTACAAACCTCCTCTCAGCGGTCCCCTACATAGGAGACACCCTTGTTCAATGAATCTGGGGTGGTTTCTCAGTAGATAATGCAACCCTCACACGATTCTTCGCATTCCACTTCCTCTTACCTTTCGTTGTTGCGGGCGCGACCGTCCTCCACCTTCTATTCCTACACGAGACAGGGTCGAATAACCCGGCCGGACTAAATTCTGACGCGGACAAAATTTCTTTCCACCCCTACTTCTCTTACAAAGACCTTCTCGGCTTCGTAATTATATTGCTAGCCCTCACCTCGCTGGCGCTGTTCTCGCCCAACCTTTTAGGTGATCCAGAAAACTTTACCCCAGCAAACCCTCTCGTGACACCCCCACATATCCAGCCAGAATGATACTTCTTATTTGCGTATGCCATCCTCCGGTCTATTCCTAATAAACTAGGCGGGGTTCTTGCTCTATTATTCAGTATTCTGGTGCTAATAGTTGTGCCGATTATACATACCTCAAAACAACGAGGACTAACCTTCCGTCCCGTGACCCAATTCCTATTCTGAACCCTAGGTGCAGATATGATCATCTTGACATGAATTGGAGGCATACCCGTAGAGCACCCGTATGTTATTATCGGTCAAGGCGCATCCATCCTATACTTTGCACTCTTCCTGATTCTTATCCCACTAGCAGGATTAATGGAAAATAAAGCATTGAAATGAGCT

>L_cephalus1_gi|12657413|emb|AJ252807.1ATGGCAAGCCTACGAAAAACCCACCCACTAATGAAAATCGCCAATGGCGCACTAGTCGACCTTCCAACACCATCTAATATCTCAGCACTATGAAACTTCGGGTCTCTCCTAGGATTATGTTTAATTACCCAAATCCTAACGGGATTATTCTTAGCGATGCACTACACCTCCGACATCTCAACCGCATTTTCATCAGTAACCCACATCTGCCGAGATGTAAACTACGGCTGACTTATTCGAAGTCTGCATGCCAATGGAGCCTCCTTCTTCTTCATCTGTCTTTACATGCACATTGCACGAGGACTATACTATGGCTCATACCTATATAAAGAAACCTGAAACATTGGTGTAGTCCTGTTCCTCCTAGTTATAATAACAGCCTTTGTCGGCTACGTACTTCCGTGGGGACAGATGTCTTTTTGAGGCGCCACGGTAATTACGAACCTGCTCTCAGCGGTCCCTTACATAGGGGACATGCTTGTTCAATGAATCTGAGGTGGTTTCTCAGTAGATAATGCAACCCTTACCCGATTCTTCGCATTCCACTTCCTCCTACCGTTTGTCGTCGCAGGCGCAACCATCCTGCACCTGCTGTTTCTACACGAAACAGGATCAAACAACCCAGCCGGACTAAACTCCGACGCCGACAAAATTTCTTTCCACCCATACTTCTCATATAAAGACCTTCTTGGCTTTGTTATCATATTACTAGCCCTTACCTCTCTAGCATTATTTTCTCCCAACCTACTGGGTGACCCAGAAAACTTTACCCCAGCAAACCCACTAGTGACGCCCCCACATATTCAGCCAGAATGGTACTTCTTATTTGCCTACGCCATTCTCCGGTCTATCCCAAACAAACTAGGAGGGGTCCTGGCGCTACTATTCAGTATCCTAGTGCTACTAGTTGTGCCAATCTTACACACCTCAAAACAACGAGGACTAACTTTCCGCCCCGTAACTCAATTCCTATTCTGAACCCTAGTTGCAGATATATTTATCCTGACATGAATTGGGGGCATACCTGTAGAACACCCATATATTATCATTGGCCAAGTCGCATCCATTCTATACTTTGCGCTCTTCCTCGTCCTTGTCCCGCTAGCAGGATGAGTCGAAAATAAAGCACTGAAATGAGCC

>L_cephalus2_gi|41057843|gb|AY509827.1ATGGCAAGCCTACGAAAAACCCACCCACTGATAAAAATCGCCAATGACGCACTAGTCGACCTTCCAACACCATCTAATATCTCAGCACTGTGAAACTTCGGATCTCTTCTAGGATTATGTTTAATTACCCAAATCCTAACAGGACTATTCTTAGCAATGCACTACACCTCCGACATCTCAACCGCATTTTCATCGGTAACCCACATCTGCCGGGATGTTAACTACGGCTGACTTATTCGAAGCCTACATGCCAATGGAGCCTCCTTCTTCTTCATCTGTCTTTATATACACATTGCACGAGGACTATATTATGGCTCATACCTATATAAAGAAACCTGAAACATTGGTGTAGTCCTGTTTCTCCTAGTTATAATGACAGCCTTTGTCGGCTACGTACTTCCGTGAGGACAAATATCCTTTTGGGGTGCCACAGTGATTACAAACCTCCTCTCAGCAGTCCCTTACATGGGGGACACTCTTGTTCAATGAATCTGAGGCGGTTTCTCCGTAGATAACGCAACCCTCACCCGATTCTTCGCATTCCACTTCCTCCTGCCATTTGTCGTCGCAGGCGCAACCATTCTACACCTCCTATTTCTACACGAAACGGGATCAAACAACCCAGCCGGACTAAATTCCGACGCCGACAAAATTTCTTTCCACCCGTACTTCTCATATAAAGACCTTCTTGGCTTTGTTATTATACTACTAGCCCTTACCTCTCTAGCACTATTTTCTCCTAACCTACTAGGTGACCCAGAAAACTTTACCCCAGCAAACCCACTAGTAACACCCCCACATATTCAGCCAGAGTGATACTTCTTATTTGCCTACGCCATTCTCCGATCTATCCCAAACAAACTAGGAGGGGTACTTGCACTACTATTCAGCATCCTAGTGCTAATAGTTGTGCCAATCTTACACACCTCTAAACAACGAGGACTAACTTTCCGCCCCGTAACTCAATTCCTATTCTGAACCCTAGTTGCAGATATATTTATCCTGACATGAATTGGGGGCATACCTGTAGAACACCCATATATTATCATTGGCCAAGTCGCATCCATTCTATATTTTGCACTCTTCCTCATCCTTGTCCCACTAGCAGGATGGGTGGAAAATAAAGCATTAAAATGAGCC

>L_cepahlus3_gi|269997201|gb|GU182336.1ATGGCAAGCCTACGAAAAACCCACCCACTAATAAAAATCGCCAACGACGCGCTAGTCGATCTTCCCACACCATCTAATATCTCTGCATGATGAAACTTTGGATCCCTTCTAGGATTATGCTTAATTACTCAAATCCTAACCGGACTGTTCTTAGCCATGCATTACACTTCTGACATCTCAACCGCATTCTCATCAGTAGTCCACATTTGCCGGGACGTCAATTACGGCTGACTTATCCGCAACCTACACGCCAACGGGGCATCATTCTTTTTTATCTGTATTTATATACACATTGCTCGCGGCCTATACTATGGATCCTACCTTTACAAAGAAACCTGAAATATTGGAGTAGTCCTACTCCTATTAGTTATAATAACGGCCTTCGTTGGCTACGTCCTCCCATGAGGACAAATATCTTTTTGAGGCGCTACCGTAATTACAAACCTACTATCAGCAGTCCCCTACATAGGAGATACTCTCGTCCAATGAATTTGAGGTGGCTTCTCAGTAGATAATGCAACACTAACACGATTTTTCGCATTCCATTTCCTATTACCATTCATCGTCGCCGCCGCAACCCTCCTACACCTACTCTTCCTGCACGAAACAGGATCAAATAACCCAATCGGACTAAACTCCGACGCAGATAAAATTTCCTTCCACCCATACTTCTCATATAAAGACCTTCTTGGCTTTGTTATTATACTACTAGCCCTTACCTCCCTAGCACTATTTTCTCCTAACCTACTAGGTGACCCAGAAAACTTTACCCCAGCAAACCCACTAGTAACACCCCCACATATTCAGCCAGAGTGATACTTCTTATTTGCCTACGCCATTCTCCGATCTATCCCAAACAAACTAGGAGGGGTACTTGCACTACTATTCAGCATCCTAGTGCTAATAGTTGTGCCAATCTTACACACCTCTAAACAACGAGGACTAACTTTCCGCCCCGTAACTCAATTCCTATTCTGAACCCTAGTTGCAGATATATTTATCCTGACATGAATTGGGGGCATACCTGTAGAACACCCATACATTATCATTGGCCAAGTCGCATCCATTCTATATTTTGCACTCTTCCTCATCCTTGTCCCACTAGCAGGATGAGTGGAAAATAAAGCATTAAAATGAGCCT

>L_leuciscus1_gi|307090186|gb|HM560101.1ATGGCAAGCCTACGAAAAACCCATCCGTTAATAAAAATCGCTAATGACGCACTAGTCGACCTTCCAACCCCATCCAACATTTCAGCCCTATGAAACTTCGGGTCCCTCCTAGGGTTATGTTTAATTACCCAAATCCTAACAGGATTATTTTTAGCCATGCACTACACCTCTGATATTTCGACCGCATTTTCATCAGTAACTCACATTTGTCGGGACGTTAACTACGGCTGGCTTATCCGAAACCTGCACGCCAACGGGGCATCATTCTTCTTCATCTGTATTTATATACATATCGCGCGAGGCCTATATTACGGGTCCTATCTTTATAAGGAGACCTGAAATATCGGTGTAGTACTACTTCTTCTAGTCATAGCAACCGCCTTCGTGGGCTATGTGCTTCCGTGGGGCCAAATATCTTTTTGAGGTGCTACTGTTATTACAAATCTCCTATCAGCAGTCCCTTATATGGGCGACACCCTTGTTCAATGAATTTGAGGCGGCTTCTCAGTAGACAACGCGACCCTCACCCGGTTCTTCGCATTCCACTTCCTCTTACCATTCGTCGTCGCCGGCGCGACCGTCCTACACTTACTATTTCTACACGAAACAGGATCAAATAACCCGGCCGGACTAAATTCTGACGCGGACAAAATTTCCTTCCACCCTTACTTCTCATACAAAGACCTTCTTGGCTTTGTGATCATACTGCTAGCCCTCACCTCTCTGACACTATTTTCCCCTAATCTTCTAGGTGACCCAGAGAATTTTACCCCAGCAAACCCACTCGTGACTCCCCCACATATTCAGCCAGAATGATACTTCCTGTTTGCCTACGCCATTCTCCGGTCCATCCCAAACAAACTAGGAGGGGTTCTTGCACTACTATTTAGCATTCTTGTGCTAATAGTTGTGCCAATTTTACACACCTCAAAACAACGAGGACTAACTTTTCGACCTGTAACCCAATTCCTATTCTGAACCTTAATTGCAGATATAATTATCTTGACATGAATTGGGGGCATACCCGTAGAACACCCATATGTCATTATTGGCCAAGTCGCATCCGTTCTATACTTTGCACTCTTCCTCGTTCTTGTCCCACTAGCAGGATGGGTCGAAAATAAAGCATTGAAATGAGCC

>L_leuciscus3_gi|2281727|emb|Y10449.1ATGGCAAGCCTACGAAAAACCCATCCATTAATAAAAATCGCTAATGACGCACTAGTCGACCTTCCAACCCCATCCAACATTTCAGCACTCTGAAACTTCGGATCCCTCCTAGGATTATGTTTAATTACCCAAATCCTAACGGGATTATTTTTAGCCATACACTACACCTCTGATATTTCGACCGCATTTTCATCAGTAACTCACATTCGTCGGGACGTTAACTACGGCTGGCTCATCCGAAACCTGCACGCCAACGGAGCGTCATTCTTCTTCATCTGTATTTATATGCATATCGCGCGAGGCCTATATTACGGGTCCTATCTTTATAAGGAGACCTGAAATATTGGTGTGGTACTACTTCTTCTAGTCATAGCAACCGCCTTCGTGGGCTATGTACTTCCCTGGGGCCAAATATCTTTTTGAGGTGCTACTGTTATTACAAATCTCCTATCAGCAGTCCCTTATATGGGTGACACCCTTGTTCAATGAATTTGGGGCGGCTTCTCGGTAGACAACGCGACCCTCACCCGGTTCTTCGCATTCCACTTCCTCTTACCATTCGTCGTCGCCGGCGCAACCGTCCTACACTTACTATTTCTGCACGAGACAGGATCAAATAACCCGGCCGGACTAAATTCTGACGCGGACAAAATTTCCTTCCACCCATACTTCTCATATAAAGACCTTCTTGGCTTGGTGATCATACTGCTAGCCCTCACCTCGCTAACGCTATTTTCCCCTAATCTTCTAGGTGACCCAGAGAATTTTACCCCAGCAAACCCACTCGTGACTCCCCCACATATTCAGCCAGAGTGATACTTCCTATTTGCCTACGCCATTCTCCGATCTATCCCAAACAAACTAGGAGGGGTTCTTGCACTACTATTTAGCATCCTTGTGCTAATAGTCGTGCCAATTTTACACACCTCAAAGCAACGAGGACTAACTTTTCGACCTGTGACCCAATTCCTATTCTGAACCTTAATTGCAGATATAATTATCTTAACATGAATTGGAGGCATACCCGTAGAGCACCCATATGTCATTATTGGCCAAATCGCATTCGTTCTATACTTTGCACTCTTCCTCGTTCTTGTTCCACTAGCGGGGTGAGTCGAGAATAAAGCATTGAAATGAGCC

>L_souffia1_gi|29691926|emb|AJ555549.1ACCCACTAATAAAAATCGCTAATGACGCACTAGTCGACCTCCCAACACCATCTAATATTTCAGTAATATGAAACTTCGGTTCTCTTCTAGGATTATGTTTAATTACCCAAATCCTAACAGGATTATTCTTAGCCATGCACTATACCTCTGACATCTCAACCGCATTCTCATCCGTAACCCACATCTGCCGAGACGTCAACTACGGCTGACTTATCCGGAACCTACATGCTAACGGGGCATCCTTCTTCTTCATCTGCCTTTACATGCACATCGCACGAGGCCTGTATTATGGGTCATACCTTTATAAAGAGACCTGAAGCATTGGTGTGGTTCTATTCCTTCTGGTTATGATGACAGCCTTCGTCGGCTACGTTCTTCCATGAGGACAAATATCCTTTTGAGGTGCTACCGTAATTACGAACCTCCTCTCAGCAGTCCCTTACATGGGAGATACCCTTGTTCAGTGAATCTGAGGCGGTTTCTCAGTAGATAATGCAACTCTTACGCGGTTCTTCGCATTCCACTTCCTCCTACCATTTGTCATCGCCGGCGCAACCATTCTGCACCTATTATTCTTACACGAAACGGGATCGAACAACCCAGCCGGACTAAATTCCGACGCGGACAAAATTTCTTTCCACCCATACTTCTCATATAAGGACCTTCTTGGCTTTGTGGCAATGTTACTAGCCCTCACCTCTCTAACCTTATTCTCCCCTAACCTCTTAGGTGACCCGGAGAACTTCACCCCAGCAAACCCACTCGTAACGCCTCCACATATCCAGCCAGAGTGGTACTTCTTGTTTGCCTACGCCATCCTCCGATCTATCCCAAATAAGCTAGGAGGGGTTCTTGCACTACTGTTCAGCATCCTGGTGCTAATAGTCGTGCCAATTTTACACACGTCCAAACAA

>P_phoxinus1_gi|209968201|gb|EU755036.1ATGGCAAGCCTACGAAAGACGCATCCACTAATAAAAATCGCCAACGGCGCACTCGTTGACCTCCCAACACCCTCCAATATTTCTGCACTCTGGAACTTTGGTTCTCTTCTAGGATTGTGTTTAATTACCCAAATTCTCACAGGACTATTTCTAGCCATACATTATACATCTGACATCTCAACCGCATTTTCATCGGTCACCCATATCTGCCGAGACGTTAATTATGGGTGACTAATTCGAAATATACACGCCAACGGCGCATCATTCTTCTTCATCTGCATTTATATGCATATTGCCCGAGGCCTTTATTATGGTTCATATCTTTATAAAGAAACCTGAAACATTGGAGTAGTACTACTCCTCCTGGTAATAATAACAGCCTTTGTGGGCTACGTACTTCCATGAGGCCAAATGTCTTTTTGAGGCGCCACAGTAATTACTAACTTGTTATCAGCAGTCCCTTACATAGGAGACATACTGGTTCAATGAATCTGGGGTGGCTTCTCAGTAGATAATGCTACCCTCACGCGATTCTTCGCATTTCACTTCCTATTCCCATTTGTTATTGCCGGCGCGACCGTCCTGCACTTATTGTTTCTACACGAGACAGGATCAAATAACCCCGTTGGATTAAACTCAGATGCAGATAAAATTTCTTTTCACCCCTACTTTTCTTATAAAGACCTTCTAGGGTTTGCAGTGATACTACTAGCTCTTACATCACTAACCCTATTTTCACCAAGTCTACTAGGTGACCCAGAAAATTTTACTCCTGCTAACCCCCTCGTTACCCCGCCTCATATTCAACCAGAATGGTATTTCCTGTTTGCCTACGCTATCTTACGATCTATCCCTAACAAGTTGGGGGGTGTCCTTGCACTATTATTTAGTATCTTGGTATTAATAGTCGTCCCTACTTTACACACCTCAAAACAACGAGGATTAACTTTCCGCCCATTAACCCAATTCTTATTCTGAACCCTACTAGCAGATATAGTCATTTTGACATGAATTGGGGGCATACCCGTAGAACACCCATATATTATTATTGGTCAAGTAGCGTCAATTTTATACTTTGCACTCTTTCTCATCCTCACACCACTGGCAGGATGGCTAGAGAATAAGACATTAAAATGAGCC

>P_phoxinus2_gi|168203857|gb|EU352213.1ATGGCAAGCCTACGAAAGACGCATCCACTAATAAAAATCGCCAACGGCGCACTCGTTGACCTCCCAACGCCCTCTAATATTTCTGCACTCTGGAACTTTGGTTCTCTTCTAGGGTTATGTCTAATTACCCAAATTCTCACAGGATTATTTCTGGCCATGCACTATACATCTGATATCTCCACCGCATTTTCATCAGTCACCCATATCTGCCGAGACGTTAATTATGGATGACTAATCCGAAACATGCACGCCAACGGCGCATCATTCTTCTTCATCTGTATTTATATGCATATCGCCCGTGGTCTTTATTATGGTTCATATCTTTATAAAGAAACCTGAAATATCGGGGTAGTTCTCCTCCTCCTGGTTATAATAACGGCCTTCGTTGGCTACGTCCTTCCCTGAGGCCAAATGTCTTTTTGAGGTGCCACAGTAATTACAAACTTATTATCAGCAGTCCCCTACATGGGGGACATACTGGTTCAATGAATCTGGGGCGGCTTCTCAGTAGATAATGCAACCCTCACGCGATTCTTCGCATTTCACTTCCTATTTCCCTTTGTTATTGCCGGTGCAACCGTCCTGCACTTGTTATTTTTACACGAAACAGGCTCGAATAACCCCGCTGGATTAAACTCGGATGCGGATAAAATTTCTTTTCACCCATACTTTTCTTATAAAGACCTTCTAGGCTTTGTAGTAATACTACTAGCTCTTACATCACTAACCCTGTTTTCACCGAGCCTGTTGGGTGACCCAGAAAATTTTACCCCTGCGAACCCCCTCGTTACCCCGCCTCATATTCAGCCAGAGTGATATTTCCTGTTTGCCTATGCTATCCTACGATCTATAGCTAACAAGTTGGGAGGTGTCCTTGCGCTATTATTTAGTATCTTGGTCCTGATAGTCGTCCCTATTTTACACACCTCCAAGCAACGAGGACTAACTTTCCGCCCATTAACCCAATTTTTATTCTGAACCCTTGTAGCAGATATGCTTATTTTAACATGAATTGGAGGCATACCTGTAGAGCACCCATACATTATTATTGGCCAAGTAGCGTCGATTTTATACTTTGCACTCTTTCTCATCCTCACACCACTGGCAGGATGGCTAGAGAATAAGACATTAAAATGAGCC

>R_amarus1_gi|636792944|dbj|AB366519.1ATGGCAAGCCTACGAAAAACCCATCCACTTATTAAAATCGCCAATGACGCGCTAGTTGACCTACCAACACCCTCTAACATCTCAGTCTGGTGAAATTTTGGATCTCTACTGGGATTATGCTTAATTTCCCAAATCTTGACAGGCCTTTTCTTAGCTATGCACTACACCTCAGACATTTCAACCGCATTTTCTTCAGTAAACCATATCTGCCGCGATGTAAATTATGGCTGACTGATCCGAAATTTACACGCCAACGGCGCATCATTCTTTTTTATCTGCATCTACATACACATCGCCCGCGGTCTGTACTACGGGTCCTATCTCTACAAAGAGACCTGAAATATTGGTGTTATCCTCTTTCTGCTCGTCATAATGACCGCTTTCGTAGGCTACGTTTTACCCTGAGGTCAAATATCATTCTGGGGCGCCACAGTAATTACCAATCTACTCTCAGCGGTCCCTTATATAGGGGACGCCCTGGTTCAATGAATTTGAGGCGGGTTCTCAGTAGACAACGCAACACTAACTCGATTTTTCGCCTTTCACTTCCTTCTTCCATTTGTTATCGTAGCCGCAACCATCCTCCATCTCCTCTTCCTCCACGAAACAGGGTCAAATAACCCCGCGGGATTAAACTCCGACGCAGATAAAATTTCTTTCCACCCCTACTTCTCCTACAAAGATCTGCTAGGATTCGTACTTATACTAATAACCTTAACAGCCTTAGCATTATTTTCACCTAACCTACTAGGTGACCCAGAAAATTTTACTCCTGCCAACCCACTCGTTACACCACCACACATCCAGCCCGAATGATATTTTCTATTTGCCTACGCCATCTTACGATCTATCCCAAACAAACTTGGAGGTGTCCTAGCACTATTATTTTCTATTCTTGTACTTATAGTAGTGCCACTGCTCCATACATCCAAACAACGCGGACTAACCTACCGTCCAATCACTCAATTCTTATTTTGAACATTAGTGGCAGATATAATTATTCTGACATGGATTGGAGGCATACCCGTAGAGCACCCATACGTTATTATTGGGCAAATTGCGTCAGTGCTGTACTTCGCACTTTTCCTCGTCCTCGCGCCATTAGCCGGATGACTAGAAAATAAAGCATTAAAATGAGCTT

>R_rutilus1_gi|207113302|gb|FJ025072.1ATGGCAAGCCTACGAAAAACCCATCCACTAATAAAAATCGCTAATGACGCGCTAGTCGACCTTCCGACACCATCTAACATCTCAGCACTATGAAACTTCGGGTCCCTGCTAGGGTTATGTTTAATTACCCAAATCCTGACAGGACTATTCTTAGCTATACACTATACCTCTGACATCTCAACCGCGTTTTCATCGGTGACCCACATCTGCCGAGACGTCAACTACGGCTGACTTATCCGAAACCTACATGCTAATGGAGCATCCTTCTTCTTCATCTGTCTTTATATACATATCGCACGAGGCCTATATTACGGGTCATACCTTTATAAGGAAACCTGAAACATTGGTGTGGTTCTATTCCTCCTGGTTATAATGACAGCCTTCGTTGGCTACGTACTACCATGGGGGCAAATATCCTTCTGAGGCGCCACCGTAATCACAAACCTCCTCTCAGCGGTCCCTTACATGGGAGATACCCTTGTTCAGTGAATCTGGGGAGGTTTCTCAGTAGATAACGCAACCCTTACACGGTTCTTCGCATTCCACTTTCTCTTTCCATTTGTCGTCGCCGGCGCAACCGTTCTACACTTACTGTTTTTACACGAGACGGGATCAAACAACCCGGTCGGACTAAACTCCGACGCAGATAAAATTTCTTTCCACCCGTATTTTTCATACAAAGACCTCCTTGGCTTTGTAATTATGTTACTAGCTCTCACCTCCCTAACATTATTTTCTCCTAACCTATTAGGTGACCCAGAAAACTTTACGCCAGCAAACCCACTCGTGACACCCCCACATATTCAGCCAGAATGATACTTCTTATTTGCCTACGCCATCCTCCGATCCATCCCGAACAAGCTAGGAGGGGTCCTCGCACTACTATTCAGCATTCTAGTGCTAATAGTTGTCCCAGTCTTACACACCTCAAAACAACGAGGACTAACTTTCCGCCCTGTGACACAATTTTTATTCTGAACCCTAGTTGCAGATATATTTATCTTAACATGAATCGGAGGCATGCCCGTAGAACACCCATATATCATTATTGGCCAAGTTGCATCCATTCTATACTTTGCACTTTTCCTCGTTCTTGTCCCGCTAGCAGGATGAGCGGAAAATAAAGCATTGAAATGAGCC

>S_erythrophthalmus1_gi|214010911|gb|EU856057.1ATGGCAAGCCCACAAAAAACCCACCCGCTAATTAAAATCGCTAATGACGCACTAGTCGATCTCCCAACACCCTCTAATATTTCAGCGCTATGAAACTTCGGGTCCCTCCTAGGACTATGTTTAATTACTCAAATCCTAACAGGGCTGTTTCTAGCCATGCACTATACCTCTGACATCTCAACCGCATTTTCATCAGTAACCCATATTTGCCGAGACGTTAACTACGGCTGACTTATCCGAAGCCTACATGCCAACGGAGCATCCTTCTTCTTCATCTGTCTTTATATACATATCGCACGAGGACTATATTATGGATCATACCTTTACAAAGAAACCTGAAATATTGGTGTAGTCCTATTCCTTTTGGTTATGATGACGGCCTTCGTCGGTTACGTACTTCCATGAGGGCAAATGTCCTTTTGAGGTGCTACCGTAATTACAAACCTCCTCTCAGCAGTCCCCTACATAGGAGATACCCTTGTTCAGTGAATCTGAGGCGGTTTCTCAGTAGACAACGCGACCCTAACACGATTCTTCGCGTTCCACTTCCTACTGCCGTTCGTCGTCGCAGGCGCAACCATCCTACACTTACTGTTTCTACACGAGACAGGATCAAACAACCCAGCCGGACTAAATTCCGACGCGGACAAAATTTCCTTCCACCCGTACTTCTCATACAAGGACCTTCTTGGCTTCGTAATTATGCTGCTTGCCCTTACAGCTTTGGCACTATTTTCCCCAAACCTTTTAGGGGACCCTGAGAATTTTACTCCAGCAAACCCACTCGTGACACCCCCACACATCCAGCCAGAGTGATATTTCTTATTTGCCTACGCCATCCTCCGATCCATCCCAAATAAACTAGGAGGGGTTCTTGCACTATTATTTAGCATCCTAGTGCTAATAGTTGTGCCAATTCTACATACCTCAAAGCAGCGAGGACTAACTTTCCGCCCCGTGACTCAATTTTTATTCTGAACCCTGGTTGCAGACATAATTATCCTAACATGAATTGGGGGCATGCCCGTAGAGCACCCGTACATTGTTATTGGTCAAGTCGCATCCATCTTATACTTTGCACTCTTCCTTATTCTTATTCCACTAGCAGGATTAATGGAAAATAAAGCATTGAAATGAGCC

>T_tinca1_gi|325169898|gb|HM167957.1ATGGCAAGCCTACGAAAAACCCATCCCCTAATTAAAATTGCTAACGATGCACTAGTTGATTTACCAGTACCCTCTAACATCTCAGCATGATGAAACTTCGGATCCCTCCTTGGACTATGCTTAATTATCCAAATCTTAACAGGATTATTTTTAGCTATACATTATACCTCAGATATTTCAACCGCATTCTCGTCAGTAAACCACATTTGCCGTGATGTAAACTATGGCTGACTTATTCGTAACTTACATGCTAATGGGGCATCATTCTTCTTTATCTGCCTTTATATACATATCGCCCGAGGATTATATTACGGATCATACCTTTACAAAGAAACCTGAAATATTGGAGTAGTTCTTTTTCTATTAGTAATAATAACAGCCTTTGTTGGCTACGTCCTGCCATGAGGACAAATATCCTTTTGAGGCGCAACAGTAATTACTAACCTACTATCAGCAGTTCCCTACATAGGAGATGCTTTAGTTCAATGAATCTGAGGGGGCTTCTCAGTAGACAATGCAACACTTACACGATTCTTCGCATTCCACTTCTTACTGCCATTTATTGTTACCGCCGCCACCCTCCTACACCTGCTATTTTTACACGAAACAGGAGCAAACAACCCAACAGGACTAAACTCCGACGCAGACAAAATCTCCTTCCACCCCTACTTTTCATATAAAGACCTTCTAGGGTTCGTAATTATATTATTAGCCCTCACATCACTAGCACTATTCTCTCCAAACTTATTAGGAGACCCAGAAAATTTTACCCCAGCAAACCCCTTAGTCACACCTCCACACATTCAGCCAGAATGATATTTCTTATTTGCCTACGCCATTTTACGATCAATCCCCAACAAGCTAGGAGGTGTTCTTGCACTATTATTCTCTATTTTAGTACTAATGGTGGTACCAATCTTACATACCTCAAAACAACGAGGACTTACATTCCGCCCAATCACTCAATTCTTATTCTGAACCTTGGTAGCAGATATAGTAATCTTAACATGAATCGGTGGTATACCTGTAGAACACCCATATATTATCATTGGTCAGATTGCATCAATTCTATACTTCGCACTTTTCCTTGTTTTTGCCCCCCTCGCAGGATGACTGGAAAATAAAGCACTGAAATGAGCTT

>T_tinca2_gi|444488022|gb|JX974525.1AACAATAATGGCAAGCCTACGAAAAACCCACCCCCTAATTAAAATTGCTAACGATGCACTAGTTGATTTACCAACACCCTCTAACATCTCAGTATGATGAAACTTCGGGTCCCTCCTTGGACTATGCTTAATTATCCAAATCTTAACAGGATTATTTTTAGCTATACATTACACCTCAGATATTTCAACCGCATTCTCGTCAGTAAACCACATTTGCCGTGATGTAAACTATGGCTGACTTATTCGTAACTTACACGCTAATGGGGCATCATTCTTCTTTATCTGCCTTTATATACATATCGCCCGAGGATTATATTACGGATCATACCTTTACAAAGAGACCTGAAATATTGGAGTAGTTCTTTTTCTATTAGTAATAATAACAGCCTTTGTTGGCTACGTCCTGCCATGAGGACAAATATCCTTTTGAGGCGCAACAGTAATTACTAACCTACTATCAGCAGTTCCCTACATAGGAGATGCTTTAGTTCAATGAATCTGAGGTGGCTTCTCAGTAGACAATGCAACACTTACGCGATTCTTCGCATTCCACTTCTTACTCCCATTCATTGTTGCCGCCGCCACCCTCCTACACCTGCTATTTTTACACGAAACAGGATCAAACAACCCAACAGGACTAAACTCCGACGCAGACAAAATCTCCTTCCACCCCTACTTTTCATATAAAGACCTTCTAGGGTTCGTAATTATATTATTAGCCCTCACATCACTAGCACTATTCTCTCCAAACTTATTAGGAGACCCAGAAAATTTTACCCCAGCAAACCCCTTAGTCACACCTCCACACATTCAGCCAGAATGATATTTCTTATTTGCCTACGCCATTTTACGATCAATCCCAAACAAGCTAGGAGGTGTTCTTGCACTATTATTCTCTATTTTAGTACTAATGGTGGTACCGATCTTACATACCTCAAAACAACGAGGACTTACATTCCGCCCAATCACTCAATTCTTATTCTGAACCTTGGTAGCGGATATAGTAATCTTAACATGAATCGGCGGTATACCTGTAGAACACCCATATATCATCATTGGTCAAATTGCATCAATTTTATACTTCGCACTTTTCCTTGTTCTCGCCCCCCTCGCAGGATGACTGGAAAATAAAGCATTGAAATGAGC

>E_lucius1_gi|325610655|gb|HM177470.1ATGACCAGCCTTCGAAAAACCCACCCCGTACTCAAAATTGTTAACGATGCACTAATTGATCTCCCTGCCCCTGCAAACATTTCTATCTGATGAAACTTCGGCTCCCTCCTAGGGCTATGCTTGATTACACAAATCCTAACCGGCTTATTCCTAGCTATACATTACACCTCTGACATCTCCACAGCCTTCTCATCAGTCTGCCACATCTGCCGGGACGTTAACTACGGCTGACTTATCCGAAATATTCACGCTAACGGTGCATCCTTATTCTTCATCTGCATCTACATACACATCGCCCGAGGCCTTTATTATGGCTCTTACTTATACAAAGAAACCTGAAATATCGGAGTTATCCTCCTCCTCCTAACAATAATAACCGCCTTCGTTGGCTATGTTCTGCCCTGAGGACAAATATCTTTTTGAGGCGCAACAGTAATTACTAACCTATTATCAGCTGTTCCCTACATTGGGAATGACCTGGTCCAATGAATCTGAGGCGGCTTCTCCGTCGATAACGCAACCCTTACACGATTCTTCGCATTCCACTTCTTATTTCCCTTCATCATTGCAGCAGCCACAATCCTCCATTTATTATTCCTTCATGAAACTGGCTCAAACAACCCAGTAGGTATTAACTCTGATGCAGATAAAATCCCCTTTCATCCCTACTTCTCCTACAAAGACCTCTTAGGTTTTGTATTTATGTTATTTGGCTTAGCCTCCCTAGCCCTCTTCTCACCCAACCTTCTGGGAGACCCAGACAACTTCATTCCTGCCAACCCCTTGGTTACTCCACCCCATATTAAACCAGAGTGATATTTTTTATTCGCCTATGCGATCCTACGATCAATTCCTAACAAACTAGGAGGAGTCCTTGCCCTACTATTTTCAATCCTGATCCTAATACTAGTACCCATCCTCCACACCTCTAAGCAGCGCGGAATCACTTTCCGCCCCCTTACCCAATTACTATTTTGACTCTTGGTAGCAGATATATTAATCTTAACATGAATTGGAGGAATACCCGTTGAACACCCCTTTATTATTATTGGACAAGTCACATCCGTAATTTATTTTGCCATCTTCCTACTCCTCGCCCCCCTGGCCGGCTGATTAGAGAACAAAACCCTAGAATAA

>L_lota1_gi|77386019|gb|DQ174053.1ATGGCCAGCCTCCGAAAAACCCACCCAATTCTAAAAATTGCTAATAACGCACTAGTAGACCTCCCCGCCCCTTCTAACATCTCAGTTTGATGAAACTTTGGTTCTCTCCTAGGCCTTTGCTTAATTACCCAGATTTTAACAGGCCTATTCTTAGCCATACATTACACCTCAGACATCGAAACAGCCTTTTCATCCGTTGTACACATCTGCCGTGACGTAAACTACGGATGACTAATCCGAAATATACACGCCAACGGTGCCTCTTTTTTCTTTATTTGCCTCTATCTCCACATTGCCCGAGGCTTATATTACGGCTCATACCTATTTATTGAAACATGAAATATCGGGGTTATTTTATTCCTGTTAGTAATAGTAACCTCTTTTGTAGGCTATGTTCTTCCCTGAGGACAAATATCCTTCTGAGGAGCAACCGTAATTACAAACCTAATATCTACTGTACCCTACGTAGGAAATACCCTAGTTCAATGAATTTGAGGAGGCTTCTCAGTTGACAACGCCACCCTTACCCGCTTCTTTGCATTTCATTTTCTATTCCCTTTTATTGTTGCTGCTGTTACAATACTACACCTACTATTTCTTCACGAAACAGGCTCAAATAACCCAACAGGAATTAACTCAAATGCAGACAAAATCCCCTTCCACCCCTACTTTACTTATAAAGACCTTCTAGGTTTTGCCGTAATACTCCTTGGCCTTACTGCCCTTGCCCTCTTCTCCCCCAACCTGCTTGGAGACCCAGACAATTTTACACCCGCTAACCCGATTGTTACCCCTCCACATGTCAAGCCTGAGTGGTATTTCCTATTTGCCTACGCAATTTTACGATCTATTCCTAACAAACTAGGCGGAGTTCTTGCCCTTCTATTTTCAATTCTAATCCTGATAGTTGTACCCTTCCTACACACCTCCAAACAACGAGGCTTGACATTCCGCCCACTCACTCAAGCACTGTTTTGAGTTCTCGTCGCAGACATACTTGTCTTAACATGAATTGGCGGGGTACCAGTAGAACACCCTTTCATCATCATTGGACAAGTAGCATCCGTCCTGTACTTCTCATTATTCCTAGTATTATTTCCCCTTGCAGGAATAACTGAAAATAAAGCCCTTGAATGAAACT

>L_planeri1_gi|261824331|gb|GQ206149.1ATGTCCCACCCACCAACCATTCTTCGAAAAACTCACCCACTCCTATCACTTGGTAATAGCATGTTAGTTGACCTTCCTTCTCCTGCTAATATCTCAGCCTGATGAAATTTTGGCTCACTTTTAAGCCTATGTTTGATCTTACAAATTATTACAGGACTAATTCTTGCGATACACTATACCGCCAACACTGAACTAGCCTTCTCTTCAGTTATACACATTTGTCGTGATGTTAACAACGGATGGCTTATACGAAATCTTCATGCCAATGGGGCCTCTATATTCTTTATTTGTATTTATGCTCACATCGGGCGAGGGATCTACTACGGCTCTTATTTATATAAAGAAACATGAAACGTTGGAGTCATCTTATTTGCATTAACTGCAGCTACTGCTTTCGTTGGTTATGTACTACCATGAGGGCAAATATCCTTCTGAGGGGCAACCGTTATTACAAATTTAATTTCAGCAGTACCTTATGTAGGAGATGATATTGTAGTATGATTATGAGGCGGCTTCTCAGTATCAAACGCCACATTAACCCGGTTTTTTACATTCCATTTTATTTTACCATTTATCCTAGCAGCAATAACTATAATCCATATTATATTCCTCCATCAAACAGGATCTAGTAACCCCCTAGGAATTAACTCTAATTTGGATAAAATTCAATTTCACCCATACTTCTCTTTCAAAGACATTTTTGGCTTTGTTATTTTACTTGGGGTTCTTTTTATAATTTCTCTTTTAGCCCCAAATGCACTAGGTGAGCCAGACAATTTTATTTATGCCAACCCTCTTAGTACCCCACCTCACATTAAGCCAGAATGATACTTCCTGTTCGCCTATGCGATTCTACGATCTATTCCTAATAAATTAGGTGGGGTCATAGCTTTAGCAGCAGCCATCATAATCCTCCTGGTTATCCCCTTTACCCACACCTCTAAACAACGAGGCATTCAATTTCGTCCGCTCGCCCAAGTTACATTTTGGATTCTGATTGCTGATCTAGCGCTACTCACTTGACTAGGGGGAGAACCAGCTGAACACCCATTTATTTTAATAACACAAATTGCATCAACAGTGTACTTTATAATTTTTATTTTAATCTTTCCAATTCTAGGTCGCCTAGAAAA

>T_thymallus_gi|408833805|gb|JX960869.1ATGGCCAACCTCCGAAAAACCCACCCACTCTTAAAAATTGCTAATGACGCACTAGTCGACCTTCCAGCCCCTTCAAACATCTCAGTATGATGAAACTTTGGATCACTATTGGGCTTGTGTCTAGCCACCCAAATCCTCACCGGGCTATTCTTAGCTATACACTACACCTCTGATATTTCAACAGCTTTTTCCTCTGTATGCCACATTTGCCGAGATGTTAGTTACGGATGACTCATCCGAAACATCCACGCTAACGGAGCATCTTTCTTTTTCATTTGCATTTATATACACATTGCCCGAGGACTTTACTACGGCTCATACCTATATAAAGAAACCTGAAACATCGGAGTTGTACTCCTTCTACTAACTATAATGACAGCCTTCGTAGGCTATGTTCTACCATGAGGACAAATATCTTTCTGAGGAGCAACCGTAATTACGAACCTCTTATCAGCCGTCCCTTATGTGGGAGGTGCCCTAGTACAATGAATTTGAGGGGGGTTCTCCGTAGATAACGCCACTTTGACACGGTTCTTTGCCTTCCACTTCTTATTCCCCTTTGTAATTGCAGCTGCCACAGTCCTTCACCTTCTATTCCTTCATGAAACAGGATCCAATAACCCAGCAGGGATTAACTCCGATGCCGATAAAATCTCGTTTCACCCTTACTTCTCATACAAAGACCTCCTTGGATTTGTAGCCATGCTTCTAGGTTTAACATCCCTAGCCCTATTTGCACCTAATCTTCTAGGAGACCCGGACAATTTTACACCAGCCAACCCACTGGTCACCCCTCCCCACATCAAGCCTGAGTGATACTTCTTGTTTGCCTACGCAATCCTACGATCAATCCCCAATAAACTAGGAGGAGTTCTTGCACTACTATTCTCTATTCTGGTCCTAATAGTTGTACCCATCCTTCACACCTCTAAACAACGAGGCCTAACTTTCCGACCACTCACCCAATTCTTGTTCTGAACCCTAGTCGCAGACATACTCATCCTCACCTGAATTGGAGGCATACCTGTAGAACACCCATTTATCATCATCGGTCAAGTCGCCTCTGTAATCTATTTCACTATCTTCCTAATTTTGGCCCCGCTAGCCGGATGAGCCGAAAACAAAGCCCTCGAATGAGCCT

>S_trutta1_gi|1001096|dbj|D58400.1ATGGCCAACCTCCGAAAAACTCACCCCCTCCTAAAAATTGCTAATGACGCACTAGTCGATCTCCCAGCACCATCTAACATCTCAGTTTGATGAAACTTTGGCTCACTCTTAGGCTTATGTCTAGCCACCCAAATTCTTACCGGACTCTTCCTAGCCATACACTACACCTCCGATATCTCAACAGCCTTTTCCTCTGTTTGCCACATTTGCCGAGATGTTAGCTACGGCTGACTCATCCGAAACATTCACGCTAACGGAGCATCTTTCTTCTTTATCTGTATTTATATACATATCGCCCGAGGACTCTACTATGGTTCCTACCTATATAAAGAAACCTGAAATATCGGAGTCGTACTGCTACTTCTCACTATAATAACCGCCTTCGTGGGCTACGTTCTTCCATGAGGGCAGATGTCCTTCTGAGGAGCCACTGTAATTACAAACCTTCTCTCCGCTGTCCCATACGTTGGAGGCGCCCTTGTACAATGAATTTGAGGCGGATTCTCTGTCGACAACGCCACCCTAACACGATTTTTCGCCTTTCACTTCCTATTCCCATTCGTTATTGCAGCTGCCACAGTACTCCACCTTCTATTTTTACATGAAACCGGCTCTAATAACCCAGCAGGTATCAACTCCGATGCCGATAAAATCTCATTCCACCCATACTTCTCATACAAAGACCTCCTTGGATTCGTAGCTATACTACTTGGCCTAACATCATTAGCTCTGTTCGCACCCAACCTCCTCGGAGACCCGGACAATTTTACGCCTGCCAACCCCCTAGTCACCCCACCTCATATCAAGCCCGAATGATACTTCCTATTCGCCTACGCAATCCTTCGCTCCATTCCTAATAAACTAGGCGGAGTACTCGCCCTCTTATTCTCGATCCTGGTCCTTATAGTCGTTCCTATCCTCCATACCTCTAAGCAACGCGGACTAACCTTTCGCCCCCTAACCCAATTCTTATTCTGAACCCTAGTAGCAGACATACTGATCCTCACCTGAATTGGGGGGATACCTGTAGAACACCCATTCATTATCATCGGTCAAGTTGCCTCTGTAATTTACTTTACTATCTTCCTAGTTCTCGCCCCCTTGGCTGGCTGGGCTGAGAATAAAGCTCTTGAATGAACC

CLUSTAL 2.1 multiple sequence alignment_with_Nordic_Gobies_short_20152603

NM = Neogobius melanostomus

NF = Neogobius fluviatilis

PK = Ponticola kessleri

BG = Babka gymnotrachelus

PM = Proterorhinus semilunaris

GC = Gymnocephalus cernuus

PF = Perca fluviatilis

CG = Cottus gobio

GA = Gasterosteus aculeatus

Gobies primer FW = CCTCTAACATTTCTGCC

Gobies primer Rev = GCCACAGCCTTCTCGTC

NM primer FW = TATGTGATGATCGGACAGC

NM primer Rev =AGCGAGCTGACTAGAGAAC

PK primer FW = ACTAGGCCTATGCCTG

PK primer Rev = GCATATTGGACGAGGACTA

NM_gi|671723846|gb|KF549990.1 --------------------------------------------------

NM_gi|168485493|gb|EU331236.1 -------ATG------------GCCAGCCTACGAAAAACACACCCGCTGC 31

NF_gi|257220486|gb|GQ444442.1 -------ATG------------GCCAGCCTACGAAAAACACACCCACTGC 31

NF_gi|671723852|gb|KF549993.1 --------------------------------------------------

BG_gi|526850801|gb|KF415509.1 -------ATG------------GCCAGCCTACGAAAAACACACCCCCTAC 31

BG_gi|188988554|gb|EU444667.1 -------ATG------------GCCAGCGTACGAAAAACACACCCCCTAC 31

PK_gi|589911366|gb|KC886260.1 -------------------------------------------CCCCTGC 7

PM_gi|526851063|gb|KF415640.1 -------ATG------------GCCAGCCTACGAAAAACGCATCCCCTAC 31

PM_gi|188988552|gb|EU444666.1 -------ATG------------GCCAGCCTACGAAAAACACATCCCCTGC 31

G_niger_gi|526850949|gb|KF4155 -------ATG------------GCCCCCCTACGAAAGACTCACCCCCTGC 31

P_minutus_gi|237638806|gb|FJ52 -------ATG------------ACCAGCCTACGGAAAACCCACCCGCTCC 31

GC3_gi|20339610|gb|AF386598.1 -------ATG------------GCAAGCCTCCGAAAAACTCACCCCCTAC 31

PF3_gi|164562184|gb|EU348846.1 -------ATG------------GCAAGCCTCCGAAAAACTCACCCTCAAC 31

CG1_gi|27762429|gb|AY116366.1 -------ATG------------GNCANCCTACGAAAAACCCACCCCTTAC 31

GA1_gi|25140372|gb|AY116004.1 -------ATG------------GCAAGCCTACGAAAAACGCACCCCCTAC 31

L_cephalus1_gi|12657413|emb|AJ -------ATG------------GCAAGCCTACGAAAAACCCACCCACTAA 31

L_cephalus2_gi|41057843|gb|AY5 -------ATG------------GCAAGCCTACGAAAAACCCACCCACTGA 31

L_cepahlus3_gi|269997201|gb|GU -------ATG------------GCAAGCCTACGAAAAACCCACCCACTAA 31

C_nasus1_gi|18460951|gb|AY0264 -------ATG------------GCAAGCCTACGAAAAACCCACCCACTAA 31

L_souffia1_gi|29691926|emb|AJ5 -----------------------------------------ACCCACTAA 9

R_rutilus1_gi|207113302|gb|FJ0 -------ATG------------GCAAGCCTACGAAAAACCCATCCACTAA 31

B_bjoerkna1_gi|133872335|gb|EF -------ATG------------GCAAGCCTACGAAAAACCCACCCACTAA 31

A_brama2_gi|549466030|gb|KF552 -------ATG------------GCAAGCCTACGAAAAACCCACCCACTAA 31

B_bjoerkna2_gi|2281719|emb|Y10 -------ATG------------GCAAGCCTACGAAAAACCCACCCACTAA 31

L_leuciscus1_gi|307090186|gb|H -------ATG------------GCAAGCCTACGAAAAACCCATCCGTTAA 31

L_leuciscus3_gi|2281727|emb|Y1 -------ATG------------GCAAGCCTACGAAAAACCCATCCATTAA 31

A_alburnus1_gi|386367699|gb|JQ -------ATG------------GCAAGCCTACGAAAAACCCACCCACTAA 31

A_alburnus2_gi|85679841|gb|DQ3 ----------------------------------AAAACCCACCCACTAA 16

L_delineatus1_gi|2281725|emb|Y -------ATG------------GCAAGCCTACGAAAAACCCACCCACTAA 31

S_erythrophthalmus1_gi|2140109 -------ATG------------GCAAGCCCACAAAAAACCCACCCGCTAA 31

A_bipunctatus2_gi|2281713|emb| -------ATG------------GCAAGCCTACGAAAAACTCCCCCACTAA 31

A_bipunctatus_gi|5832982|gb|AF -------ATG------------GCAAGCCTACGAAAAACCCACCCACTAA 31

P_phoxinus1_gi|209968201|gb|EU -------ATG------------GCAAGCCTACGAAAGACGCATCCACTAA 31

P_phoxinus2_gi|168203857|gb|EU -------ATG------------GCAAGCCTACGAAAGACGCATCCACTAA 31

T_tinca1_gi|325169898|gb|HM167 -------ATG------------GCAAGCCTACGAAAAACCCATCCCCTAA 31

T_tinca2_gi|444488022|gb|JX974 AACAATAATG------------GCAAGCCTACGAAAAACCCACCCCCTAA 38

G_gobio1_gi|37904500|gb|AY4265 -------ATG------------GCAAGCCTACGAAAAACCCACCCCCTAA 31

B_barbus1_gi|462648905|gb|KC46 -------ATG------------GCAAGCCTACGAAAAACGCACCCCCTAA 31

C_carpio1_gi|68532783|dbj|AB15 -------ATG------------GCAAGCCTACGAAAAACACACCCTCTCA 31

R_amarus1_gi|636792944|dbj|AB3 -------ATG------------GCAAGCCTACGAAAAACCCATCCACTTA 31

B_barbatula2_gi|73671964|gb|DQ -------ATG------------GCCACCCTGCGGAAAACACACCCCCTCA 31

C_taenia1_gi|62912083|gb|AY940 ---------------------------------AAAAACACACCCTTTAA 17

M_fossilis1_gi|117959984|gb|DQ -------ATG------------GCAAGCCTACGAAAAACACACCCCCTAA 31

A_anguilla1_gi|159461951|gb|EU -------ATG------------GCAAACCTACGAAAAACCCACCCACTTC 31

T_thymallus_gi|408833805|gb|JX -------ATG------------GCCAACCTCCGAAAAACCCACCCACTCT 31

S_trutta1_gi|1001096|dbj|D5840 -------ATG------------GCCAACCTCCGAAAAACTCACCCCCTCC 31

E_lucius1_gi|325610655|gb|HM17 -------ATG------------ACCAGCCTTCGAAAAACCCACCCCGTAC 31

L_lota1_gi|77386019|gb|DQ17405 -------ATG------------GCCAGCCTCCGAAAAACCCACCCAATTC 31

L_planeri1_gi|261824331|gb|GQ2 -------ATGTCCCACCCACCAACCATTCTTCGAAAAACTCACCCACTCC 43

NM_gi|671723846|gb|KF549990.1 -----ATCGCAAACCACGCACTAGTAGACCTACCCGCCCCCTCTAACATT 45

NM_gi|168485493|gb|EU331236.1 TTAAAATCGCAAACCACGCACTAGTAGACCTACCCGCCCCCTCTAACATT 81

NF_gi|257220486|gb|GQ444442.1 TTAAAATTGCAAACCATGCACTAGTAGACCTACCCGCCCCCTCTAATATT 81

NF_gi|671723852|gb|KF549993.1 -----ATTGCAAACCACGCACTAGTAGACCTACCCGCCCCCTCCAACATT 45

BG_gi|526850801|gb|KF415509.1 TAAAAATTGCTAACCATGCACTGGTAGACTTACCTGCCCCCTCGAACATT 81

BG_gi|188988554|gb|EU444667.1 TAAAAATTGCTAACCATGCACTGGTAGACTTACCTGCCCCCTCGAACATT 81

PK_gi|589911366|gb|KC886260.1 TTAAAATCGCAAACCATGCACTGGTAGACTTACCTGCCCCCTCAAACATT 57

PM_gi|526851063|gb|KF415640.1 TTAAAATTGCAAACCATGCACTAGTGGATTTACCTGCCCCTTCAAACATT 81

PM_gi|188988552|gb|EU444666.1 TTAAAATTGCAAACCACGCACTAGTGGACCTACCTGCCCCCTCAAATATT 81

G_niger_gi|526850949|gb|KF4155 TTAAAATCGCAAACCACGCACTGGTTGACCTCCCCGCCCCCTCTAACATT 81

P_minutus_gi|237638806|gb|FJ52 TTAAGATTGCAAATGGTGCACTTGTAGACCTCCCTGCACCCTCAAATATC 81

GC3_gi|20339610|gb|AF386598.1 TAAAAATTGCAAACGGCGCACTAGTTGACCTACCCGCCCCCTCTAATATT 81

PF3_gi|164562184|gb|EU348846.1 TAAAAATTGCAAACAACGCACTACTTGACTTACCCGCCCCTTCTAATATT 81

CG1_gi|27762429|gb|AY116366.1 TAAAAATCGCAAACAATGCACTAGTTGACCTTCCAGCCCCCTCAAATATT 81

GA1_gi|25140372|gb|AY116004.1 TAAAAATCGCTAACAATGCACTAGTCGACCTCCCCGCCCCCTCAAATATT 81

L_cephalus1_gi|12657413|emb|AJ TGAAAATCGCCAATGGCGCACTAGTCGACCTTCCAACACCATCTAATATC 81

L_cephalus2_gi|41057843|gb|AY5 TAAAAATCGCCAATGACGCACTAGTCGACCTTCCAACACCATCTAATATC 81

L_cepahlus3_gi|269997201|gb|GU TAAAAATCGCCAACGACGCGCTAGTCGATCTTCCCACACCATCTAATATC 81

C_nasus1_gi|18460951|gb|AY0264 TAAAAATCGCTAACGACGCGCTAGTCGACCTCCCAACACCATCTAATATC 81

L_souffia1_gi|29691926|emb|AJ5 TAAAAATCGCTAATGACGCACTAGTCGACCTCCCAACACCATCTAATATT 59

R_rutilus1_gi|207113302|gb|FJ0 TAAAAATCGCTAATGACGCGCTAGTCGACCTTCCGACACCATCTAACATC 81

B_bjoerkna1_gi|133872335|gb|EF TAAAAATCGCTAATGACGCACTAGTCGACCTCCCAACACCATCTAACATT 81

A_brama2_gi|549466030|gb|KF552 TAAAAATCGCTAATGACGCACTAGTCGACCTCCCAACACCATCTAACATT 81

B_bjoerkna2_gi|2281719|emb|Y10 TAAAAATCGCTAATGACGCACTAGTCGACCTTCCAACACCATCTAACATT 81

L_leuciscus1_gi|307090186|gb|H TAAAAATCGCTAATGACGCACTAGTCGACCTTCCAACCCCATCCAACATT 81

L_leuciscus3_gi|2281727|emb|Y1 TAAAAATCGCTAATGACGCACTAGTCGACCTTCCAACCCCATCCAACATT 81

A_alburnus1_gi|386367699|gb|JQ TAAAAATCGCTAATGACGCGCTAGTCGATCTTCCAACACCATCTAACATT 81

A_alburnus2_gi|85679841|gb|DQ3 TAAAAATCGCTAATGACGCGCTAGTCGATCTTCCAACACCATCTAACATT 66

L_delineatus1_gi|2281725|emb|Y TAAAAATCGCTAATGACGCTCTAGTTGACCTTCCAACACCATCTAATATT 81

S_erythrophthalmus1_gi|2140109 TTAAAATCGCTAATGACGCACTAGTCGATCTCCCAACACCCTCTAATATT 81

A_bipunctatus2_gi|2281713|emb| TAAAAATCGCGAATGGTGCACTAGTTGACCTTCCAACCCCATCTAATATT 81

A_bipunctatus_gi|5832982|gb|AF TAAAAATCGCGAATGGTGCACTAGTTGACCTTCCAACCCCCTCTAATATT 81

P_phoxinus1_gi|209968201|gb|EU TAAAAATCGCCAACGGCGCACTCGTTGACCTCCCAACACCCTCCAATATT 81

P_phoxinus2_gi|168203857|gb|EU TAAAAATCGCCAACGGCGCACTCGTTGACCTCCCAACGCCCTCTAATATT 81

T_tinca1_gi|325169898|gb|HM167 TTAAAATTGCTAACGATGCACTAGTTGATTTACCAGTACCCTCTAACATC 81

T_tinca2_gi|444488022|gb|JX974 TTAAAATTGCTAACGATGCACTAGTTGATTTACCAACACCCTCTAACATC 88

G_gobio1_gi|37904500|gb|AY4265 TAAAAATCGCTAATGACGCACTAGGTGACCTACCAACACCATCCAATATT 81

B_barbus1_gi|462648905|gb|KC46 TTAAAATTGCTAACAACGCACTAGTTGACCTACCAGCACCATCTAATATT 81

C_carpio1_gi|68532783|dbj|AB15 TTAAAATCGCTAACGACGCACTAGTTGACCTACCAACACCATCCAACATC 81

R_amarus1_gi|636792944|dbj|AB3 TTAAAATCGCCAATGACGCGCTAGTTGACCTACCAACACCCTCTAACATC 81

B_barbatula2_gi|73671964|gb|DQ TCAAAATCGCCAACCATGCACTGGTTGATCTACCAGCCCCATCCAACATC 81

C_taenia1_gi|62912083|gb|AY940 TTAAAATTGCTAATGATGCACTAGTTGACCTCCCAGCTCCTTCCAACATT 67

M_fossilis1_gi|117959984|gb|DQ TTAAAATTGCTAATGATGCACTTATTGATCTCCCAGCCCCCTCCAATATT 81

A_anguilla1_gi|159461951|gb|EU TAAAAATTGCTAACGATGCCCTAGTGGATCTACCAACCCCATCCAATATT 81

T_thymallus_gi|408833805|gb|JX TAAAAATTGCTAATGACGCACTAGTCGACCTTCCAGCCCCTTCAAACATC 81

S_trutta1_gi|1001096|dbj|D5840 TAAAAATTGCTAATGACGCACTAGTCGATCTCCCAGCACCATCTAACATC 81

E_lucius1_gi|325610655|gb|HM17 TCAAAATTGTTAACGATGCACTAATTGATCTCCCTGCCCCTGCAAACATT 81

L_lota1_gi|77386019|gb|DQ17405 TAAAAATTGCTAATAACGCACTAGTAGACCTCCCCGCCCCTTCTAACATC 81

L_planeri1_gi|261824331|gb|GQ2 TATCACTTGGTAATAGCATGTTAGTTGACCTTCCTTCTCCTGCTAATATC 93

NM_gi|671723846|gb|KF549990.1 TCTGCCTGATGAAACTTCGGCTCTCTCCTAGGCCTATGTTTAATTGCCCA 95

NM_gi|168485493|gb|EU331236.1 TCTGCCTGATGAAACTTCGGCTCTCTCCTCGGCCTATGTTTAATTGCTCA 131

NF_gi|257220486|gb|GQ444442.1 TCGGCCTGATGAAATTTCGGCTCCCTCCTCGGCCTATGCTTAATTGCCCA 131

NF_gi|671723852|gb|KF549993.1 TCAGCCTGATGAAACTTCGGCTCCCTCCTCGGCCTATGCTTAATTGCCCA 95

BG_gi|526850801|gb|KF415509.1 TCTGCCTGATGGAACTTTGGTTCACTACTGGGCTTATGCCTTATTGCTCA 131

BG_gi|188988554|gb|EU444667.1 TCTGCCTGATGGAACTTTGGTTCACTACTGGGCTTATGCCTTATTGCTCA 131

PK_gi|589911366|gb|KC886260.1 TCTGCCTGATGGAACTTTGGCTCTCTACTAGGCCTATGCCTGATTGCTCA 107

PM_gi|526851063|gb|KF415640.1 TCTGCCTGATGAAACTTCGGCTCTCTTCTGGGCTTATGTCTTATTGCTCA 131

PM_gi|188988552|gb|EU444666.1 TCCGCCTGATGAAACTTCGGCTCTCTCCTTGGCTTATGCTTAATTGCTCA 131

G_niger_gi|526850949|gb|KF4155 TCTGCCTGATGGAACTTCGGCTCCCTCCTCGGGCTCTGCCTTATTGCCCA 131

P_minutus_gi|237638806|gb|FJ52 TCTGCCTGATGGAATTTTGGCTCTTTACTGGGTATCTGCCTTATTGCTCA 131

GC3_gi|20339610|gb|AF386598.1 TCAGTATGATGAAACTTTGGTTCCTTACTTGGCCTCTGTTTAATCACCCA 131

PF3_gi|164562184|gb|EU348846.1 TCAGTATGATGAAATTTTGGTTCCCTACTTGGCCTCTGTTTAATTACCCA 131

CG1_gi|27762429|gb|AY116366.1 TCGGTATGATGAAACTTTGGTTCCCTCCTCGGTCTTTGCTTAATTATCCA 131

GA1_gi|25140372|gb|AY116004.1 TCAGTATGATGAAACTTTGGTTCCCTCCTTGGACTTTGCTTAATTATCCA 131

L_cephalus1_gi|12657413|emb|AJ TCAGCACTATGAAACTTCGGGTCTCTCCTAGGATTATGTTTAATTACCCA 131

L_cephalus2_gi|41057843|gb|AY5 TCAGCACTGTGAAACTTCGGATCTCTTCTAGGATTATGTTTAATTACCCA 131

L_cepahlus3_gi|269997201|gb|GU TCTGCATGATGAAACTTTGGATCCCTTCTAGGATTATGCTTAATTACTCA 131

C_nasus1_gi|18460951|gb|AY0264 TCAGTAATGTGAAACTTCGGATCTCTCCTAGGATTATGTTTAATTACCCA 131

L_souffia1_gi|29691926|emb|AJ5 TCAGTAATATGAAACTTCGGTTCTCTTCTAGGATTATGTTTAATTACCCA 109

R_rutilus1_gi|207113302|gb|FJ0 TCAGCACTATGAAACTTCGGGTCCCTGCTAGGGTTATGTTTAATTACCCA 131

B_bjoerkna1_gi|133872335|gb|EF TCCACACTATGAAACTTCGGATCCCTCCTAGGATTATGTTTAATTACCCA 131

A_brama2_gi|549466030|gb|KF552 TCAACACTATGAAACTTCGGATCCCTCCTAGGATTATGTTTAATTACCCA 131

B_bjoerkna2_gi|2281719|emb|Y10 TCAGTATTATGAAACTTCGGGTCCCTCCTAGGATTGTGTTTAATTACCCA 131

L_leuciscus1_gi|307090186|gb|H TCAGCCCTATGAAACTTCGGGTCCCTCCTAGGGTTATGTTTAATTACCCA 131

L_leuciscus3_gi|2281727|emb|Y1 TCAGCACTCTGAAACTTCGGATCCCTCCTAGGATTATGTTTAATTACCCA 131

A_alburnus1_gi|386367699|gb|JQ TCAGCAATATGAAATTTCGGATCCCTTCTAGGGTTATGTTTAATTACCCA 131

A_alburnus2_gi|85679841|gb|DQ3 TCAGCAATGTGAAATTTCGGATCCCTTCTAGGGTTGTGTTTAATTACCCA 116

L_delineatus1_gi|2281725|emb|Y TCAGCAATATGAAACTTTGGATCCCTTTTAGGATTATGCTTAATTGCCCA 131

S_erythrophthalmus1_gi|2140109 TCAGCGCTATGAAACTTCGGGTCCCTCCTAGGACTATGTTTAATTACTCA 131

A_bipunctatus2_gi|2281713|emb| TCAGCACTCTGAAACTTCGGCTCCCTGCTAGGGCTGTGTTTAATTACCCA 131

A_bipunctatus_gi|5832982|gb|AF TCGGCACTTTGAAACTTCGGATCCCTGCTGGGATTATGTTTAATTACCCA 131

P_phoxinus1_gi|209968201|gb|EU TCTGCACTCTGGAACTTTGGTTCTCTTCTAGGATTGTGTTTAATTACCCA 131

P_phoxinus2_gi|168203857|gb|EU TCTGCACTCTGGAACTTTGGTTCTCTTCTAGGGTTATGTCTAATTACCCA 131

T_tinca1_gi|325169898|gb|HM167 TCAGCATGATGAAACTTCGGATCCCTCCTTGGACTATGCTTAATTATCCA 131

T_tinca2_gi|444488022|gb|JX974 TCAGTATGATGAAACTTCGGGTCCCTCCTTGGACTATGCTTAATTATCCA 138

G_gobio1_gi|37904500|gb|AY4265 TCAGTCTGATGAAACTTTGGGTCCCTCCTCGGACTCTGTCTAATTACGCA 131

B_barbus1_gi|462648905|gb|KC46 TCAGCTTGATGAAATTTTGGTTCTCTTCTGGGACTATGCTTAGCTACTCA 131

C_carpio1_gi|68532783|dbj|AB15 TCAGCATGATGAAACTTTGGATCCCTCCTAGGACTATGCTTAATTACCCA 131

R_amarus1_gi|636792944|dbj|AB3 TCAGTCTGGTGAAATTTTGGATCTCTACTGGGATTATGCTTAATTTCCCA 131

B_barbatula2_gi|73671964|gb|DQ TCAGTATGATGAAACTTCGGATCACTCCTTGGATTATGCCTAGCTACACA 131

C_taenia1_gi|62912083|gb|AY940 TCAGTATGATGAAATTTTGGTTCACTATTAGGATTATGCCTAATTGCCCA 117

M_fossilis1_gi|117959984|gb|DQ TCAGTATGATGAAATTTTGGCTCACTACTAGGACTATGCTTAGTAGCTCA 131

A_anguilla1_gi|159461951|gb|EU TCAGCATGATGAAATTTTGGCTCTCTTCTAGGATTATGTCTTATTTCACA 131

T_thymallus_gi|408833805|gb|JX TCAGTATGATGAAACTTTGGATCACTATTGGGCTTGTGTCTAGCCACCCA 131

S_trutta1_gi|1001096|dbj|D5840 TCAGTTTGATGAAACTTTGGCTCACTCTTAGGCTTATGTCTAGCCACCCA 131

E_lucius1_gi|325610655|gb|HM17 TCTATCTGATGAAACTTCGGCTCCCTCCTAGGGCTATGCTTGATTACACA 131

L_lota1_gi|77386019|gb|DQ17405 TCAGTTTGATGAAACTTTGGTTCTCTCCTAGGCCTTTGCTTAATTACCCA 131

L_planeri1_gi|261824331|gb|GQ2 TCAGCCTGATGAAATTTTGGCTCACTTTTAAGCCTATGTTTGATCTTACA 143

NM_gi|671723846|gb|KF549990.1 AATTGTAACAGGGCTTTTTCTAGCCATACACTACACCTCGGATATCGCCA 145

NM_gi|168485493|gb|EU331236.1 AATTGTAACAGGGCTTTTTCTAGCCATACACTACACCTCGGATATCGCCA 181

NF_gi|257220486|gb|GQ444442.1 AATTGTTACAGGGCTTTTCTTAGCCATACACTACACCTCTGATATTGCCA 181

NF_gi|671723852|gb|KF549993.1 AATTGTGACGGGACTTTTTTTAGCTATACACTATACATCTGATATTGCCA 145

BG_gi|526850801|gb|KF415509.1 AATTATCACAGGACTATTTCTAGCTATACATTATACCTCTGATATTGCCA 181

BG_gi|188988554|gb|EU444667.1 AATTATCACAGGACTATTTCTAGCTATACATTATACCTCTGATATTGCCA 181

PK_gi|589911366|gb|KC886260.1 AATTGTCACAGGACTATTTTTAGCTATACATTATACCTCTGATACTGCCA 157

PM_gi|526851063|gb|KF415640.1 AATTATTACAGGACTATTTTTAGCTATACACTATACCTCTGATATCGCCA 181

PM_gi|188988552|gb|EU444666.1 AATTGTTACAGGACTGTTTTTAGCTATACACTACACCTCTGATATCGCCA 181

G_niger_gi|526850949|gb|KF4155 AATCCTCACTGGCCTTTTCTTAGCCATACATTACACCGCAGACATTACAA 181

P_minutus_gi|237638806|gb|FJ52 AATTCTGACTGGCCTCTTTCTGGCGATACACTACACATCTGATATCGCCA 181

GC3_gi|20339610|gb|AF386598.1 AATCCTTACAGGCCTCTTTTTAGCAATACACTATACCGCAGACATCGCCA 181

PF3_gi|164562184|gb|EU348846.1 AATCCTAACTGGCCTTTTTCTGGCAATACACTATACCGCAGACATCGCAA 181

CG1_gi|27762429|gb|AY116366.1 AATCCTAACCGGGCTCTTCCTCGCCATACACTACACCTCTGACATCGCAA 181

GA1_gi|25140372|gb|AY116004.1 AATTCTCACTGGGCTTTTCCTTGCAATACACTACACTTCCGATATTGCTA 181

L_cephalus1_gi|12657413|emb|AJ AATCCTAACGGGATTATTCTTAGCGATGCACTACACCTCCGACATCTCAA 181

L_cephalus2_gi|41057843|gb|AY5 AATCCTAACAGGACTATTCTTAGCAATGCACTACACCTCCGACATCTCAA 181

L_cepahlus3_gi|269997201|gb|GU AATCCTAACCGGACTGTTCTTAGCCATGCATTACACTTCTGACATCTCAA 181

C_nasus1_gi|18460951|gb|AY0264 AATTCTGACAGGATTATTCTTAGCCATACATTACACCTCTGATATCTCAA 181

L_souffia1_gi|29691926|emb|AJ5 AATCCTAACAGGATTATTCTTAGCCATGCACTATACCTCTGACATCTCAA 159

R_rutilus1_gi|207113302|gb|FJ0 AATCCTGACAGGACTATTCTTAGCTATACACTATACCTCTGACATCTCAA 181

B_bjoerkna1_gi|133872335|gb|EF AATCCTCACGGGATTATTTCTAGCCATACACTACACCTCTGATATCTCCA 181

A_brama2_gi|549466030|gb|KF552 AATCCTCACGGGATTATTTCTAGCCATACACTACACCTCTGATATCTCCA 181

B_bjoerkna2_gi|2281719|emb|Y10 AATCCTCACGGGATTATTTCTAGCCATACATTACACCTCTGATATTTCCA 181

L_leuciscus1_gi|307090186|gb|H AATCCTAACAGGATTATTTTTAGCCATGCACTACACCTCTGATATTTCGA 181

L_leuciscus3_gi|2281727|emb|Y1 AATCCTAACGGGATTATTTTTAGCCATACACTACACCTCTGATATTTCGA 181

A_alburnus1_gi|386367699|gb|JQ AATCCTAACAGGGTTATTCCTAGCCATACACTACACCTCCGATATCTCAA 181

A_alburnus2_gi|85679841|gb|DQ3 AATCCTAACAGGACTATTCCTAGCCATGCACTACACCTCTGATATCTCAA 166

L_delineatus1_gi|2281725|emb|Y AATCCTAACGGGATTATTCCTGGCCATGCACTACACCTCTGACATCTCGA 181

S_erythrophthalmus1_gi|2140109 AATCCTAACAGGGCTGTTTCTAGCCATGCACTATACCTCTGACATCTCAA 181

A_bipunctatus2_gi|2281713|emb| AATTCTAACAGGACTATTTTTAGCCATGCACTATACCTCCGATATCTCAA 181

A_bipunctatus_gi|5832982|gb|AF AATTCTAACGGGATTGTTTTTAGCCATACACTACACCTCTGATATCTCAA 181

P_phoxinus1_gi|209968201|gb|EU AATTCTCACAGGACTATTTCTAGCCATACATTATACATCTGACATCTCAA 181

P_phoxinus2_gi|168203857|gb|EU AATTCTCACAGGATTATTTCTGGCCATGCACTATACATCTGATATCTCCA 181

T_tinca1_gi|325169898|gb|HM167 AATCTTAACAGGATTATTTTTAGCTATACATTATACCTCAGATATTTCAA 181

T_tinca2_gi|444488022|gb|JX974 AATCTTAACAGGATTATTTTTAGCTATACATTACACCTCAGATATTTCAA 188

G_gobio1_gi|37904500|gb|AY4265 AATCTTAACAGGACTGTTCCTGGCTATGCACTATACCTCAGACATTTCAA 181

B_barbus1_gi|462648905|gb|KC46 AATCCTTACCGGCCTATTCTTAGCCATGCATTACACCTCAGATATTTCAA 181

C_carpio1_gi|68532783|dbj|AB15 AATTTTAACCGGCCTATTCCTAGCCATACACTACACCTCAGACATTTCAA 181

R_amarus1_gi|636792944|dbj|AB3 AATCTTGACAGGCCTTTTCTTAGCTATGCACTACACCTCAGACATTTCAA 181

B_barbatula2_gi|73671964|gb|DQ AATCCTAACCGGATTATTCCTGGCTATGCACTACACATCTGACATCTCCA 181

C_taenia1_gi|62912083|gb|AY940 AATCTTAACAGGATTATTTCTAGCTATACATTATACATCCGATATCACCA 167

M_fossilis1_gi|117959984|gb|DQ AATTCTTACAGGACTATTCCTGGCTATGCACTATACATCTGACATTTCCA 181

A_anguilla1_gi|159461951|gb|EU AATCCTTACAGGACTATTCCTAGCCATACATTATACATCAGACATCTCAA 181

T_thymallus_gi|408833805|gb|JX AATCCTCACCGGGCTATTCTTAGCTATACACTACACCTCTGATATTTCAA 181

S_trutta1_gi|1001096|dbj|D5840 AATTCTTACCGGACTCTTCCTAGCCATACACTACACCTCCGATATCTCAA 181

E_lucius1_gi|325610655|gb|HM17 AATCCTAACCGGCTTATTCCTAGCTATACATTACACCTCTGACATCTCCA 181

L_lota1_gi|77386019|gb|DQ17405 GATTTTAACAGGCCTATTCTTAGCCATACATTACACCTCAGACATCGAAA 181

L_planeri1_gi|261824331|gb|GQ2 AATTATTACAGGACTAATTCTTGCGATACACTATACCGCCAACACTGAAC 193

NM_gi|671723846|gb|KF549990.1 CAGCCTTCTCGTCAGTAACCCACATCTGCCGAGATGTTAACTTTGGCTGA 195

NM_gi|168485493|gb|EU331236.1 CAGCCTTCTCATCAGTAACTCACATCTGCCGAGATGTTAACTTTGGCTGA 231

NF_gi|257220486|gb|GQ444442.1 CAGCCTTCTCATCAGTAGCACACATCTGCCGAGATGTAAACTTTGGCTGA 231

NF_gi|671723852|gb|KF549993.1 CAGCCTTCTCATCAGTAGCACACATCTGCCGAGATGTAAACTTTGGATGG 195

BG_gi|526850801|gb|KF415509.1 CAGCCTTCTCATCCGTAGCACATATCTGCCGAGATGTAAATTTTGGATGA 231

BG_gi|188988554|gb|EU444667.1 CAGCCTTCTCATCCGTAGCACATATCTGCCGAGATGTAAATTTTGGATGA 231

PK_gi|589911366|gb|KC886260.1 CAGCCTTTTCATCTGTGGCACACATCTGCCGAGATGTAAATTTCGGTTGA 207

PM_gi|526851063|gb|KF415640.1 CAGCCTTTTCATCCGTGGCACACATCTGCCGAGATGTTAATTTTGGATGA 231

PM_gi|188988552|gb|EU444666.1 CAGCCTTTTCATCTGTAGCACATATTTGTCGAGATGTAAACTTTGGATGA 231

G_niger_gi|526850949|gb|KF4155 TGGCCTTCTCCTCCGTAGCGCACATCTGCCGAGACGTCAACTTCGGGTGG 231

P_minutus_gi|237638806|gb|FJ52 CTGCTTTTTCATCTGTCGCCCACATTTGCCGTGATGTCAACTTTGGCTGA 231

GC3_gi|20339610|gb|AF386598.1 CAGCCTTTTCATCAGTAGCCCATATCTGCCGAGATGTAAATTATGGATGA 231

PF3_gi|164562184|gb|EU348846.1 CAGCCTTTTCATCAGTCGCCCACATTTGCCGAGACGTAAACTACGGCTGA 231

CG1_gi|27762429|gb|AY116366.1 CAGCCTTCTCATCAGTCGGCCACATCTGCCGAGATGTCAACTACGGATGA 231

GA1_gi|25140372|gb|AY116004.1 CAGCTTTTTCCTCCGTCGGACACATCTGCCGAGATGTAAATTACGGCTGA 231

L_cephalus1_gi|12657413|emb|AJ CCGCATTTTCATCAGTAACCCACATCTGCCGAGATGTAAACTACGGCTGA 231

L_cephalus2_gi|41057843|gb|AY5 CCGCATTTTCATCGGTAACCCACATCTGCCGGGATGTTAACTACGGCTGA 231

L_cepahlus3_gi|269997201|gb|GU CCGCATTCTCATCAGTAGTCCACATTTGCCGGGACGTCAATTACGGCTGA 231

C_nasus1_gi|18460951|gb|AY0264 CCGCATTCTCATCAGTGACCCACATCTGCCGAGATGTCAACTACGGCTGA 231

L_souffia1_gi|29691926|emb|AJ5 CCGCATTCTCATCCGTAACCCACATCTGCCGAGACGTCAACTACGGCTGA 209

R_rutilus1_gi|207113302|gb|FJ0 CCGCGTTTTCATCGGTGACCCACATCTGCCGAGACGTCAACTACGGCTGA 231

B_bjoerkna1_gi|133872335|gb|EF CCGCATTTTCATCAGTAACCCACATCTGCCGAGACGTTAACTACGGCTGA 231

A_brama2_gi|549466030|gb|KF552 CCGCATTTTCATCAGTAACCCACATCTGCCGAGACGTTAACTACGGCTGA 231

B_bjoerkna2_gi|2281719|emb|Y10 CCGCATTTTCATCAGTAACCCACATCTGCCGAGACGTTAACTACGGCTGG 231

L_leuciscus1_gi|307090186|gb|H CCGCATTTTCATCAGTAACTCACATTTGTCGGGACGTTAACTACGGCTGG 231

L_leuciscus3_gi|2281727|emb|Y1 CCGCATTTTCATCAGTAACTCACATTCGTCGGGACGTTAACTACGGCTGG 231

A_alburnus1_gi|386367699|gb|JQ CCGCATTCTCATCAGTCACCCATATTTGCCGGGACGTTAACTACGGCTGG 231

A_alburnus2_gi|85679841|gb|DQ3 CCGCATTCTCATCAGTCACCCATATTTGCCGAGACGTTAACTACGGCTGA 216

L_delineatus1_gi|2281725|emb|Y CCGCATTCTCATCAGTTACTCACATCTGCCGAGACGTCAACTACGGTTGG 231

S_erythrophthalmus1_gi|2140109 CCGCATTTTCATCAGTAACCCATATTTGCCGAGACGTTAACTACGGCTGA 231

A_bipunctatus2_gi|2281713|emb| CCGCATTTTCATCAGTAACCCACATCTGTCGAGACGTCAACTACGGCTGA 231

A_bipunctatus_gi|5832982|gb|AF CCGCATTTTCATCAGTGACCCACATCTGTCGAGACGTTAACTACGGCTGA 231

P_phoxinus1_gi|209968201|gb|EU CCGCATTTTCATCGGTCACCCATATCTGCCGAGACGTTAATTATGGGTGA 231

P_phoxinus2_gi|168203857|gb|EU CCGCATTTTCATCAGTCACCCATATCTGCCGAGACGTTAATTATGGATGA 231

T_tinca1_gi|325169898|gb|HM167 CCGCATTCTCGTCAGTAAACCACATTTGCCGTGATGTAAACTATGGCTGA 231

T_tinca2_gi|444488022|gb|JX974 CCGCATTCTCGTCAGTAAACCACATTTGCCGTGATGTAAACTATGGCTGA 238

G_gobio1_gi|37904500|gb|AY4265 CTGCATTCTCATCAGTTGCCCACATCTGCCGGGACGTAAACTACGGCTGA 231

B_barbus1_gi|462648905|gb|KC46 CCGCATTTTCATCAGTTGTCCATATCTGCCGAGATGTAAACTACGGCTGG 231

C_carpio1_gi|68532783|dbj|AB15 CCGCATTCTCATCTGTTACCCACATCTGCCGAGACGTAAATTACGGCTGA 231

R_amarus1_gi|636792944|dbj|AB3 CCGCATTTTCTTCAGTAAACCATATCTGCCGCGATGTAAATTATGGCTGA 231

B_barbatula2_gi|73671964|gb|DQ CCGCCTTCTCCTCAGTGGCACACATCTGCCGCGACGTCAACTACGGATGA 231

C_taenia1_gi|62912083|gb|AY940 CAGCCTTCTCATCTGTGGCTCACATCTGCCGTGATGTAAATTACGGATGA 217

M_fossilis1_gi|117959984|gb|DQ CTGCCTTCTCATCCGTAGCACACATCTGCCGAGATGTGAACTATGGATGA 231

A_anguilla1_gi|159461951|gb|EU CTGCCTTCTCCTCAGTAGCTCACATCTGCCGAGACGTTAACTATGGATGA 231

T_thymallus_gi|408833805|gb|JX CAGCTTTTTCCTCTGTATGCCACATTTGCCGAGATGTTAGTTACGGATGA 231

S_trutta1_gi|1001096|dbj|D5840 CAGCCTTTTCCTCTGTTTGCCACATTTGCCGAGATGTTAGCTACGGCTGA 231

E_lucius1_gi|325610655|gb|HM17 CAGCCTTCTCATCAGTCTGCCACATCTGCCGGGACGTTAACTACGGCTGA 231

L_lota1_gi|77386019|gb|DQ17405 CAGCCTTTTCATCCGTTGTACACATCTGCCGTGACGTAAACTACGGATGA 231

L_planeri1_gi|261824331|gb|GQ2 TAGCCTTCTCTTCAGTTATACACATTTGTCGTGATGTTAACAACGGATGG 243

NM_gi|671723846|gb|KF549990.1 CTAATCCGCAATATGCACGCCAACGGAGCTTCCTTTTTCTTTATTTGCAT 245

NM_gi|168485493|gb|EU331236.1 CTAATCCGCAATATGCACGCCAACGGAGCTTCCTTTTTCTTTATTTGCAT 281

NF_gi|257220486|gb|GQ444442.1 CTAATCCGCAATATGCACGCCAACGGGGCCTCATTCTTCTTTATCTGCAT 281

NF_gi|671723852|gb|KF549993.1 CTAATCCGCAACATGCACGCCAACGGGGCTTCCTTCTTCTTTATCTGCAT 245

BG_gi|526850801|gb|KF415509.1 CTTATCCGAAATATTCACGCCAACGGGGCCTCCTTCTTCTTTATCTGTAT 281

BG_gi|188988554|gb|EU444667.1 CTTATCCGAAATATTCACGCCAACGGGGCCTCCTTCTTCTTTATCTGTAT 281

PK_gi|589911366|gb|KC886260.1 CTCATCCGAAACATTCATGCCAATGGTGCCTCTTTCTTCTTTATCTGTAT 257

PM_gi|526851063|gb|KF415640.1 CTTATCCGAAATCTTCACGCCAACGGGGCCTCTTTTTTCTTCATTTGTTT 281

PM_gi|188988552|gb|EU444666.1 CTTATTCGAAACCTTCACGCCAATGGAGCCTCCTTCTTTTTCATTTGTTT 281

G_niger_gi|526850949|gb|KF4155 CTTATCCGGAATATGCATGCCAACGGCGCCTCATTCTTTTTTATCTGCAT 281

P_minutus_gi|237638806|gb|FJ52 CTGATCCGGAACATGCACGCCAACGGTGCCTCCTTCTTCTTCATCTGCCT 281

GC3_gi|20339610|gb|AF386598.1 CTTATTCGAAACCTTCATGCCAACGGAGCATCATTCTTTTTCATTTGCAT 281

PF3_gi|164562184|gb|EU348846.1 CTCATTCGAAATATTCATGCCAATGGTGCATCCTTCTTCTTTATTTGCAT 281

CG1_gi|27762429|gb|AY116366.1 CTTATCCGTAACCTCCACGCCAACGGTGCCTCTTTCTTCTTTATTTGCAT 281

GA1_gi|25140372|gb|AY116004.1 CTAATTCGAAACCTCCATGCCAATGGCGCATCCTTTTTCTTCATCTGTAT 281

L_cephalus1_gi|12657413|emb|AJ CTTATTCGAAGTCTGCATGCCAATGGAGCCTCCTTCTTCTTCATCTGTCT 281

L_cephalus2_gi|41057843|gb|AY5 CTTATTCGAAGCCTACATGCCAATGGAGCCTCCTTCTTCTTCATCTGTCT 281

L_cepahlus3_gi|269997201|gb|GU CTTATCCGCAACCTACACGCCAACGGGGCATCATTCTTTTTTATCTGTAT 281

C_nasus1_gi|18460951|gb|AY0264 CTTATCCGAAGCCTGCATGCCAATGGAGCATCCTTTTTCTTCATCTGTCT 281

L_souffia1_gi|29691926|emb|AJ5 CTTATCCGGAACCTACATGCTAACGGGGCATCCTTCTTCTTCATCTGCCT 259

R_rutilus1_gi|207113302|gb|FJ0 CTTATCCGAAACCTACATGCTAATGGAGCATCCTTCTTCTTCATCTGTCT 281

B_bjoerkna1_gi|133872335|gb|EF CTTATTCGAAACTTACATGCTAATGGAGCATCATTCTTCTTTATCTGCCT 281

A_brama2_gi|549466030|gb|KF552 CTTATTCGAAACTTACATGCTAATGGAGCATCATTCTTCTTTATCTGCCT 281

B_bjoerkna2_gi|2281719|emb|Y10 CTCATTCGAAATTTACACGCTAACGGAGCATCATTCTTCTTCATCTGTCT 281

L_leuciscus1_gi|307090186|gb|H CTTATCCGAAACCTGCACGCCAACGGGGCATCATTCTTCTTCATCTGTAT 281

L_leuciscus3_gi|2281727|emb|Y1 CTCATCCGAAACCTGCACGCCAACGGAGCGTCATTCTTCTTCATCTGTAT 281

A_alburnus1_gi|386367699|gb|JQ CTCATTCGAAACCTACATGCCAACGGAGCATCCTTCTTCTTCATCTGCCT 281

A_alburnus2_gi|85679841|gb|DQ3 CTCATTCGAAACCTACATGCCAACGGAGCATCCTTCTTCTTTATCTGCCT 266

L_delineatus1_gi|2281725|emb|Y CTCATTCGAAATCTACATGCTAATGGGGCATCCTTCTTCTTCATCTGTCT 281

S_erythrophthalmus1_gi|2140109 CTTATCCGAAGCCTACATGCCAACGGAGCATCCTTCTTCTTCATCTGTCT 281

A_bipunctatus2_gi|2281713|emb| CTTATCCGAAGCCTTCACGCTAACGGAGCATCTTTTTTCTTCATCTGCCT 281

A_bipunctatus_gi|5832982|gb|AF CTTATCCGAAGCCTTCACGCTAACGGCGCATCTTTTTTCTTCATCTGCCT 281

P_phoxinus1_gi|209968201|gb|EU CTAATTCGAAATATACACGCCAACGGCGCATCATTCTTCTTCATCTGCAT 281

P_phoxinus2_gi|168203857|gb|EU CTAATCCGAAACATGCACGCCAACGGCGCATCATTCTTCTTCATCTGTAT 281

T_tinca1_gi|325169898|gb|HM167 CTTATTCGTAACTTACATGCTAATGGGGCATCATTCTTCTTTATCTGCCT 281

T_tinca2_gi|444488022|gb|JX974 CTTATTCGTAACTTACACGCTAATGGGGCATCATTCTTCTTTATCTGCCT 288

G_gobio1_gi|37904500|gb|AY4265 TTTATTCGTAACGTACACGCCAACGGAGCTTCATTCTTTTTCATCTGCAT 281

B_barbus1_gi|462648905|gb|KC46 CTGATCCGTAACATGCACGCCAACGGAGCATCATTCTTTTTCCTTTGCAT 281

C_carpio1_gi|68532783|dbj|AB15 CTAATCCGTAATGTACACGCCAACGGAGCATCATTCTTCTTCATTTGCAT 281

R_amarus1_gi|636792944|dbj|AB3 CTGATCCGAAATTTACACGCCAACGGCGCATCATTCTTTTTTATCTGCAT 281

B_barbatula2_gi|73671964|gb|DQ CTAATCCGAAACATACACGCTAATGGTGCCTCCTTCTTTTTCATCTGCCT 281

C_taenia1_gi|62912083|gb|AY940 CTTATCCGTAACATTCATGCCAACGGCGCATCATTTTTCTTTATTTGTAT 267

M_fossilis1_gi|117959984|gb|DQ TTTATCCGCAACATTCATGCTAACGGAGCATCACTCTTTTTCATCTGCAT 281

A_anguilla1_gi|159461951|gb|EU CTAATTCGCAACCTACATGCAAATGGAGCCTCATTCTTCTTTATCTGCCT 281

T_thymallus_gi|408833805|gb|JX CTCATCCGAAACATCCACGCTAACGGAGCATCTTTCTTTTTCATTTGCAT 281

S_trutta1_gi|1001096|dbj|D5840 CTCATCCGAAACATTCACGCTAACGGAGCATCTTTCTTCTTTATCTGTAT 281

E_lucius1_gi|325610655|gb|HM17 CTTATCCGAAATATTCACGCTAACGGTGCATCCTTATTCTTCATCTGCAT 281

L_lota1_gi|77386019|gb|DQ17405 CTAATCCGAAATATACACGCCAACGGTGCCTCTTTTTTCTTTATTTGCCT 281

L_planeri1_gi|261824331|gb|GQ2 CTTATACGAAATCTTCATGCCAATGGGGCCTCTATATTCTTTATTTGTAT 293

NM_gi|671723846|gb|KF549990.1 CTACCTTCACATTGGTCGAGGCCTCTATTACGGCTCATACCTATACAAAG 295

NM_gi|168485493|gb|EU331236.1 CTACCTTCACATTGGTCGAGGCCTCTATTACGGCTCATACCTATACAAAG 331

NF_gi|257220486|gb|GQ444442.1 CTACCTTCACATTGGACGAGGCCTCTACTACGGCTCATACCTGTATAAAG 331

NF_gi|671723852|gb|KF549993.1 TTACCTTCACATTGGACGAGGCCTCTATTACGGCTCATACCTGTATAAAG 295

BG_gi|526850801|gb|KF415509.1 TTATATACACATTGGACGAGGCCTTTATTATGGATCCCACCAATACAAAG 331

BG_gi|188988554|gb|EU444667.1 TTATATACACATTGGACGAGGCCTTTATTATGGATCCCACCAATACAAAG 331

PK_gi|589911366|gb|KC886260.1 TTACATGCATATTGGACGAGGACTATATTACGGCTCCCATCAATATAAAG 307

PM_gi|526851063|gb|KF415640.1 GTACCTGCACATCGGACGGGGACTCTACTACGGCTCATACTTATTTAAAG 331

PM_gi|188988552|gb|EU444666.1 GTACCTTCACATTGGGCGAGGCCTCTACTACGGCTCATACTTATATAAAG 331

G_niger_gi|526850949|gb|KF4155 TTACCTTCACATCGGACGAGGCCTCTACTACGGGTCCTACCTTTATAAGG 331

P_minutus_gi|237638806|gb|FJ52 TTACCTCCATGTGGGGCGGGGCCTCTACTACGGCTCCTACCTCTATAAGG 331

GC3_gi|20339610|gb|AF386598.1 TTATATACACATTGGCCGAGGCCTATACTACGGATCCTACCTTTATAAAG 331

PF3_gi|164562184|gb|EU348846.1 CTATATGCACATCGGCCGGGGTTTATATTACGGCTCCTATCTTTACAAAG 331

CG1_gi|27762429|gb|AY116366.1 CTATATGCACATCGGACGGGGCCTTTACTACGGCTCCTACCTCTATAAAG 331

GA1_gi|25140372|gb|AY116004.1 CTATATGCATATCGGCCGAGGACTTTACTATGGCTCTTACCTATACAAAG 331

L_cephalus1_gi|12657413|emb|AJ TTACATGCACATTGCACGAGGACTATACTATGGCTCATACCTATATAAAG 331

L_cephalus2_gi|41057843|gb|AY5 TTATATACACATTGCACGAGGACTATATTATGGCTCATACCTATATAAAG 331

L_cepahlus3_gi|269997201|gb|GU TTATATACACATTGCTCGCGGCCTATACTATGGATCCTACCTTTACAAAG 331

C_nasus1_gi|18460951|gb|AY0264 TTACATGCATATCGCACGGGGCCTATATTATGGGTCATACCTTTATAAAG 331

L_souffia1_gi|29691926|emb|AJ5 TTACATGCACATCGCACGAGGCCTGTATTATGGGTCATACCTTTATAAAG 309

R_rutilus1_gi|207113302|gb|FJ0 TTATATACATATCGCACGAGGCCTATATTACGGGTCATACCTTTATAAGG 331

B_bjoerkna1_gi|133872335|gb|EF TTATATACATATTGCACGAGGCCTATACTACGGGTCATATCTTTACAAAG 331

A_brama2_gi|549466030|gb|KF552 TTATATACATATTGCACGAGGCCTATACTACGGGTCATATCTTTACAAAG 331

B_bjoerkna2_gi|2281719|emb|Y10 TTATATACATATTGCACGAGGCCTATACTACGGATCATATCTTTACAAAG 331

L_leuciscus1_gi|307090186|gb|H TTATATACATATCGCGCGAGGCCTATATTACGGGTCCTATCTTTATAAGG 331

L_leuciscus3_gi|2281727|emb|Y1 TTATATGCATATCGCGCGAGGCCTATATTACGGGTCCTATCTTTATAAGG 331

A_alburnus1_gi|386367699|gb|JQ ATATATGCATATCGCACGAGGTCTATATTACGGCTCATATCTTTATAAAG 331

A_alburnus2_gi|85679841|gb|DQ3 ATATATGCATATTGCACGGGGTCTATATTACGGCTCATACCTTTATAAGG 316

L_delineatus1_gi|2281725|emb|Y GTATATACACATCGCACGAGGTTTATACTACGGCTCATATCTTTATAAAG 331

S_erythrophthalmus1_gi|2140109 TTATATACATATCGCACGAGGACTATATTATGGATCATACCTTTACAAAG 331

A_bipunctatus2_gi|2281713|emb| TTACTTGCACATTGCACGAGGCCTATATTATGGCTCCTACCTTTACAAAG 331

A_bipunctatus_gi|5832982|gb|AF TTATTTACACATTGCACGAGGCCTATATTATGGCTCCTACCTTTACAAAG 331

P_phoxinus1_gi|209968201|gb|EU TTATATGCATATTGCCCGAGGCCTTTATTATGGTTCATATCTTTATAAAG 331

P_phoxinus2_gi|168203857|gb|EU TTATATGCATATCGCCCGTGGTCTTTATTATGGTTCATATCTTTATAAAG 331

T_tinca1_gi|325169898|gb|HM167 TTATATACATATCGCCCGAGGATTATATTACGGATCATACCTTTACAAAG 331

T_tinca2_gi|444488022|gb|JX974 TTATATACATATCGCCCGAGGATTATATTACGGATCATACCTTTACAAAG 338

G_gobio1_gi|37904500|gb|AY4265 TTACATGCACGTAGCCCGAGGCCTCTATTACGGATCCTACCCCTACAAAG 331

B_barbus1_gi|462648905|gb|KC46 CTACATACACATTGCCCGAGGACTGTACTACGGCTCTTACCTCTACAAGG 331

C_carpio1_gi|68532783|dbj|AB15 TTACATACACATCGCCCGAGGCCTATACTACGGATCATACCTTTACAAAG 331

R_amarus1_gi|636792944|dbj|AB3 CTACATACACATCGCCCGCGGTCTGTACTACGGGTCCTATCTCTACAAAG 331

B_barbatula2_gi|73671964|gb|DQ TTACATACACATCGCCCGAGGACTATATTATGGGTCCTACCTATACAAAG 331

C_taenia1_gi|62912083|gb|AY940 TTATATTCACATCGCCCGAGGACTATATTATGGATCTTATCTCTACAAAG 317

M_fossilis1_gi|117959984|gb|DQ CTATATTCATATTGCCCGAGGACTATACTATGGGTCATATCTCTATAAAG 331

A_anguilla1_gi|159461951|gb|EU ATACCTCCACATTGCCCGAGGACTTTACTACGGCTCATACCTTTACATAG 331

T_thymallus_gi|408833805|gb|JX TTATATACACATTGCCCGAGGACTTTACTACGGCTCATACCTATATAAAG 331

S_trutta1_gi|1001096|dbj|D5840 TTATATACATATCGCCCGAGGACTCTACTATGGTTCCTACCTATATAAAG 331

E_lucius1_gi|325610655|gb|HM17 CTACATACACATCGCCCGAGGCCTTTATTATGGCTCTTACTTATACAAAG 331

L_lota1_gi|77386019|gb|DQ17405 CTATCTCCACATTGCCCGAGGCTTATATTACGGCTCATACCTATTTATTG 331

L_planeri1_gi|261824331|gb|GQ2 TTATGCTCACATCGGGCGAGGGATCTACTACGGCTCTTATTTATATAAAG 343

NM_gi|671723846|gb|KF549990.1 AGACCTGAAACATCGGAGTAGTACTACTACTCCTAGTTATGATAACTGCT 345

NM_gi|168485493|gb|EU331236.1 AGACCTGAAACATCGGAGTAGTACTACTACTCCTAGTTATAATAACTGCT 381

NF_gi|257220486|gb|GQ444442.1 AGACTTGAAATATTGGGGTTATTCTTTTACTCCTGGTTATAATAACTGCT 381

NF_gi|671723852|gb|KF549993.1 AAACCTGAAATATCGGTGTAATTCTTCTCCTCCTGGTTATAATGACTGCT 345

BG_gi|526850801|gb|KF415509.1 AAACCTGAAATATTGGAGTCGTCCTACTGCTCCTAGTAATAATAACCGCC 381

BG_gi|188988554|gb|EU444667.1 AAACCTGAAATATTGGAGTCGTCCTACTGCTCCTAGTAATAATAACCGCC 381

PK_gi|589911366|gb|KC886260.1 AAACTTGAAATATTGGAGTCGTACTTCTGCTCCTAGTGATAATAACCGCC 357

PM_gi|526851063|gb|KF415640.1 AAACATGAAATATTGGAGTAGTCCTATTTCTCCTAGTAATAATAACCGCC 381

PM_gi|188988552|gb|EU444666.1 AGACATGATTTATTGGGGTAGTACTATTCCTCCTAGTTATAATAACCGCA 381

G_niger_gi|526850949|gb|KF4155 AAACCTGGACTATTGGAGTGGTACTGCTCCTTCTTGTCATGATGACCGCC 381

P_minutus_gi|237638806|gb|FJ52 AAACGTGAAATATCGGCGTCGTTCTGCTTCTCCTGGTAATGATGACTGCC 381

GC3_gi|20339610|gb|AF386598.1 AAACATGAAACATCGGAGTTGTCCTATTGCTCCTAGTAATAATGACCGCC 381

PF3_gi|164562184|gb|EU348846.1 AAACATGAAACATTGGAGTTGTTCTCCTTCTCCTAGTAATAATAACCGCC 381

CG1_gi|27762429|gb|AY116366.1 AAACTTGAAACATTGGGGTCGTCCTCCTGCTCCTTGTAATAATAACCGCT 381

GA1_gi|25140372|gb|AY116004.1 AAACCTGAAACATCGGAGTAATTCTTCTACTTTTAGTCATGATAACAGCC 381

L_cephalus1_gi|12657413|emb|AJ AAACCTGAAACATTGGTGTAGTCCTGTTCCTCCTAGTTATAATAACAGCC 381

L_cephalus2_gi|41057843|gb|AY5 AAACCTGAAACATTGGTGTAGTCCTGTTTCTCCTAGTTATAATGACAGCC 381

L_cepahlus3_gi|269997201|gb|GU AAACCTGAAATATTGGAGTAGTCCTACTCCTATTAGTTATAATAACGGCC 381

C_nasus1_gi|18460951|gb|AY0264 AAACCTGAAACATCGGTGTAGTCCTATTCCTTCTGGTTATGATAACGGCC 381

L_souffia1_gi|29691926|emb|AJ5 AGACCTGAAGCATTGGTGTGGTTCTATTCCTTCTGGTTATGATGACAGCC 359

R_rutilus1_gi|207113302|gb|FJ0 AAACCTGAAACATTGGTGTGGTTCTATTCCTCCTGGTTATAATGACAGCC 381

B_bjoerkna1_gi|133872335|gb|EF AAACCTGAAATATTGGCGTAGTCCTATTTCTTCTAGTTATAATAACAGCC 381

A_brama2_gi|549466030|gb|KF552 AAACCTGAAATATTGGCGTAGTCCTATTTCTTCTAGTTATAATAACAGCC 381

B_bjoerkna2_gi|2281719|emb|Y10 AAACCTGAAATATTGGTGTAGTCCTATTTCTTCTAGTTATAATGACAGCC 381

L_leuciscus1_gi|307090186|gb|H AGACCTGAAATATCGGTGTAGTACTACTTCTTCTAGTCATAGCAACCGCC 381

L_leuciscus3_gi|2281727|emb|Y1 AGACCTGAAATATTGGTGTGGTACTACTTCTTCTAGTCATAGCAACCGCC 381

A_alburnus1_gi|386367699|gb|JQ AGACCTGAAACATTGGGGTAGTACTATTTCTTCTGGTTATGATAACAGCC 381

A_alburnus2_gi|85679841|gb|DQ3 AGACCTGAAACATCGGGGTAGTACTATTTCTTCTGGTTATGATAACAGCC 366

L_delineatus1_gi|2281725|emb|Y AGACCTGAAATATTGGAGTAGTACTATTTCTTCTGGTTATGATGACAGCC 381

S_erythrophthalmus1_gi|2140109 AAACCTGAAATATTGGTGTAGTCCTATTCCTTTTGGTTATGATGACGGCC 381

A_bipunctatus2_gi|2281713|emb| AAACCTGAAACATCGGCGTAGTCCTATTCCTTCTAGTAATAATGACAGCC 381

A_bipunctatus_gi|5832982|gb|AF AAACCTGAAACATCGGCGTAGTCCTATTTCTTTTAGTGATAATAACAGCC 381

P_phoxinus1_gi|209968201|gb|EU AAACCTGAAACATTGGAGTAGTACTACTCCTCCTGGTAATAATAACAGCC 381

P_phoxinus2_gi|168203857|gb|EU AAACCTGAAATATCGGGGTAGTTCTCCTCCTCCTGGTTATAATAACGGCC 381

T_tinca1_gi|325169898|gb|HM167 AAACCTGAAATATTGGAGTAGTTCTTTTTCTATTAGTAATAATAACAGCC 381

T_tinca2_gi|444488022|gb|JX974 AGACCTGAAATATTGGAGTAGTTCTTTTTCTATTAGTAATAATAACAGCC 388

G_gobio1_gi|37904500|gb|AY4265 AAACCTGAAACATTGGAGTGGTTCTGCTGCTGCTAGTTATAATAACGGCC 381

B_barbus1_gi|462648905|gb|KC46 AAACCTGAAACATCGGCGTAATTCTCCTACTACTAGTCATAGCGACAGCC 381

C_carpio1_gi|68532783|dbj|AB15 AAACCTGAAACATTGGTGTAGTCCTTCTACTACTAGTCATGATAACAGCC 381

R_amarus1_gi|636792944|dbj|AB3 AGACCTGAAATATTGGTGTTATCCTCTTTCTGCTCGTCATAATGACCGCT 381

B_barbatula2_gi|73671964|gb|DQ AAACCTGAAATATTGGGGTAGTCATTTTCCTATTGGTAATAATGACAGCT 381

C_taenia1_gi|62912083|gb|AY940 AGACTTGAAATATTGGAGTAGTTCTCCTTCTACTCGTTATAATAACAGCA 367

M_fossilis1_gi|117959984|gb|DQ AGACCTGAAATATTGGAGTTATTCTCCTCCTATTAGTTATAATAACAGCA 381

A_anguilla1_gi|159461951|gb|EU AAACATGAAACATTGGAGTTGTATTATTCCTATTAGTAATAATAACAGCA 381

T_thymallus_gi|408833805|gb|JX AAACCTGAAACATCGGAGTTGTACTCCTTCTACTAACTATAATGACAGCC 381

S_trutta1_gi|1001096|dbj|D5840 AAACCTGAAATATCGGAGTCGTACTGCTACTTCTCACTATAATAACCGCC 381

E_lucius1_gi|325610655|gb|HM17 AAACCTGAAATATCGGAGTTATCCTCCTCCTCCTAACAATAATAACCGCC 381

L_lota1_gi|77386019|gb|DQ17405 AAACATGAAATATCGGGGTTATTTTATTCCTGTTAGTAATAGTAACCTCT 381

L_planeri1_gi|261824331|gb|GQ2 AAACATGAAACGTTGGAGTCATCTTATTTGCATTAACTGCAGCTACTGCT 393

NM_gi|671723846|gb|KF549990.1 TTTGTGGGCTACGTCCTCCCATGAGGACAAATGTCCTTTTGAGGGGCCAC 395

NM_gi|168485493|gb|EU331236.1 TTTGTGGGCTACGTCCTCCCATGAGGACAAATGTCCTTTTGAGGGGCTAC 431

NF_gi|257220486|gb|GQ444442.1 TTTGTAGGCTACGTCCTCCCATGAGGACAAATATCCTTTTGAGGTGCCAC 431

NF_gi|671723852|gb|KF549993.1 TTTGTAGGTTACGTCCTCCCATGGGGACAAATATCCTTTTGAGGTGCCAC 395

BG_gi|526850801|gb|KF415509.1 TTCGTGGGTTACGTCTTACCATGAGGGCAAATATCTTTCTGAGGTGCAAC 431

BG_gi|188988554|gb|EU444667.1 TTCGTGGGCTACGTCTTACCATGAGGGCAAATATCTTTCTGAGGTGCAAC 431

PK_gi|589911366|gb|KC886260.1 TTCGTGGGCTACGTCCTGCCATGAGGACAAATATCCTTCTGAGGTGCCAC 407

PM_gi|526851063|gb|KF415640.1 TTCGTGGGCTATGTACTTCCATGAGGACAAATATCTTTCTGAGGAGCAAC 431

PM_gi|188988552|gb|EU444666.1 TTCGTGGGCTATGTTCTTCCATGGGGGCAAATATCCTTCTGAGGGGCAAC 431

G_niger_gi|526850949|gb|KF4155 TTCGTCGGGTACGTCCTCCCCTGAGGACAGATATCTTTCTGGGGTGCAAC 431

P_minutus_gi|237638806|gb|FJ52 TTCGTCGGCTACGTCCTCCCCTGGGGACAGATGTCGTTTTGGGGGGCAAC 431

GC3_gi|20339610|gb|AF386598.1 TTTGTAGGATACGTTTTACCCTGAGGACAGATATCTTTCTGAGGAGCAAC 431

PF3_gi|164562184|gb|EU348846.1 TTTGTTGGGTACGTCCTGCCCTGAGGACAAATATCTTTCTGGGGTGCGAC 431

CG1_gi|27762429|gb|AY116366.1 TTCGTAGGGTACGTCCTCCCCTGAGGACAGATGTCATTCTGAGGGGCCAC 431

GA1_gi|25140372|gb|AY116004.1 TTCGTTGGTTATGTCCTTCCATGAGGACAAATATCTTTCTGAGGAGCTAC 431

L_cephalus1_gi|12657413|emb|AJ TTTGTCGGCTACGTACTTCCGTGGGGACAGATGTCTTTTTGAGGCGCCAC 431

L_cephalus2_gi|41057843|gb|AY5 TTTGTCGGCTACGTACTTCCGTGAGGACAAATATCCTTTTGGGGTGCCAC 431

L_cepahlus3_gi|269997201|gb|GU TTCGTTGGCTACGTCCTCCCATGAGGACAAATATCTTTTTGAGGCGCTAC 431

C_nasus1_gi|18460951|gb|AY0264 TTTGTAGGCTACGTTCTTCCGTGGGGACAAATATCCTTTTGAGGTGCTAC 431

L_souffia1_gi|29691926|emb|AJ5 TTCGTCGGCTACGTTCTTCCATGAGGACAAATATCCTTTTGAGGTGCTAC 409

R_rutilus1_gi|207113302|gb|FJ0 TTCGTTGGCTACGTACTACCATGGGGGCAAATATCCTTCTGAGGCGCCAC 431

B_bjoerkna1_gi|133872335|gb|EF TTCGTCGGCTACGTACTTCCATGAGGACAAATGTCCTTTTGAGGTGCCAC 431

A_brama2_gi|549466030|gb|KF552 TTCGTCGGCTACGTACTTCCATGAGGACAAATGTCCTTTTGAGGTGCCAC 431

B_bjoerkna2_gi|2281719|emb|Y10 TTCGTCGGCTATGTACTTCCATGAGGGCAGATATCTTTTTGAGGTGCCAC 431

L_leuciscus1_gi|307090186|gb|H TTCGTGGGCTATGTGCTTCCGTGGGGCCAAATATCTTTTTGAGGTGCTAC 431

L_leuciscus3_gi|2281727|emb|Y1 TTCGTGGGCTATGTACTTCCCTGGGGCCAAATATCTTTTTGAGGTGCTAC 431

A_alburnus1_gi|386367699|gb|JQ TTCGTGGGCTATGTACTCCCATGAGGACAAATATCCTTTTGAGGCGCTAC 431

A_alburnus2_gi|85679841|gb|DQ3 TTCGTGGGCTATGTACTCCCATGAGGACAAATGTCCTTTTGAGGTGCTAC 416

L_delineatus1_gi|2281725|emb|Y TTCGTGGGCTATGTATTACCATGAGGACAAATGTCCTTCTGGGGCGCTAC 431

S_erythrophthalmus1_gi|2140109 TTCGTCGGTTACGTACTTCCATGAGGGCAAATGTCCTTTTGAGGTGCTAC 431

A_bipunctatus2_gi|2281713|emb| TTCGTCGGCTACGTACTTCCATGGGGACAAATATCATTTTGGGGCGCCAC 431

A_bipunctatus_gi|5832982|gb|AF TTCGTCGGCTACGTGCTCCCATGGGGACAAATATCATTTTGAGGTGCCAC 431

P_phoxinus1_gi|209968201|gb|EU TTTGTGGGCTACGTACTTCCATGAGGCCAAATGTCTTTTTGAGGCGCCAC 431

P_phoxinus2_gi|168203857|gb|EU TTCGTTGGCTACGTCCTTCCCTGAGGCCAAATGTCTTTTTGAGGTGCCAC 431

T_tinca1_gi|325169898|gb|HM167 TTTGTTGGCTACGTCCTGCCATGAGGACAAATATCCTTTTGAGGCGCAAC 431

T_tinca2_gi|444488022|gb|JX974 TTTGTTGGCTACGTCCTGCCATGAGGACAAATATCCTTTTGAGGCGCAAC 438

G_gobio1_gi|37904500|gb|AY4265 TTCGTTGGCTATGTTCTCCCATGGGGTCAAATATCCTTCTGAGGTGCCAC 431

B_barbus1_gi|462648905|gb|KC46 TTCGTTGGCTACGTACTTCCATGAGGCCAAATATCTTTCTGAGGCGCTAC 431

C_carpio1_gi|68532783|dbj|AB15 TTCGTTGGCTATGTTCTTCCATGAGGACAAATATCCTTTTGAGGCGCCAC 431

R_amarus1_gi|636792944|dbj|AB3 TTCGTAGGCTACGTTTTACCCTGAGGTCAAATATCATTCTGGGGCGCCAC 431

B_barbatula2_gi|73671964|gb|DQ TTTGTAGGCTATGTATTACCATGGGGTCAAATATCTTTTTGAGGTGCCAC 431

C_taenia1_gi|62912083|gb|AY940 TTCGTTGGCTATGTCCTTCCATGAGGTCAAATATCCTTTTGAGGGGCCAC 417

M_fossilis1_gi|117959984|gb|DQ TTTGTAGGCTATGTACTCCCATGGGGGCAAATATCCTTCTGGGGTGCCAC 431

A_anguilla1_gi|159461951|gb|EU TTCGTAGGATATGTGCTTCCATGAGGACAGATATCATTCTGAGGTGCTAC 431

T_thymallus_gi|408833805|gb|JX TTCGTAGGCTATGTTCTACCATGAGGACAAATATCTTTCTGAGGAGCAAC 431

S_trutta1_gi|1001096|dbj|D5840 TTCGTGGGCTACGTTCTTCCATGAGGGCAGATGTCCTTCTGAGGAGCCAC 431

E_lucius1_gi|325610655|gb|HM17 TTCGTTGGCTATGTTCTGCCCTGAGGACAAATATCTTTTTGAGGCGCAAC 431

L_lota1_gi|77386019|gb|DQ17405 TTTGTAGGCTATGTTCTTCCCTGAGGACAAATATCCTTCTGAGGAGCAAC 431

L_planeri1_gi|261824331|gb|GQ2 TTCGTTGGTTATGTACTACCATGAGGGCAAATATCCTTCTGAGGGGCAAC 443

NM_gi|671723846|gb|KF549990.1 CGTGATCACCAACCTCCTCTCTGCCGTCCCTTACGTGGGCGGAAACCTAG 445

NM_gi|168485493|gb|EU331236.1 CGTGATCACCAACCTCCTCTCTGCCGTCCCTTACGTGGGCGGAAACCTAG 481

NF_gi|257220486|gb|GQ444442.1 TGTAATTACTAACCTTTTATCTGCTGTCCCCTACGTAGGAGGGAGCCTAG 481

NF_gi|671723852|gb|KF549993.1 CGTAATTACTAATCTTCTATCTGCCGTCCCTTACGTAGGAGGAAGCCTAG 445

BG_gi|526850801|gb|KF415509.1 CGTAATTACAAATCTTCTCTCTGCTGTTCCCTACATCGGGGGAGACTTAG 481

BG_gi|188988554|gb|EU444667.1 CGTAATTACAAACCTTCTCTCTGCTGTTCCCTACATCGGGGGAAACTTAG 481

PK_gi|589911366|gb|KC886260.1 CGTAATTACAAACCTTCTCTCTGCTGTTCCCTACATTGGAGGAGACTTAG 457

PM_gi|526851063|gb|KF415640.1 CGTAATTACAAATCTCCTCTCTGCTATCCCTTACATTGGCGGGGATTTAG 481

PM_gi|188988552|gb|EU444666.1 CGTAATTACAAACCTCCTCTCTGCTATCCCTTATGTAGGAACTGATCTAG 481

G_niger_gi|526850949|gb|KF4155 CGTGATTACGAACCTACTTTCCGCCGTGCCCTACGTCGGAGGAACCCTTG 481

P_minutus_gi|237638806|gb|FJ52 TGTCATTACCAACCTCCTCTCCGCGGTCCCCTACGTGGGCAATAGCCTTG 481

GC3_gi|20339610|gb|AF386598.1 TGTCATCACCAACCTCCTGTCCGCAGTACCTTATATTGGCAACACCCTAG 481

PF3_gi|164562184|gb|EU348846.1 CGTCATTACCAACCTTCTGTCAGCAGTCCCTTACATTGGCAATACCCTTG 481

CG1_gi|27762429|gb|AY116366.1 TGTCATCACAAACCTTCTTTCTGCAGTTCCTTACATCGGCAACGCCCTGG 481

GA1_gi|25140372|gb|AY116004.1 AGTTATTACCAACCTACTTTCAGCCGTCCCATACGTTGGCAACTCATTAG 481

L_cephalus1_gi|12657413|emb|AJ GGTAATTACGAACCTGCTCTCAGCGGTCCCTTACATAGGGGACATGCTTG 481

L_cephalus2_gi|41057843|gb|AY5 AGTGATTACAAACCTCCTCTCAGCAGTCCCTTACATGGGGGACACTCTTG 481

L_cepahlus3_gi|269997201|gb|GU CGTAATTACAAACCTACTATCAGCAGTCCCCTACATAGGAGATACTCTCG 481

C_nasus1_gi|18460951|gb|AY0264 CGTAATTACAAACCTCCTCTCTGCCGTCCCCTACATGGGGGATACCCTTG 481

L_souffia1_gi|29691926|emb|AJ5 CGTAATTACGAACCTCCTCTCAGCAGTCCCTTACATGGGAGATACCCTTG 459

R_rutilus1_gi|207113302|gb|FJ0 CGTAATCACAAACCTCCTCTCAGCGGTCCCTTACATGGGAGATACCCTTG 481

B_bjoerkna1_gi|133872335|gb|EF CGTAATTACAAATCTCCTCTCAGCAGTCCCTTATATAGGAGACACTCTTG 481

A_brama2_gi|549466030|gb|KF552 CGTAATTACAAATCTCCTCTCAGCAGTCCCTTATATAGGAGACACTCTTG 481

B_bjoerkna2_gi|2281719|emb|Y10 CGTAATTACAAACCTCCTCTCAGCAGTCCCTTATATAGGCGACACCCTCG 481

L_leuciscus1_gi|307090186|gb|H TGTTATTACAAATCTCCTATCAGCAGTCCCTTATATGGGCGACACCCTTG 481

L_leuciscus3_gi|2281727|emb|Y1 TGTTATTACAAATCTCCTATCAGCAGTCCCTTATATGGGTGACACCCTTG 481

A_alburnus1_gi|386367699|gb|JQ CGTAATCACGAACCTCCTCTCAGCAGTTCCCTACATGGGAGATACCCTTG 481

A_alburnus2_gi|85679841|gb|DQ3 CGTAATCACAAATCTCCTCTCAGCAGTTCCCTACATGGGAGACACCCTCG 466

L_delineatus1_gi|2281725|emb|Y CGTGATTACAAACCTCCTCTCAGCGGTCCCCTACATAGGAGACACCCTTG 481

S_erythrophthalmus1_gi|2140109 CGTAATTACAAACCTCCTCTCAGCAGTCCCCTACATAGGAGATACCCTTG 481

A_bipunctatus2_gi|2281713|emb| CGTCATCACGAATCTCCTTTCAGCAGTACCTTACATGGGAGACATACTTG 481

A_bipunctatus_gi|5832982|gb|AF CGTCATTACAAATCTCCTTTCAGCAGTACCCTACATGGGAGACATACTTG 481

P_phoxinus1_gi|209968201|gb|EU AGTAATTACTAACTTGTTATCAGCAGTCCCTTACATAGGAGACATACTGG 481

P_phoxinus2_gi|168203857|gb|EU AGTAATTACAAACTTATTATCAGCAGTCCCCTACATGGGGGACATACTGG 481

T_tinca1_gi|325169898|gb|HM167 AGTAATTACTAACCTACTATCAGCAGTTCCCTACATAGGAGATGCTTTAG 481

T_tinca2_gi|444488022|gb|JX974 AGTAATTACTAACCTACTATCAGCAGTTCCCTACATAGGAGATGCTTTAG 488

G_gobio1_gi|37904500|gb|AY4265 AGTCATTACTAACCTTTTATCAGCAGTCCCTTATATGGGGGACACCCTTG 481

B_barbus1_gi|462648905|gb|KC46 AGTAATTACAAATCTCCTCTCCGCCGTACCATATATAGGAGACATACTAG 481

C_carpio1_gi|68532783|dbj|AB15 AGTAATCACAAACCTCCTATCTGCCGTACCATACATGGGAGACATGTTAG 481

R_amarus1_gi|636792944|dbj|AB3 AGTAATTACCAATCTACTCTCAGCGGTCCCTTATATAGGGGACGCCCTGG 481

B_barbatula2_gi|73671964|gb|DQ AGTCATCACCAACCTATTGTCAGCCGTACCTTATGTAGGAGATGTTTTAG 481

C_taenia1_gi|62912083|gb|AY940 AGTAATTACCAATCTCCTATCAGCAGTCCCTTATGTAGGAAATGCCCTAG 467

M_fossilis1_gi|117959984|gb|DQ AGTAATCACCAACCTTCTATCTGCAGTTCCCTACATAGGAAACACCCTAG 481

A_anguilla1_gi|159461951|gb|EU AGTAATTACCAACCTACTATCTGCCGTCCCATATGTGGGGAACTCCCTAG 481

T_thymallus_gi|408833805|gb|JX CGTAATTACGAACCTCTTATCAGCCGTCCCTTATGTGGGAGGTGCCCTAG 481

S_trutta1_gi|1001096|dbj|D5840 TGTAATTACAAACCTTCTCTCCGCTGTCCCATACGTTGGAGGCGCCCTTG 481

E_lucius1_gi|325610655|gb|HM17 AGTAATTACTAACCTATTATCAGCTGTTCCCTACATTGGGAATGACCTGG 481

L_lota1_gi|77386019|gb|DQ17405 CGTAATTACAAACCTAATATCTACTGTACCCTACGTAGGAAATACCCTAG 481

L_planeri1_gi|261824331|gb|GQ2 CGTTATTACAAATTTAATTTCAGCAGTACCTTATGTAGGAGATGATATTG 493

NM_gi|671723846|gb|KF549990.1 TACAATGAATTTGGGGGGGATTTTCAGTCGATAACGCAACCCTAACCCGA 495

NM_gi|168485493|gb|EU331236.1 TACAATGAATTTGGGGTGGATTTTCAGTCGATAACGCAACCCTGACCCGA 531

NF_gi|257220486|gb|GQ444442.1 TACAATGGATCTGAGGCGGGTTTTCAGTTGACAACGCAACCCTGACACGA 531

NF_gi|671723852|gb|KF549993.1 TACAATGAATCTGAGGGGGGTTTTCGGTTGATAACGCAACCCTAACACGA 495

BG_gi|526850801|gb|KF415509.1 TGCAATGAATTTGAGGGGGCTTCTCAGTCGACAACGCAACCCTAACACGA 531

BG_gi|188988554|gb|EU444667.1 TGCAATGAATTTGAGGGGGCTTCTCAGTCGACAACGCAACCCTAACACGA 531

PK_gi|589911366|gb|KC886260.1 TACAATGAATTTGAGGGGGCTTCTCAGTTGATAACGCAACCCTAACACGA 507

PM_gi|526851063|gb|KF415640.1 TACAATGAATCTGGGGAGGCTTTTCAGTTGATAATGCAACCCTAACACGA 531

PM_gi|188988552|gb|EU444666.1 TACAGTGAATTTGAGGGGGCTTCTCAGTTGATAACGCAACCCTCACACGA 531

G_niger_gi|526850949|gb|KF4155 TGCAATGAATCTGGGGCGGCTTCTCAGTCGACAACGCAACGCTAACTCGA 531

P_minutus_gi|237638806|gb|FJ52 TGCAGTGAATCTGGGGGGGCTTCTCGGTTGACAATGCTACCCTCACGCGC 531

GC3_gi|20339610|gb|AF386598.1 TACAATGAATCTGGGGAGGCTTCTCAGTTGATAATGCCACCCTCACCCGA 531

PF3_gi|164562184|gb|EU348846.1 TTCAATGGATCTGAGGCGGCTTCTCAGTAGATAACGCCACCCTCACTCGA 531

CG1_gi|27762429|gb|AY116366.1 TCCAATGAATCTGAGGAGGCTTCTCAGTAGACAACGCCACACTCACACGA 531

GA1_gi|25140372|gb|AY116004.1 TTCAATGAATTTGAGGGGGCTTTTCCGTTGACAACGCCACCTTAACACGT 531

L_cephalus1_gi|12657413|emb|AJ TTCAATGAATCTGAGGTGGTTTCTCAGTAGATAATGCAACCCTTACCCGA 531

L_cephalus2_gi|41057843|gb|AY5 TTCAATGAATCTGAGGCGGTTTCTCCGTAGATAACGCAACCCTCACCCGA 531

L_cepahlus3_gi|269997201|gb|GU TCCAATGAATTTGAGGTGGCTTCTCAGTAGATAATGCAACACTAACACGA 531

C_nasus1_gi|18460951|gb|AY0264 TTCAATGAATCTGAGGCGGCTTCTCAGTAGACAATGCGACTCTCACACGG 531

L_souffia1_gi|29691926|emb|AJ5 TTCAGTGAATCTGAGGCGGTTTCTCAGTAGATAATGCAACTCTTACGCGG 509

R_rutilus1_gi|207113302|gb|FJ0 TTCAGTGAATCTGGGGAGGTTTCTCAGTAGATAACGCAACCCTTACACGG 531

B_bjoerkna1_gi|133872335|gb|EF TCCAATGAATCTGAGGCGGCTTTTCAGTAGACAACGCAACTCTCACACGA 531

A_brama2_gi|549466030|gb|KF552 TCCAATGAATCTGAGGCGGCTTTTCAGTAGACAACGCAACTCTCACACGA 531

B_bjoerkna2_gi|2281719|emb|Y10 TCCAATGAATCTGAGGCGGTTTCTCAGTAGATAACGCAACTCTCACACGA 531

L_leuciscus1_gi|307090186|gb|H TTCAATGAATTTGAGGCGGCTTCTCAGTAGACAACGCGACCCTCACCCGG 531

L_leuciscus3_gi|2281727|emb|Y1 TTCAATGAATTTGGGGCGGCTTCTCGGTAGACAACGCGACCCTCACCCGG 531

A_alburnus1_gi|386367699|gb|JQ TTCAATGAATTTGGGGCGGTTTCTCAGTAGATAACGCGACTCTTACGCGA 531

A_alburnus2_gi|85679841|gb|DQ3 TTCAATGAATTTGAGGCGGTTTCTCAGTAGATAACGCGACTCTTACGCGA 516

L_delineatus1_gi|2281725|emb|Y TTCAATGAATCTGGGGTGGTTTCTCAGTAGATAATGCAACCCTCACACGA 531

S_erythrophthalmus1_gi|2140109 TTCAGTGAATCTGAGGCGGTTTCTCAGTAGACAACGCGACCCTAACACGA 531

A_bipunctatus2_gi|2281713|emb| TTCAATGAATCTGGGGCGGCTTCTCAGTAGATAACGCAACCCTCACACGA 531

A_bipunctatus_gi|5832982|gb|AF TTCAATGAATCTGAGGCGGCTTCTCAGTAGACAACGCAACCCTCACACGA 531

P_phoxinus1_gi|209968201|gb|EU TTCAATGAATCTGGGGTGGCTTCTCAGTAGATAATGCTACCCTCACGCGA 531

P_phoxinus2_gi|168203857|gb|EU TTCAATGAATCTGGGGCGGCTTCTCAGTAGATAATGCAACCCTCACGCGA 531

T_tinca1_gi|325169898|gb|HM167 TTCAATGAATCTGAGGGGGCTTCTCAGTAGACAATGCAACACTTACACGA 531

T_tinca2_gi|444488022|gb|JX974 TTCAATGAATCTGAGGTGGCTTCTCAGTAGACAATGCAACACTTACGCGA 538

G_gobio1_gi|37904500|gb|AY4265 TCCAATGAATTTGAGGCGGGTTTTCAGTTGATAACGCAACACTAACACGA 531

B_barbus1_gi|462648905|gb|KC46 TCCAATGAATCTGAGGCGGATTTTCGGTAGATAACGCAACACTGACACGA 531

C_carpio1_gi|68532783|dbj|AB15 TCCAATGAATCTGAGGTGGGTTCTCAGTAGACAATGCAACACTAACACGA 531

R_amarus1_gi|636792944|dbj|AB3 TTCAATGAATTTGAGGCGGGTTCTCAGTAGACAACGCAACACTAACTCGA 531

B_barbatula2_gi|73671964|gb|DQ TACAATGGATTTGAGGGGGCTTCTCGGTGGACAATGCAACCCTAACACGA 531

C_taenia1_gi|62912083|gb|AY940 TCCAGTGAATTTGAGGTGGATTCTCGGTGGATAATGCTACACTAACACGA 517

M_fossilis1_gi|117959984|gb|DQ TCCAATGAATTTGGGGGGGCTTCTCAGTAGATAATGCCACACTAACACGA 531

A_anguilla1_gi|159461951|gb|EU TCCAATGAATCTGAGGGGGATTCTCAGTTGACAACGCCACATTAACCCGA 531

T_thymallus_gi|408833805|gb|JX TACAATGAATTTGAGGGGGGTTCTCCGTAGATAACGCCACTTTGACACGG 531

S_trutta1_gi|1001096|dbj|D5840 TACAATGAATTTGAGGCGGATTCTCTGTCGACAACGCCACCCTAACACGA 531

E_lucius1_gi|325610655|gb|HM17 TCCAATGAATCTGAGGCGGCTTCTCCGTCGATAACGCAACCCTTACACGA 531

L_lota1_gi|77386019|gb|DQ17405 TTCAATGAATTTGAGGAGGCTTCTCAGTTGACAACGCCACCCTTACCCGC 531

L_planeri1_gi|261824331|gb|GQ2 TAGTATGATTATGAGGCGGCTTCTCAGTATCAAACGCCACATTAACCCGG 543

NM_gi|671723846|gb|KF549990.1 TTTTTCGCCTTCCATTTTATTCTGCCCTTTATTATTCTAGCTGCAACGTT 545

NM_gi|168485493|gb|EU331236.1 TTTTTCGCCTTCCATTTTATTCTGCCCTTTATTATTCTAGCTGCAACATT 581

NF_gi|257220486|gb|GQ444442.1 TTTTTTGCCTTCCACTTCCTACTACCCTTCGTTATTCTAGCCGCCACGCT 581

NF_gi|671723852|gb|KF549993.1 TTTTTCGCCTTCCACTTCCTATTGCCCTTTGTAATCCTAGCCGCCACGCT 545

BG_gi|526850801|gb|KF415509.1 TTTTTTGCCTTTCACTTCCTTTTCCCCTTTGTGATTTTAGCCGCCACCCT 581

BG_gi|188988554|gb|EU444667.1 TTTTTTGCCTTTCACTTCCTTTTCCCCTTTGTGATTTTAGCCGCCACCCT 581

PK_gi|589911366|gb|KC886260.1 TTTTTTGCCTTTCACTTCCTTCTACCATTTGTCATTTTGGCTGCCACTCT 557

PM_gi|526851063|gb|KF415640.1 TTTTTTGCCTTTCACTTTCTGCTACCCTTCGTTATCTTGGCCGTCACCCT 581

PM_gi|188988552|gb|EU444666.1 TTCTTCGCCTTTCATTTCCTACTGCCATTTGTTGTCTTGGCCATCACCTT 581

G_niger_gi|526850949|gb|KF4155 TTCTTTGCCTTCCACTTCCTCTTTCCATTCGTTATCCTTGCCGTTACCCT 581

P_minutus_gi|237638806|gb|FJ52 TTCTTTGCCTTCCACTTTCTGTTCCCCTTTGTAGTATTAGCAGCTACTAT 581

GC3_gi|20339610|gb|AF386598.1 TTCTTTGCTTTCCATTTCCTCTTCCCCTTTGTCATTGCCGGCGCTACCCT 581

PF3_gi|164562184|gb|EU348846.1 TTCTTTGCCTTCCACTTCCTATTCCCCTTCGTCATTGCAGGTGCCACCCT 581

CG1_gi|27762429|gb|AY116366.1 TTCTTTGCCTTTCACTTCCTCTTCCCCTTCGTAATTGCAGGTGCAACCCT 581

GA1_gi|25140372|gb|AY116004.1 TTCTTTGCCTTTCACTTCTTATTCCCATTTGTCATTGCTGGTGCCACACT 581

L_cephalus1_gi|12657413|emb|AJ TTCTTCGCATTCCACTTCCTCCTACCGTTTGTCGTCGCAGGCGCAACCAT 581

L_cephalus2_gi|41057843|gb|AY5 TTCTTCGCATTCCACTTCCTCCTGCCATTTGTCGTCGCAGGCGCAACCAT 581

L_cepahlus3_gi|269997201|gb|GU TTTTTCGCATTCCATTTCCTATTACCATTCATCGTCGCCGCCGCAACCCT 581

C_nasus1_gi|18460951|gb|AY0264 TTCTTCGCATTCCACTTCCTACTACCCTTTGTAGTTGCCGGCGCAACCAT 581

L_souffia1_gi|29691926|emb|AJ5 TTCTTCGCATTCCACTTCCTCCTACCATTTGTCATCGCCGGCGCAACCAT 559

R_rutilus1_gi|207113302|gb|FJ0 TTCTTCGCATTCCACTTTCTCTTTCCATTTGTCGTCGCCGGCGCAACCGT 581

B_bjoerkna1_gi|133872335|gb|EF TTCTTCGCATTCCACTTCCTCCTGCCATTCGTTGTAGCCGGCGCAACCCT 581

A_brama2_gi|549466030|gb|KF552 TTCTTCGCATTCCACTTCCTCCTGCCATTCGTTGTAGCCGGCGCAACCCT 581

B_bjoerkna2_gi|2281719|emb|Y10 TTCTTCGCATTCCACTTCCTTCTACCATTCGTTGTCGCCGGCGCAACCCT 581

L_leuciscus1_gi|307090186|gb|H TTCTTCGCATTCCACTTCCTCTTACCATTCGTCGTCGCCGGCGCGACCGT 581

L_leuciscus3_gi|2281727|emb|Y1 TTCTTCGCATTCCACTTCCTCTTACCATTCGTCGTCGCCGGCGCAACCGT 581

A_alburnus1_gi|386367699|gb|JQ TTCTTCGCGTTCCACTTCCTCCTGCCGTTCGTCGTTGCAGGCGCAACCGT 581

A_alburnus2_gi|85679841|gb|DQ3 TTCTTCGCGTTCCACTTTCTCCTGCCGTTCGTCGTTGCAGGCGCAACCGT 566

L_delineatus1_gi|2281725|emb|Y TTCTTCGCATTCCACTTCCTCTTACCTTTCGTTGTTGCGGGCGCGACCGT 581

S_erythrophthalmus1_gi|2140109 TTCTTCGCGTTCCACTTCCTACTGCCGTTCGTCGTCGCAGGCGCAACCAT 581

A_bipunctatus2_gi|2281713|emb| TTCTTCGCGTTCCACTTCCTCCTCCCATTCGTCGTCGCCGGCGCAACCAT 581

A_bipunctatus_gi|5832982|gb|AF TTCTTCGCGTTCCACTTCCTTCTCCCATCTGTCGTCGCCGGCGCAACCAT 581

P_phoxinus1_gi|209968201|gb|EU TTCTTCGCATTTCACTTCCTATTCCCATTTGTTATTGCCGGCGCGACCGT 581

P_phoxinus2_gi|168203857|gb|EU TTCTTCGCATTTCACTTCCTATTTCCCTTTGTTATTGCCGGTGCAACCGT 581

T_tinca1_gi|325169898|gb|HM167 TTCTTCGCATTCCACTTCTTACTGCCATTTATTGTTACCGCCGCCACCCT 581

T_tinca2_gi|444488022|gb|JX974 TTCTTCGCATTCCACTTCTTACTCCCATTCATTGTTGCCGCCGCCACCCT 588

G_gobio1_gi|37904500|gb|AY4265 TTCTTCGCTTTTCACTTTCTCCTACCATTTGTTATTGCCGCCGCAACCGT 581

B_barbus1_gi|462648905|gb|KC46 TTCTTCGCATTTCACTTCCTACTACCATTTATTATTGCTGCCGCAACCAT 581

C_carpio1_gi|68532783|dbj|AB15 TTCTTCGCATTCCACTTCCTACTACCATTTGTTATTGCCGCCGCAACCAT 581

R_amarus1_gi|636792944|dbj|AB3 TTTTTCGCCTTTCACTTCCTTCTTCCATTTGTTATCGTAGCCGCAACCAT 581

B_barbatula2_gi|73671964|gb|DQ TTCTTTGCATTTCACTTTCTCTTCCCATTCGTCATTGCCGGAGCAACCAT 581

C_taenia1_gi|62912083|gb|AY940 TTCTTTGCCTTCCACTTTCTTCTGCCCTTTATTATTGCTGCGGGCACTAT 567

M_fossilis1_gi|117959984|gb|DQ TTCTTTGCTTTCCACTTCCTCTTCCCATTTATCATTGCTGCAGCCACCAT 581

A_anguilla1_gi|159461951|gb|EU TTCTTCGCATTCCACTTCCTATTTCCATTTGTAGTTGCTGGAGCCACAAT 581

T_thymallus_gi|408833805|gb|JX TTCTTTGCCTTCCACTTCTTATTCCCCTTTGTAATTGCAGCTGCCACAGT 581

S_trutta1_gi|1001096|dbj|D5840 TTTTTCGCCTTTCACTTCCTATTCCCATTCGTTATTGCAGCTGCCACAGT 581

E_lucius1_gi|325610655|gb|HM17 TTCTTCGCATTCCACTTCTTATTTCCCTTCATCATTGCAGCAGCCACAAT 581

L_lota1_gi|77386019|gb|DQ17405 TTCTTTGCATTTCATTTTCTATTCCCTTTTATTGTTGCTGCTGTTACAAT 581

L_planeri1_gi|261824331|gb|GQ2 TTTTTTACATTCCATTTTATTTTACCATTTATCCTAGCAGCAATAACTAT 593

NM_gi|671723846|gb|KF549990.1 ACTCCACTTACTATTCCTACACGAAACGGGATCCAATAATCCAGTCGGCC 595

NM_gi|168485493|gb|EU331236.1 ACTCCACTTATTATTCCTACACGAAACGGGATCCAATAATCCAGTCGGCC 631

NF_gi|257220486|gb|GQ444442.1 CCTCCACCTACTATTTCTCCACGAAACGGGCTCCAACAACCCAGTAGGTC 631

NF_gi|671723852|gb|KF549993.1 CCTCCACCTGCTATTTCTCCACGAAACGGGCTCCAACAATCCAGTGGGGC 595

BG_gi|526850801|gb|KF415509.1 AATTCACCTTCTCTTTTTACACGAAACAGGATCTAATAACCCTCTTGGCC 631

BG_gi|188988554|gb|EU444667.1 AATTCACCTTCTCTTTTTACACGAAACAGGATCTAATAACCCTCTTGGCC 631

PK_gi|589911366|gb|KC886260.1 AATTCACCTACTTTTTCTACACGAAACAGGCTCTAATAACCCTATTGGGT 607

PM_gi|526851063|gb|KF415640.1 AGTTCACCTACTCTTTCTACACCAAACAGGATGCAATAACCCACTGGGCC 631

PM_gi|188988552|gb|EU444666.1 AATTCACCTACTTTTTCTACACGAAACAGGGTCCAATAACCCCCTTGGCC 631

G_niger_gi|526850949|gb|KF4155 CCTTCACCTTCTCTTTCTCCACGAGACCGGCTCCAACAACCCCACCGGGC 631

P_minutus_gi|237638806|gb|FJ52 TCTCCACTTGCTATTCCTTCACGAGTCGGGGTCGAACAACCCCACGGGCC 631

GC3_gi|20339610|gb|AF386598.1 TATTCACCTCCTCTTTCTACACGAAACAGGCTCGAACAACCCCTTAGGTT 631

PF3_gi|164562184|gb|EU348846.1 CATCCACCTGCTTTTCCTTCATGAAACAGGCTCAAACAATCCCTTAGGTT 631

CG1_gi|27762429|gb|AY116366.1 CATCCATCTGCTCTTCCTACATGAAACTGGCTCAAACAACCCCCTTGGTT 631

GA1_gi|25140372|gb|AY116004.1 TGTTCACCTTCTTTTCCTTCACCAGACAGGCTCTAATAACCCTCTTGGCC 631

L_cephalus1_gi|12657413|emb|AJ CCTGCACCTGCTGTTTCTACACGAAACAGGATCAAACAACCCAGCCGGAC 631

L_cephalus2_gi|41057843|gb|AY5 TCTACACCTCCTATTTCTACACGAAACGGGATCAAACAACCCAGCCGGAC 631

L_cepahlus3_gi|269997201|gb|GU CCTACACCTACTCTTCCTGCACGAAACAGGATCAAATAACCCAATCGGAC 631

C_nasus1_gi|18460951|gb|AY0264 CCTGCACTTGTTGTTTTTACACGAAACGGGGTCGAACAACCCGGCCGGAC 631

L_souffia1_gi|29691926|emb|AJ5 TCTGCACCTATTATTCTTACACGAAACGGGATCGAACAACCCAGCCGGAC 609

R_rutilus1_gi|207113302|gb|FJ0 TCTACACTTACTGTTTTTACACGAGACGGGATCAAACAACCCGGTCGGAC 631

B_bjoerkna1_gi|133872335|gb|EF CCTACACCTACTATTTCTACACGAAACGGGATCAAATAACCCGACCGGAT 631

A_brama2_gi|549466030|gb|KF552 CCTACACCTACTATTTCTACACGAAACGGGATCAAATAACCCGACCGGAT 631

B_bjoerkna2_gi|2281719|emb|Y10 CTTACACCTACTATTCCTACACGAAACAGGGTCGAACAACCCAGCCGGCT 631

L_leuciscus1_gi|307090186|gb|H CCTACACTTACTATTTCTACACGAAACAGGATCAAATAACCCGGCCGGAC 631

L_leuciscus3_gi|2281727|emb|Y1 CCTACACTTACTATTTCTGCACGAGACAGGATCAAATAACCCGGCCGGAC 631

A_alburnus1_gi|386367699|gb|JQ CCTCCACTTACTATTCCTACACGAGACAGGATCAAACAACCCAGCCGGGT 631

A_alburnus2_gi|85679841|gb|DQ3 CCTCCACTTACTATTCCTACACGAGACAGGATCAAACAACCCAGCCGGGT 616

L_delineatus1_gi|2281725|emb|Y CCTCCACCTTCTATTCCTACACGAGACAGGGTCGAATAACCCGGCCGGAC 631

S_erythrophthalmus1_gi|2140109 CCTACACTTACTGTTTCTACACGAGACAGGATCAAACAACCCAGCCGGAC 631

A_bipunctatus2_gi|2281713|emb| TCTCCACTTGCTATTCCTACACGAGACAGGATCAAACAACCCGGCCGGAT 631

A_bipunctatus_gi|5832982|gb|AF TCTGCACTTGCTATTTCTACACGAGACGGGGTCGAACAATCCGGCCGGAT 631

P_phoxinus1_gi|209968201|gb|EU CCTGCACTTATTGTTTCTACACGAGACAGGATCAAATAACCCCGTTGGAT 631

P_phoxinus2_gi|168203857|gb|EU CCTGCACTTGTTATTTTTACACGAAACAGGCTCGAATAACCCCGCTGGAT 631

T_tinca1_gi|325169898|gb|HM167 CCTACACCTGCTATTTTTACACGAAACAGGAGCAAACAACCCAACAGGAC 631

T_tinca2_gi|444488022|gb|JX974 CCTACACCTGCTATTTTTACACGAAACAGGATCAAACAACCCAACAGGAC 638

G_gobio1_gi|37904500|gb|AY4265 CCTCCACCTACTGTTTTTGCACGAGACAGGATCAAATAACCCAGCCGGAC 631

B_barbus1_gi|462648905|gb|KC46 TCTACATCTCCTATTCCTCCACGAAACAGGGTCAAATAACCCAATTGGAC 631

C_carpio1_gi|68532783|dbj|AB15 CATCCACCTACTGTTCCTCCACGAAACAGGATCAAACAACCCGATCGGAC 631

R_amarus1_gi|636792944|dbj|AB3 CCTCCATCTCCTCTTCCTCCACGAAACAGGGTCAAATAACCCCGCGGGAT 631

B_barbatula2_gi|73671964|gb|DQ CCTCCATATGTTGTTTCTTCATGAAACAGGGTCAAACAACCCGACCGGCC 631

C_taenia1_gi|62912083|gb|AY940 TCTTCACCTACTTTTTCTCCCCGAAACAGGCTCAAACAACCCTATAGGAC 617

M_fossilis1_gi|117959984|gb|DQ CCTTCACCTACTTTTCCTCCATGAAACAGGCTCAAACAATCCCATGGGTA 631

A_anguilla1_gi|159461951|gb|EU ACTTCACCTCCTATTCCTCCATGAAACAGGATCAAACAATCCGGTAGGAT 631

T_thymallus_gi|408833805|gb|JX CCTTCACCTTCTATTCCTTCATGAAACAGGATCCAATAACCCAGCAGGGA 631

S_trutta1_gi|1001096|dbj|D5840 ACTCCACCTTCTATTTTTACATGAAACCGGCTCTAATAACCCAGCAGGTA 631

E_lucius1_gi|325610655|gb|HM17 CCTCCATTTATTATTCCTTCATGAAACTGGCTCAAACAACCCAGTAGGTA 631

L_lota1_gi|77386019|gb|DQ17405 ACTACACCTACTATTTCTTCACGAAACAGGCTCAAATAACCCAACAGGAA 631

L_planeri1_gi|261824331|gb|GQ2 AATCCATATTATATTCCTCCATCAAACAGGATCTAGTAACCCCCTAGGAA 643

NM_gi|671723846|gb|KF549990.1 TTAATTCAGATGCCGACAAAATTCCTTTTCACCCTTACTTTACCTATAAA 645

NM_gi|168485493|gb|EU331236.1 TTAATTCAGATGCCGACAAAATTCCTTTTCACCCTTACTTTACCTATAAA 681

NF_gi|257220486|gb|GQ444442.1 TTAACTCAGATGCCGACAAAATTCCTTTTCACCCCTACTTTACCTATAAA 681

NF_gi|671723852|gb|KF549993.1 TTAACTCAGACGCCGACAAAATTCCTTTCCACCCCTACTTTACCTATAAA 645

BG_gi|526850801|gb|KF415509.1 TTAACTCAAACGCCGACAAAATCCCCTTTCACCCCTATTTTATCTATAAA 681

BG_gi|188988554|gb|EU444667.1 TTAACTCAAACGCCGACAAAATCCCCTTTCACCCCTATTTTATCTATAAA 681

PK_gi|589911366|gb|KC886260.1 TGAACTCAGACGCCGACAAAATCCCCTTCCACCCCTACTTTATTTATAAA 657

PM_gi|526851063|gb|KF415640.1 TTAACTCTAATGCAGATAAAATCCCATTCCACCCCTACTTCACTTACAAA 681

PM_gi|188988552|gb|EU444666.1 TTAACTCAAACGCAGACAAAATCCCATTTCACCCCTACTTCATCTATAAA 681

G_niger_gi|526850949|gb|KF4155 TTAATTCTGACGCGGACAAGGTTCCCTTTCACCCTTACTTCTCCTACAAG 681

P_minutus_gi|237638806|gb|FJ52 TGAACTCCGACGCCGATAAGGTTCCCTTCCACCCATACTTCTCCTACAAG 681

GC3_gi|20339610|gb|AF386598.1 TAAACTCTGACGCCGACAAAGTCTCTTTCCACCCCTATTTCTCATATAAA 681

PF3_gi|164562184|gb|EU348846.1 TAAACTCTGACGCAGATAAAGTTTCCTTTCATCCTTATTTTTCTTACAAA 681

CG1_gi|27762429|gb|AY116366.1 TAAACTCAGATGCAGACAAAATCTCTTTCCACCCTTACTTTTCTTACAAA 681

GA1_gi|25140372|gb|AY116004.1 TGAACTCAGACGCTGACAAAATCTCCTTTCACCCCTACTTTTCTTATAAA 681

L_cephalus1_gi|12657413|emb|AJ TAAACTCCGACGCCGACAAAATTTCTTTCCACCCATACTTCTCATATAAA 681

L_cephalus2_gi|41057843|gb|AY5 TAAATTCCGACGCCGACAAAATTTCTTTCCACCCGTACTTCTCATATAAA 681

L_cepahlus3_gi|269997201|gb|GU TAAACTCCGACGCAGATAAAATTTCCTTCCACCCATACTTCTCATATAAA 681

C_nasus1_gi|18460951|gb|AY0264 TAAATTCAGACGCCGACAAAATTTCTTTCCACCCGTACTTCTCATATAAA 681

L_souffia1_gi|29691926|emb|AJ5 TAAATTCCGACGCGGACAAAATTTCTTTCCACCCATACTTCTCATATAAG 659

R_rutilus1_gi|207113302|gb|FJ0 TAAACTCCGACGCAGATAAAATTTCTTTCCACCCGTATTTTTCATACAAA 681

B_bjoerkna1_gi|133872335|gb|EF TAAACTCCGACGCAGATAAAATTTCTTTCCACCCATACTTCTCATATAAA 681

A_brama2_gi|549466030|gb|KF552 TAAACTCCGACGCAGATAAAATTTCTTTCCACCCATACTTCTCATATAAA 681

B_bjoerkna2_gi|2281719|emb|Y10 TAAACTCCGACGCAGATAAAATTTCCTTCCACCCGTACTTCTCATATAAA 681

L_leuciscus1_gi|307090186|gb|H TAAATTCTGACGCGGACAAAATTTCCTTCCACCCTTACTTCTCATACAAA 681

L_leuciscus3_gi|2281727|emb|Y1 TAAATTCTGACGCGGACAAAATTTCCTTCCACCCATACTTCTCATATAAA 681

A_alburnus1_gi|386367699|gb|JQ TAAACTCTGACGCGGATAAAATTTCTTTCCACCCATACTTCTCCTACAAA 681

A_alburnus2_gi|85679841|gb|DQ3 TAAACTCTGACGCGGACAAAATTTCTTTCCACCCATACTTCTCCTACAAA 666

L_delineatus1_gi|2281725|emb|Y TAAATTCTGACGCGGACAAAATTTCTTTCCACCCCTACTTCTCTTACAAA 681

S_erythrophthalmus1_gi|2140109 TAAATTCCGACGCGGACAAAATTTCCTTCCACCCGTACTTCTCATACAAG 681

A_bipunctatus2_gi|2281713|emb| TAAATTCCGACGCAGACAAAATTTCTTTCCACCCATACTTCTCATATAAA 681

A_bipunctatus_gi|5832982|gb|AF TAAACTCCGATGCAGACAAGATTTCTTTCCACCCATACTTCTCATATAAA 681

P_phoxinus1_gi|209968201|gb|EU TAAACTCAGATGCAGATAAAATTTCTTTTCACCCCTACTTTTCTTATAAA 681

P_phoxinus2_gi|168203857|gb|EU TAAACTCGGATGCGGATAAAATTTCTTTTCACCCATACTTTTCTTATAAA 681

T_tinca1_gi|325169898|gb|HM167 TAAACTCCGACGCAGACAAAATCTCCTTCCACCCCTACTTTTCATATAAA 681

T_tinca2_gi|444488022|gb|JX974 TAAACTCCGACGCAGACAAAATCTCCTTCCACCCCTACTTTTCATATAAA 688

G_gobio1_gi|37904500|gb|AY4265 TAAACTCTGACGCAGACAAAATCTCATTCCACCCATACTTCTCTTACAAG 681

B_barbus1_gi|462648905|gb|KC46 TAAACTCAGACGCAGACAAAATCTCCTTCCACCCATACTTTACCTACAAA 681

C_carpio1_gi|68532783|dbj|AB15 TAAACTCAGACGCAGACAAAGTCTCTTTCCACCCGTACTTCTCATACAAA 681

R_amarus1_gi|636792944|dbj|AB3 TAAACTCCGACGCAGATAAAATTTCTTTCCACCCCTACTTCTCCTACAAA 681

B_barbatula2_gi|73671964|gb|DQ TAAACTCAGACGCAGACAAAATCACCTTCCACCCCTATTTCTCCTATAAA 681

C_taenia1_gi|62912083|gb|AY940 TAAATTCAGACGCAGATAAAGTATCATTTCACCCATATTTCTCATATAAA 667

M_fossilis1_gi|117959984|gb|DQ TTAACTCAGACGCAGATAAAGTCTCATTTCACCCCTACTTTTCATATAAA 681

A_anguilla1_gi|159461951|gb|EU TAAACTCCGACGCAGACAAAATCCCATTCCACCCATACTTCTCCTACAAA 681

T_thymallus_gi|408833805|gb|JX TTAACTCCGATGCCGATAAAATCTCGTTTCACCCTTACTTCTCATACAAA 681

S_trutta1_gi|1001096|dbj|D5840 TCAACTCCGATGCCGATAAAATCTCATTCCACCCATACTTCTCATACAAA 681

E_lucius1_gi|325610655|gb|HM17 TTAACTCTGATGCAGATAAAATCCCCTTTCATCCCTACTTCTCCTACAAA 681

L_lota1_gi|77386019|gb|DQ17405 TTAACTCAAATGCAGACAAAATCCCCTTCCACCCCTACTTTACTTATAAA 681

L_planeri1_gi|261824331|gb|GQ2 TTAACTCTAATTTGGATAAAATTCAATTTCACCCATACTTCTCTTTCAAA 693

NM_gi|671723846|gb|KF549990.1 GACCTACTAGGGTTTGCCATCATAATATTTGCTTTAACCTCTCTCGCCCT 695

NM_gi|168485493|gb|EU331236.1 GACCTACTAGGGTTTGCCATCATAATATTTGCTTTAACCTCTCTCGCCCT 731

NF_gi|257220486|gb|GQ444442.1 GATTTGTTAGGGTTTGCCATCATATTACTAGCCCTAACCTCTCTTGCCCT 731

NF_gi|671723852|gb|KF549993.1 GATTTATTAGGATTTGCCATCATATTACTTGCTCTTGCCTCTCTTGCCCT 695

BG_gi|526850801|gb|KF415509.1 GACCTGCTAGGTTTTGTTATTATATTATTTGCCCTAACCTCCCTAGCACT 731

BG_gi|188988554|gb|EU444667.1 GACCTGCTAGGTTTTGTTATTATATTATTTGCCCTAACCTCCCTAGCACT 731

PK_gi|589911366|gb|KC886260.1 GACCTGCTAGGTTTTGCTATTATATTACTTGCCCTAACCTCCTTAGCATT 707

PM_gi|526851063|gb|KF415640.1 GACTTACTAGGGTTTGCCCTCATACTAATTTCCCTAACCTGTCTGGCACT 731

PM_gi|188988552|gb|EU444666.1 GACTTATTAGGATTTGCCATCATATTAATTTCCCTAACCTGCTTAGCACT 731

G_niger_gi|526850949|gb|KF4155 GACCTCCTTGGATTCGCCATCATACTCTTCGCCCTCACCTCCTTGGCTCT 731

P_minutus_gi|237638806|gb|FJ52 GATCTTATCGGTTTCGCCGTAATGCTAGTTGCGCTCACCTCACTCGCCCT 731

GC3_gi|20339610|gb|AF386598.1 GACCTCTTAGGCTTTGCAGTCCTCCTTATTACCTTAACATCTCTAGCCCT 731

PF3_gi|164562184|gb|EU348846.1 GACCTCTTAGGATTTGCAGTACTACTAATCGCTCTAACAGCTCTAGCTCT 731

CG1_gi|27762429|gb|AY116366.1 GACCTGTTAGGCTTTGCAGCACTCTTAATTGCCCTCACAGCCCTAGCACT 731

GA1_gi|25140372|gb|AY116004.1 GACCTCCTTGGCTTCGCAGCCCTACTAATTGCCCTTACATCCCTAGCACT 731

L_cephalus1_gi|12657413|emb|AJ GACCTTCTTGGCTTTGTTATCATATTACTAGCCCTTACCTCTCTAGCATT 731

L_cephalus2_gi|41057843|gb|AY5 GACCTTCTTGGCTTTGTTATTATACTACTAGCCCTTACCTCTCTAGCACT 731

L_cepahlus3_gi|269997201|gb|GU GACCTTCTTGGCTTTGTTATTATACTACTAGCCCTTACCTCCCTAGCACT 731

C_nasus1_gi|18460951|gb|AY0264 GATCTCCTTGGTTTTGTGGCAATGTTACTAGCCCTTACCTCTCTGACATT 731

L_souffia1_gi|29691926|emb|AJ5 GACCTTCTTGGCTTTGTGGCAATGTTACTAGCCCTCACCTCTCTAACCTT 709

R_rutilus1_gi|207113302|gb|FJ0 GACCTCCTTGGCTTTGTAATTATGTTACTAGCTCTCACCTCCCTAACATT 731

B_bjoerkna1_gi|133872335|gb|EF GACCTTCTTGGCTTTGTAATCATGTTACTAGCCCTCACTTCGCTAGCATT 731

A_brama2_gi|549466030|gb|KF552 GACCTTCTTGGCTTTGTAATCATGTTACTAGCCCTCACTTCGCTAGCATT 731

B_bjoerkna2_gi|2281719|emb|Y10 GACCTTCTTGGCTTTGTAATCATATTACTGGCCCTTACTTCCCTGGCATT 731

L_leuciscus1_gi|307090186|gb|H GACCTTCTTGGCTTTGTGATCATACTGCTAGCCCTCACCTCTCTGACACT 731

L_leuciscus3_gi|2281727|emb|Y1 GACCTTCTTGGCTTGGTGATCATACTGCTAGCCCTCACCTCGCTAACGCT 731

A_alburnus1_gi|386367699|gb|JQ GACCTTCTCGGCTTCGTAATCATGCTGCTCGCCCTCACCTCGCTGGCGCT 731

A_alburnus2_gi|85679841|gb|DQ3 GACCTTCTCGGCTTCGTAATCATGCTGCTGGCCCTCACCTCGCTGGCGCT 716

L_delineatus1_gi|2281725|emb|Y GACCTTCTCGGCTTCGTAATTATATTGCTAGCCCTCACCTCGCTGGCGCT 731

S_erythrophthalmus1_gi|2140109 GACCTTCTTGGCTTCGTAATTATGCTGCTTGCCCTTACAGCTTTGGCACT 731

A_bipunctatus2_gi|2281713|emb| GACCTTCTTGGCTTTGTAATCATGTTACTAGCCCTCACCTCCCTAACGCT 731

A_bipunctatus_gi|5832982|gb|AF GATCTCCTTGGCTTTGTAATCATGTTACTAGCCCTCACCTCCCTGACCCT 731

P_phoxinus1_gi|209968201|gb|EU GACCTTCTAGGGTTTGCAGTGATACTACTAGCTCTTACATCACTAACCCT 731

P_phoxinus2_gi|168203857|gb|EU GACCTTCTAGGCTTTGTAGTAATACTACTAGCTCTTACATCACTAACCCT 731

T_tinca1_gi|325169898|gb|HM167 GACCTTCTAGGGTTCGTAATTATATTATTAGCCCTCACATCACTAGCACT 731

T_tinca2_gi|444488022|gb|JX974 GACCTTCTAGGGTTCGTAATTATATTATTAGCCCTCACATCACTAGCACT 738

G_gobio1_gi|37904500|gb|AY4265 GACCTTCTTGGTTTTGTCCTAATACTCCTAGCTCTTACATCATTAGCACT 731

B_barbus1_gi|462648905|gb|KC46 GACCTACTCGGGTTCGTAATTATACTACTAGCCCTTACACTACTAGCATT 731

C_carpio1_gi|68532783|dbj|AB15 GACCTCCTTGGGTTCGTAATTATACTCCTAGCTCTTACACTACTAGCACT 731

R_amarus1_gi|636792944|dbj|AB3 GATCTGCTAGGATTCGTACTTATACTAATAACCTTAACAGCCTTAGCATT 731

B_barbatula2_gi|73671964|gb|DQ GACCTACTTGGCTTCGTAGTTATGCTACTAGCCCTTACATCCTTAGTATT 731

C_taenia1_gi|62912083|gb|AY940 GACTTATTAGGCTTTGCAGTAGTCCTTCTGGCATTGACCTCACTTTCACT 717

M_fossilis1_gi|117959984|gb|DQ GATTTGCTAGGATTTGCAGTTGTTCTACTTGCCCTTACATCTCTATCACT 731

A_anguilla1_gi|159461951|gb|EU GATCTACTGGGGTTCATTATCATGCTCACCGCCCTAACAATACTTGCCCT 731

T_thymallus_gi|408833805|gb|JX GACCTCCTTGGATTTGTAGCCATGCTTCTAGGTTTAACATCCCTAGCCCT 731

S_trutta1_gi|1001096|dbj|D5840 GACCTCCTTGGATTCGTAGCTATACTACTTGGCCTAACATCATTAGCTCT 731

E_lucius1_gi|325610655|gb|HM17 GACCTCTTAGGTTTTGTATTTATGTTATTTGGCTTAGCCTCCCTAGCCCT 731

L_lota1_gi|77386019|gb|DQ17405 GACCTTCTAGGTTTTGCCGTAATACTCCTTGGCCTTACTGCCCTTGCCCT 731

L_planeri1_gi|261824331|gb|GQ2 GACATTTTTGGCTTTGTTATTTTACTTGGGGTTCTTTTTATAATTTCTCT 743

NM_gi|671723846|gb|KF549990.1 TTTTTCCCCAAACTTCCTCGGAGACCCAGACAACTTCATCCCAGCAAACC 745

NM_gi|168485493|gb|EU331236.1 TTTTTCCCCAAACTTTCTCGGAGACCCAGACAACTTCATCCCAGCAAACC 781

NF_gi|257220486|gb|GQ444442.1 CTTTACCCCAAATTACCTAGGAGATCCCGACAACTTCATCCCAGCAAACC 781

NF_gi|671723852|gb|KF549993.1 CTTTACCCCAAACTACCTAGGAGACCCGGACAATTTCATCCCAGCAAATC 745

BG_gi|526850801|gb|KF415509.1 ATTTTCCCCTAATTATCTCGGAGACCCCGACAACTTTATTCCAGCAAATC 781

BG_gi|188988554|gb|EU444667.1 ATTTTCCCCTAATTATCTCGGAGACCCCGACAACTTTATTCCAGCAAATC 781

PK_gi|589911366|gb|KC886260.1 ATTTATCCCCAACTACCTTGGAGACCCCGACAACTTTATCCCAGCAAACC 757

PM_gi|526851063|gb|KF415640.1 ATTTTTACCTAACTATCTAGGAGACCCTGACAACTTCATCCCCGCAAACC 781

PM_gi|188988552|gb|EU444666.1 GTTTTTACCTAACTACCTTGGGGACCCTGACAACTTCATTCCCGCAAATC 781

G_niger_gi|526850949|gb|KF4155 TTTTACCCCCAACTACCTAGGGGATCCGGACAACTTCATTCCTGCCAACC 781

P_minutus_gi|237638806|gb|FJ52 GTTCTCCCCCAACTACCTTGGCGACCCAGACAACTTCACCCCCGCCAATC 781

GC3_gi|20339610|gb|AF386598.1 TTTCTCCCCTAACCTCTTAGGAGACCCAGATAATTTTACCCCTGCAAACC 781

PF3_gi|164562184|gb|EU348846.1 CTTCTCCCCTAATTTATTAGGGGATCCTGACAACTTTACCCCAGCCAACC 781

CG1_gi|27762429|gb|AY116366.1 CTTCTCCCCTAACCTCTTAGGAGACCCAGATAACTTTACTCCTGCTAACC 781

GA1_gi|25140372|gb|AY116004.1 ATTTGCTTCTAACCTGCTTGGAGATCCCGATAACTTCACCCCTGCAAACC 781

L_cephalus1_gi|12657413|emb|AJ ATTTTCTCCCAACCTACTGGGTGACCCAGAAAACTTTACCCCAGCAAACC 781

L_cephalus2_gi|41057843|gb|AY5 ATTTTCTCCTAACCTACTAGGTGACCCAGAAAACTTTACCCCAGCAAACC 781

L_cepahlus3_gi|269997201|gb|GU ATTTTCTCCTAACCTACTAGGTGACCCAGAAAACTTTACCCCAGCAAACC 781

C_nasus1_gi|18460951|gb|AY0264 ATTTTCCCCTAACCTATTAGGTGACCCGGAAAACTTTACCCCAGCAAACC 781

L_souffia1_gi|29691926|emb|AJ5 ATTCTCCCCTAACCTCTTAGGTGACCCGGAGAACTTCACCCCAGCAAACC 759

R_rutilus1_gi|207113302|gb|FJ0 ATTTTCTCCTAACCTATTAGGTGACCCAGAAAACTTTACGCCAGCAAACC 781

B_bjoerkna1_gi|133872335|gb|EF ATTTTCACCTAATCTACTAGGTGACCCAGAAAATTTTACCCCAGCAAACC 781

A_brama2_gi|549466030|gb|KF552 ATTTTCACCTAATCTACTAGGTGACCCAGAAAATTTTACCCCAGCAAACC 781

B_bjoerkna2_gi|2281719|emb|Y10 ATTTTCCCCCAACCTACTAGGTGACCCAGAAAATTTTACCCCAGCAAACC 781

L_leuciscus1_gi|307090186|gb|H ATTTTCCCCTAATCTTCTAGGTGACCCAGAGAATTTTACCCCAGCAAACC 781

L_leuciscus3_gi|2281727|emb|Y1 ATTTTCCCCTAATCTTCTAGGTGACCCAGAGAATTTTACCCCAGCAAACC 781

A_alburnus1_gi|386367699|gb|JQ ATTTTCCCCCAACCTCCTAGGTGATCCAGAGAACTTTACCCCAGCAAACC 781

A_alburnus2_gi|85679841|gb|DQ3 ATTTTCCCCTAACCTCCTAGGTGATCCGGAGAACTTTACCCCAGCAAACC 766

L_delineatus1_gi|2281725|emb|Y GTTCTCGCCCAACCTTTTAGGTGATCCAGAAAACTTTACCCCAGCAAACC 781

S_erythrophthalmus1_gi|2140109 ATTTTCCCCAAACCTTTTAGGGGACCCTGAGAATTTTACTCCAGCAAACC 781

A_bipunctatus2_gi|2281713|emb| ATTTTCTCCCAACCTACTAGGTGACCCAGAAAATTTTACCCCTGCGAACC 781

A_bipunctatus_gi|5832982|gb|AF ATTTTCTCCGAACCTATTAGGTGACCCAGAAAATTTTACCCCTGCGAACC 781

P_phoxinus1_gi|209968201|gb|EU ATTTTCACCAAGTCTACTAGGTGACCCAGAAAATTTTACTCCTGCTAACC 781

P_phoxinus2_gi|168203857|gb|EU GTTTTCACCGAGCCTGTTGGGTGACCCAGAAAATTTTACCCCTGCGAACC 781

T_tinca1_gi|325169898|gb|HM167 ATTCTCTCCAAACTTATTAGGAGACCCAGAAAATTTTACCCCAGCAAACC 781

T_tinca2_gi|444488022|gb|JX974 ATTCTCTCCAAACTTATTAGGAGACCCAGAAAATTTTACCCCAGCAAACC 788

G_gobio1_gi|37904500|gb|AY4265 GTTCTCCCCTAACTTGCTTGGGGACCCGGACAACTTCACCCCCGCAAACC 781

B_barbus1_gi|462648905|gb|KC46 ATTTTCCCCTAACCTACTAGGAGACCCGGAAAACTTTACCCCCGCCAACC 781

C_carpio1_gi|68532783|dbj|AB15 ATTCTCCCCTAACTTACTAGGAGACCCAGAAAACTTCACCCCCGCAAACC 781

R_amarus1_gi|636792944|dbj|AB3 ATTTTCACCTAACCTACTAGGTGACCCAGAAAATTTTACTCCTGCCAACC 781

B_barbatula2_gi|73671964|gb|DQ ATTTACCCCCGGCCTTTTAGGAGACCCAGAGAACTTCACACCTGCAAACC 781

C_taenia1_gi|62912083|gb|AY940 ATTTTCCCCTAATCTTCTTGGAGACCCCGATAACTTCACCCCCGCAAATC 767

M_fossilis1_gi|117959984|gb|DQ GTTCTCCCCAAACCTCCTAGGCGACCCGGACAACTTTACCCCCGCCAACC 781

A_anguilla1_gi|159461951|gb|EU ATTCTACCCGAACCTGCTTGGAGACCCAGACAACTTCACCCCGGCAAATC 781

T_thymallus_gi|408833805|gb|JX ATTTGCACCTAATCTTCTAGGAGACCCGGACAATTTTACACCAGCCAACC 781

S_trutta1_gi|1001096|dbj|D5840 GTTCGCACCCAACCTCCTCGGAGACCCGGACAATTTTACGCCTGCCAACC 781

E_lucius1_gi|325610655|gb|HM17 CTTCTCACCCAACCTTCTGGGAGACCCAGACAACTTCATTCCTGCCAACC 781

L_lota1_gi|77386019|gb|DQ17405 CTTCTCCCCCAACCTGCTTGGAGACCCAGACAATTTTACACCCGCTAACC 781

L_planeri1_gi|261824331|gb|GQ2 TTTAGCCCCAAATGCACTAGGTGAGCCAGACAATTTTATTTATGCCAACC 793

NM_gi|671723846|gb|KF549990.1 CCCTCGTCACTCCCCCCCACATCAAACCTGAATGGTACTTCTTATTTGCT 795

NM_gi|168485493|gb|EU331236.1 CCCTCGTCACTCCCCCCCACATCAAACCTGAATGATACTTCTTATTTGCT 831

NF_gi|257220486|gb|GQ444442.1 CCCTCGTTACTCCGCCCCACATTAAACCTGAGTGATACTTCTTATTTGCT 831

NF_gi|671723852|gb|KF549993.1 CCCTTGTCACCCCTCCCCACATTAAGCCTGAGTGGTACTTCTTATTTGCT 795

BG_gi|526850801|gb|KF415509.1 CCCTAGTTACCCCGCCCCACATCAAGCCTGAATGATACTTCCTATTTGCT 831

BG_gi|188988554|gb|EU444667.1 CCCTAGTTACCCCGCCCCACATCAAGCCTGAATGATACTTCCTATTTGCT 831

PK_gi|589911366|gb|KC886260.1 CACTAGTTACCCCACCCCACATTAAGCCTGAGTGGTACTTCCTGTTCGCC 807

PM_gi|526851063|gb|KF415640.1 CCTTAATAACTCCCCCCCATATCAAACCCGAGTGGTACTTCTTATTTGCC 831

PM_gi|188988552|gb|EU444666.1 CCTTAATAACCCCACCTCATATTAAGCCCGAATGATATTTCTTATTTGCC 831

G_niger_gi|526850949|gb|KF4155 CCCTTGTTACCCCGCCTCACATTAAACCAGAGTGATACTTTCTTTTTGCC 831

P_minutus_gi|237638806|gb|FJ52 CTCTGGTTACCCCACCCCACATCAAGCCTGAGTGGTACTTCTTGTTTGCC 831

GC3_gi|20339610|gb|AF386598.1 CCCTAGTTACACCACCTCACATCAAACCTGAGTGATACTTCCTATTTGCA 831

PF3_gi|164562184|gb|EU348846.1 CCTTAGTTACCCCACCACACATCAAGCCTGAATGATACTTCTTGTTTGCC 831

CG1_gi|27762429|gb|AY116366.1 CCCTGGTAACCCCTCCCCACATCAAGCCTGAATGATACTTCTTGTTTGCC 831

GA1_gi|25140372|gb|AY116004.1 CATTAGTCACTCCACCTCACATCAAGCCTGAATGGTACTTCTTGTTTGCC 831

L_cephalus1_gi|12657413|emb|AJ CACTAGTGACGCCCCCACATATTCAGCCAGAATGGTACTTCTTATTTGCC 831

L_cephalus2_gi|41057843|gb|AY5 CACTAGTAACACCCCCACATATTCAGCCAGAGTGATACTTCTTATTTGCC 831

L_cepahlus3_gi|269997201|gb|GU CACTAGTAACACCCCCACATATTCAGCCAGAGTGATACTTCTTATTTGCC 831

C_nasus1_gi|18460951|gb|AY0264 CGCTCGTGACACCACCGCATATCCAGCCAGAATGATACTTCTTATTTGCC 831

L_souffia1_gi|29691926|emb|AJ5 CACTCGTAACGCCTCCACATATCCAGCCAGAGTGGTACTTCTTGTTTGCC 809

R_rutilus1_gi|207113302|gb|FJ0 CACTCGTGACACCCCCACATATTCAGCCAGAATGATACTTCTTATTTGCC 831

B_bjoerkna1_gi|133872335|gb|EF CACTCGTGACACCCCCGCATATTCAGCCAGAATGATACTTCTTATTTGCC 831

A_brama2_gi|549466030|gb|KF552 CACTCGTGACGCCCCCGCATATTCAGCCAGAATGATACT----------- 820

B_bjoerkna2_gi|2281719|emb|Y10 CACTCGTGACACCCCCACATATTCAGCCAGAGTGATATTTCCTATTTGCC 831

L_leuciscus1_gi|307090186|gb|H CACTCGTGACTCCCCCACATATTCAGCCAGAATGATACTTCCTGTTTGCC 831

L_leuciscus3_gi|2281727|emb|Y1 CACTCGTGACTCCCCCACATATTCAGCCAGAGTGATACTTCCTATTTGCC 831

A_alburnus1_gi|386367699|gb|JQ CACTTGTGACACCCCCACATATCCAACCAGAGTGATACTTCTTGTTTGCA 831

A_alburnus2_gi|85679841|gb|DQ3 CACTTGTGACACCCCCACATATTCAACCAGAGTGATACTTCTTGTTTGCA 816

L_delineatus1_gi|2281725|emb|Y CTCTCGTGACACCCCCACATATCCAGCCAGAATGATACTTCTTATTTGCG 831

S_erythrophthalmus1_gi|2140109 CACTCGTGACACCCCCACACATCCAGCCAGAGTGATATTTCTTATTTGCC 831

A_bipunctatus2_gi|2281713|emb| CCCTTGTGACACCCCCACACATCCAACCTGAGTGATACTTCCTGTTTGCC 831

A_bipunctatus_gi|5832982|gb|AF CACTTGTAACGCCCCCACACATCCAACCAGAGTGATACTTCTTGTTTGCC 831

P_phoxinus1_gi|209968201|gb|EU CCCTCGTTACCCCGCCTCATATTCAACCAGAATGGTATTTCCTGTTTGCC 831

P_phoxinus2_gi|168203857|gb|EU CCCTCGTTACCCCGCCTCATATTCAGCCAGAGTGATATTTCCTGTTTGCC 831

T_tinca1_gi|325169898|gb|HM167 CCTTAGTCACACCTCCACACATTCAGCCAGAATGATATTTCTTATTTGCC 831

T_tinca2_gi|444488022|gb|JX974 CCTTAGTCACACCTCCACACATTCAGCCAGAATGATATTTCTTATTTGCC 838

G_gobio1_gi|37904500|gb|AY4265 CGATAGTTACCCCTCCCCACATTAAACCCGAGTGGTATTTCCTATTTGCT 831

B_barbus1_gi|462648905|gb|KC46 CCCTAGTCACCCCTCCACATATCAAACCAGAATGATACTTCCTATTTGCC 831

C_carpio1_gi|68532783|dbj|AB15 CTCTAGTCACACCACCCCACATCAAACCAGAATGATACTTCCTATTTGCC 831

R_amarus1_gi|636792944|dbj|AB3 CACTCGTTACACCACCACACATCCAGCCCGAATGATATTTTCTATTTGCC 831

B_barbatula2_gi|73671964|gb|DQ CCCTAGTCACCCCACCACACATTCAACCTGAATGATATTTCCTTTTCGCC 831

C_taenia1_gi|62912083|gb|AY940 CCCTAGTGACACCTCCCCATATTAAACCAGAATGATATTTTCTATTTGCA 817

M_fossilis1_gi|117959984|gb|DQ CTTTAGTAACACCCCCTCATATTAAACCAGAATGATACTTTTTATTTGCC 831

A_anguilla1_gi|159461951|gb|EU CAATAGTTACTCCGCCACACATTAAGCCAGAGTGGTATTTTCTATTTGCC 831

T_thymallus_gi|408833805|gb|JX CACTGGTCACCCCTCCCCACATCAAGCCTGAGTGATACTTCTTGTTTGCC 831

S_trutta1_gi|1001096|dbj|D5840 CCCTAGTCACCCCACCTCATATCAAGCCCGAATGATACTTCCTATTCGCC 831

E_lucius1_gi|325610655|gb|HM17 CCTTGGTTACTCCACCCCATATTAAACCAGAGTGATATTTTTTATTCGCC 831

L_lota1_gi|77386019|gb|DQ17405 CGATTGTTACCCCTCCACATGTCAAGCCTGAGTGGTATTTCCTATTTGCC 831

L_planeri1_gi|261824331|gb|GQ2 CTCTTAGTACCCCACCTCACATTAAGCCAGAATGATACTTCCTGTTCGCC 843

NM_gi|671723846|gb|KF549990.1 TACGCCATCCTCCGCTCTATCCCTAACAAACTTGGGGGAGTCCTAGCACT 845

NM_gi|168485493|gb|EU331236.1 TACGCCATCCTCCGCTCTATCCCTAACAAACTTGGAGGAGTCCTAGCACT 881

NF_gi|257220486|gb|GQ444442.1 TACGCCATCCTACGCTCCATCCCCAATAAACTTGGAGGAGTCCTAGCATT 881

NF_gi|671723852|gb|KF549993.1 TACGCCATTCTCCGCTCCATCCCCAATAAACTTGGGGGAGTACTAGCATT 845

BG_gi|526850801|gb|KF415509.1 TACGCCATTCTACGCTCCATCCCTGATAAATTGGGAGGAGTAATAGCACT 881

BG_gi|188988554|gb|EU444667.1 TACGCCATTCTACGCTCCATCCCTGATAAATTGGGAGGAGTAATAGCACT 881

PK_gi|589911366|gb|KC886260.1 TATGCCATTTTACGCTCCATCCCTGACAAGTTAGGGGGAGTCATAGCACT 857

PM_gi|526851063|gb|KF415640.1 TACGCCATCTTACGCTCCATTCCCAACAAACTTGGGGGAGTTATAGCACT 881

PM_gi|188988552|gb|EU444666.1 TACGCCATCTTGCGCTCTATTCCTAATAAACTTGGGGGAGTAATAGCACT 881

G_niger_gi|526850949|gb|KF4155 TACGCAATCTTGCGCTCCATTCCTAACAAACTTGGAGGAGTCCTTGCCCT 881

P_minutus_gi|237638806|gb|FJ52 TACGCCATCCTGCGATCTATCCCTAACAAGCTTGGAGGAGTCCTCGCCCT 881

GC3_gi|20339610|gb|AF386598.1 TACGCAATCCTTCGCTCCATCCCCAACAAGCTAGGAGGAGTTTTAGCCCT 881

PF3_gi|164562184|gb|EU348846.1 TACGCCATCTTACGCTCGATTCCAAACAAACTAGGAGGGGTGTTAGCCTT 881

CG1_gi|27762429|gb|AY116366.1 TACGCCATCCTTCGCTCTATCCCCAACAAGCTTGGAGGCGTCCTAGCCCT 881

GA1_gi|25140372|gb|AY116004.1 TACGCCATTCTTCGATCTATCCCAAATAAACTTGGAGGCGTCCTTGCACT 881

L_cephalus1_gi|12657413|emb|AJ TACGCCATTCTCCGGTCTATCCCAAACAAACTAGGAGGGGTCCTGGCGCT 881

L_cephalus2_gi|41057843|gb|AY5 TACGCCATTCTCCGATCTATCCCAAACAAACTAGGAGGGGTACTTGCACT 881

L_cepahlus3_gi|269997201|gb|GU TACGCCATTCTCCGATCTATCCCAAACAAACTAGGAGGGGTACTTGCACT 881

C_nasus1_gi|18460951|gb|AY0264 TACGCCATCCTTCGGTCTATCCCAAATAAACTAGGAGGGGTTCTTGCACT 881

L_souffia1_gi|29691926|emb|AJ5 TACGCCATCCTCCGATCTATCCCAAATAAGCTAGGAGGGGTTCTTGCACT 859

R_rutilus1_gi|207113302|gb|FJ0 TACGCCATCCTCCGATCCATCCCGAACAAGCTAGGAGGGGTCCTCGCACT 881

B_bjoerkna1_gi|133872335|gb|EF TACGCTATTCTCCGATCCATTCCAAATAAATTAGGAGGGGTTCTTGCATT 881

A_brama2_gi|549466030|gb|KF552 --------------------------------------------------

B_bjoerkna2_gi|2281719|emb|Y10 TACGCCATTCTCCGATCCATTCCAAATAAACTAGGAGGGGTCCTTGCATT 881

L_leuciscus1_gi|307090186|gb|H TACGCCATTCTCCGGTCCATCCCAAACAAACTAGGAGGGGTTCTTGCACT 881

L_leuciscus3_gi|2281727|emb|Y1 TACGCCATTCTCCGATCTATCCCAAACAAACTAGGAGGGGTTCTTGCACT 881

A_alburnus1_gi|386367699|gb|JQ TACGCCATCCTCCGGTCTATTCCTAATAAACTAGGCGGGGTTCTTGCACT 881

A_alburnus2_gi|85679841|gb|DQ3 TACGCCATCCTCCGGTCTATTCCTAATAAACTAGGCGGGGTTCTTGCACT 866

L_delineatus1_gi|2281725|emb|Y TATGCCATCCTCCGGTCTATTCCTAATAAACTAGGCGGGGTTCTTGCTCT 881

S_erythrophthalmus1_gi|2140109 TACGCCATCCTCCGATCCATCCCAAATAAACTAGGAGGGGTTCTTGCACT 881

A_bipunctatus2_gi|2281713|emb| TATGCCATCCTACGATCTATCCCAAATAAACTTGGAGGGGTCCTTGCGCT 881

A_bipunctatus_gi|5832982|gb|AF TATGCTATCCTGCGATCTATTCCAAATAAACTTGGAGGGGTCCTTGCGCT 881

P_phoxinus1_gi|209968201|gb|EU TACGCTATCTTACGATCTATCCCTAACAAGTTGGGGGGTGTCCTTGCACT 881

P_phoxinus2_gi|168203857|gb|EU TATGCTATCCTACGATCTATAGCTAACAAGTTGGGAGGTGTCCTTGCGCT 881

T_tinca1_gi|325169898|gb|HM167 TACGCCATTTTACGATCAATCCCCAACAAGCTAGGAGGTGTTCTTGCACT 881

T_tinca2_gi|444488022|gb|JX974 TACGCCATTTTACGATCAATCCCAAACAAGCTAGGAGGTGTTCTTGCACT 888

G_gobio1_gi|37904500|gb|AY4265 TACGCCATTCTACGATCTATCCCTAACAAATTAGGAGGCGTCCTTGCACT 881

B_barbus1_gi|462648905|gb|KC46 TATGCTATTTTACGGTCGATCCCGAACAAACTAGGAGGGGTTCTCGCTTT 881

C_carpio1_gi|68532783|dbj|AB15 TACGCCATCCTACGATCAATTCCAAACAAACTCGGAGGTGTCCTTGCACT 881

R_amarus1_gi|636792944|dbj|AB3 TACGCCATCTTACGATCTATCCCAAACAAACTTGGAGGTGTCCTAGCACT 881

B_barbatula2_gi|73671964|gb|DQ TATGCCATCCTGCGATCGATCCCAAACAAGCTAGGGGGCGTGCTAGCCCT 881

C_taenia1_gi|62912083|gb|AY940 TACGCTATCCTTCGATCAATCCCCAATAAACTAGGAGGAGTTCTAGCCTT 867

M_fossilis1_gi|117959984|gb|DQ TATGCCATCCTGCGGTCAATCCCCAATAAATTAGGTGGAGTCCTGGCCCT 881

A_anguilla1_gi|159461951|gb|EU TACGCCATTCTACGATCAATTCCTAATAAACTAGGCGGGGTATTAGCCTT 881

T_thymallus_gi|408833805|gb|JX TACGCAATCCTACGATCAATCCCCAATAAACTAGGAGGAGTTCTTGCACT 881

S_trutta1_gi|1001096|dbj|D5840 TACGCAATCCTTCGCTCCATTCCTAATAAACTAGGCGGAGTACTCGCCCT 881

E_lucius1_gi|325610655|gb|HM17 TATGCGATCCTACGATCAATTCCTAACAAACTAGGAGGAGTCCTTGCCCT 881

L_lota1_gi|77386019|gb|DQ17405 TACGCAATTTTACGATCTATTCCTAACAAACTAGGCGGAGTTCTTGCCCT 881

L_planeri1_gi|261824331|gb|GQ2 TATGCGATTCTACGATCTATTCCTAATAAATTAGGTGGGGTCATAGCTTT 893

NM_gi|671723846|gb|KF549990.1 GCTCGCCTCCATCTTAGTTTTAATACTAGTCCCCCTACTCCACACCTCTA 895

NM_gi|168485493|gb|EU331236.1 GCTCGCCTCCATCCTAGTTTTAATACTAGTCCCCTTACTCCACACCTCTA 931

NF_gi|257220486|gb|GQ444442.1 ACTTGCCTCTATTTTAGTTCTGATACTAGTACCTTTCCTCCACACCTCTA 931

NF_gi|671723852|gb|KF549993.1 GCTTGCCTCCATCTTAGTTTTGATGCTTGTGCCTTTTCTCCACACCTCTA 895

BG_gi|526850801|gb|KF415509.1 ACTCGCCTCCATCCTAATTTTAATACTAGTGCCATTTCTTCATACCTCCA 931

BG_gi|188988554|gb|EU444667.1 ACTCGCCTCCATCCTAATTTTAATACTAGTGCCATTTCTTCATACCTCCA 931

PK_gi|589911366|gb|KC886260.1 ACTCGCCTCCATTTTGATTCTAATACTGGTACCATTTCTCCACACCTCTA 907

PM_gi|526851063|gb|KF415640.1 ACTAGCCTCTATCTTAATCTTAATACTAGTGCCCTTTCTTCATACCTCTA 931

PM_gi|188988552|gb|EU444666.1 GCTCGCTTCTATTTTAATCTTATTATTAGTGCCCTTTCTTCACACCTCTA 931

G_niger_gi|526850949|gb|KF4155 TCTGGCCTCTATTTTGGTCCTTTTAGTCGTCCCCTTCCTTCACACTTCTA 931

P_minutus_gi|237638806|gb|FJ52 ACTAGCCTCCATTCTTGTACTACTCCTAGTACCATTCCTCCACACCTCGA 931

GC3_gi|20339610|gb|AF386598.1 ACTAGCCTCAATTCTTATCCTTATGGCTGTCCCAATTCTTCACACCTCCA 931

PF3_gi|164562184|gb|EU348846.1 ACTTGCCTCCATCCTGGTTCTTATAGTTGTCCCCATCCTTCACACTTCTA 931

CG1_gi|27762429|gb|AY116366.1 CCTGGCATCCATCCTGGTACTCATAGTAGTACCCATCCTCCACACATCAA 931

GA1_gi|25140372|gb|AY116004.1 CCTTGCCTCCATCCTAGTCTTGATAGTAGTCCCCATCCTTCATACATCTA 931

L_cephalus1_gi|12657413|emb|AJ ACTATTCAGTATCCTAGTGCTACTAGTTGTGCCAATCTTACACACCTCAA 931

L_cephalus2_gi|41057843|gb|AY5 ACTATTCAGCATCCTAGTGCTAATAGTTGTGCCAATCTTACACACCTCTA 931

L_cepahlus3_gi|269997201|gb|GU ACTATTCAGCATCCTAGTGCTAATAGTTGTGCCAATCTTACACACCTCTA 931

C_nasus1_gi|18460951|gb|AY0264 ACTATTCAGCATCCTAGTGCTATTAGTCGTGCCAATTTTACACACATCCA 931

L_souffia1_gi|29691926|emb|AJ5 ACTGTTCAGCATCCTGGTGCTAATAGTCGTGCCAATTTTACACACGTCCA 909

R_rutilus1_gi|207113302|gb|FJ0 ACTATTCAGCATTCTAGTGCTAATAGTTGTCCCAGTCTTACACACCTCAA 931

B_bjoerkna1_gi|133872335|gb|EF ATTATTCAGTATTTTAGTGCTAATAGTTGTGCCAATCTTACATACCTCAA 931

A_brama2_gi|549466030|gb|KF552 --------------------------------------------------

B_bjoerkna2_gi|2281719|emb|Y10 ATTATTCAGTATTTTAGTGCTAATAGTTGTGCCAATCTTACATACCTCAA 931

L_leuciscus1_gi|307090186|gb|H ACTATTTAGCATTCTTGTGCTAATAGTTGTGCCAATTTTACACACCTCAA 931

L_leuciscus3_gi|2281727|emb|Y1 ACTATTTAGCATCCTTGTGCTAATAGTCGTGCCAATTTTACACACCTCAA 931

A_alburnus1_gi|386367699|gb|JQ ACTGTTTAGTATTCTAGTGCTAATAGTTGTGCCAATTCTACATACCTCAA 931

A_alburnus2_gi|85679841|gb|DQ3 ATTATTTAGTATTCTAGTGCTAATAGTTGTGCCAATTCTACACACCTCAA 916

L_delineatus1_gi|2281725|emb|Y ATTATTCAGTATTCTGGTGCTAATAGTTGTGCCGATTATACATACCTCAA 931

S_erythrophthalmus1_gi|2140109 ATTATTTAGCATCCTAGTGCTAATAGTTGTGCCAATTCTACATACCTCAA 931

A_bipunctatus2_gi|2281713|emb| GCTATTTAGCATCTTGGTGCTAATAGTCGTGCCAATTCTACACACCTCAA 931

A_bipunctatus_gi|5832982|gb|AF ACTATTTAGCATCTTGGTATTAATGGTCGTGCCAGTCTTACACACCTCAA 931

P_phoxinus1_gi|209968201|gb|EU ATTATTTAGTATCTTGGTATTAATAGTCGTCCCTACTTTACACACCTCAA 931

P_phoxinus2_gi|168203857|gb|EU ATTATTTAGTATCTTGGTCCTGATAGTCGTCCCTATTTTACACACCTCCA 931

T_tinca1_gi|325169898|gb|HM167 ATTATTCTCTATTTTAGTACTAATGGTGGTACCAATCTTACATACCTCAA 931

T_tinca2_gi|444488022|gb|JX974 ATTATTCTCTATTTTAGTACTAATGGTGGTACCGATCTTACATACCTCAA 938

G_gobio1_gi|37904500|gb|AY4265 ATTGTTTTCTATTCTGATTCTCATGGTGGTCCCAATTTTACACACCTCGA 931

B_barbus1_gi|462648905|gb|KC46 ACTATTCTCCATTCTAGTACTAATAGTGGTACCGCTCCTACACACCTCAA 931

C_carpio1_gi|68532783|dbj|AB15 CCTATTCTCCATTCTGGTATTAATAGTAGTACCACTACTACACACCTCAA 931

R_amarus1_gi|636792944|dbj|AB3 ATTATTTTCTATTCTTGTACTTATAGTAGTGCCACTGCTCCATACATCCA 931

B_barbatula2_gi|73671964|gb|DQ CCTCTTTTCCATCCTAATCCTAATGATTGTACCCATTCTTCACACGTCAA 931

C_taenia1_gi|62912083|gb|AY940 ATTATTTTCAATCCTAGTTTTAATAGTAGTACCTATTCTTCATACGTCAA 917

M_fossilis1_gi|117959984|gb|DQ ATTATTCTCTATCCTGGTCTTAATAGTAGTCCCCATCTTACACACCTCAA 931

A_anguilla1_gi|159461951|gb|EU GTTATCCTCCATCCTAGTTCTAATAGTAGTACCAATTCTTCACACCTCAA 931

T_thymallus_gi|408833805|gb|JX ACTATTCTCTATTCTGGTCCTAATAGTTGTACCCATCCTTCACACCTCTA 931

S_trutta1_gi|1001096|dbj|D5840 CTTATTCTCGATCCTGGTCCTTATAGTCGTTCCTATCCTCCATACCTCTA 931

E_lucius1_gi|325610655|gb|HM17 ACTATTTTCAATCCTGATCCTAATACTAGTACCCATCCTCCACACCTCTA 931

L_lota1_gi|77386019|gb|DQ17405 TCTATTTTCAATTCTAATCCTGATAGTTGTACCCTTCCTACACACCTCCA 931

L_planeri1_gi|261824331|gb|GQ2 AGCAGCAGCCATCATAATCCTCCTGGTTATCCCCTTTACCCACACCTCTA 943

NM_gi|671723846|gb|KF549990.1 AGCACCGGAGCCTCACCTTCCGCCCACTCTCACAATTCTTATTTTGAACA 945

NM_gi|168485493|gb|EU331236.1 AGCACCGGAGCCTTACCTTCCGCCCACTCTCACAATTCTTATTTTGAACA 981

NF_gi|257220486|gb|GQ444442.1 AGCACCGAAGCCTCACCTTCCGCCCACTTTCACAATTCCTATTTTGGATA 981

NF_gi|671723852|gb|KF549993.1 AACACCGAAGCCTCACCTTCCGCCCACTCTCACAATTCCTATTCTGAACA 945

BG_gi|526850801|gb|KF415509.1 AACATCGAAGCCTCACCTTTCGTCCACTCTCTCAACTACTATTCTGAACG 981

BG_gi|188988554|gb|EU444667.1 AACATCGAAGCCTCACCTTTCGTCCACTCTCTCAACTACTATTCTGAACG 981

PK_gi|589911366|gb|KC886260.1 AACACCGGAGCCTCACCTTTCGTCCATTTTCTCAACTACTGTTCTGAATA 957

PM_gi|526851063|gb|KF415640.1 AACATCGCAGCCTCACCTTTCGCCCATTTTCCCAACTATTATTCTGAACA 981

PM_gi|188988552|gb|EU444666.1 AACATCGAGGCCTCACCTTTCGCCCGTTTTCCCAAATACTATTCTGAACA 981

G_niger_gi|526850949|gb|KF4155 AGCAGCGGGGGCTCACCTTCCGTCCTCTTTCCCAGCTTTTATTCTGAGTC 981

P_minutus_gi|237638806|gb|FJ52 AGCAACGAGGGCTTACCTTCCGCCCCCTTTCGCAGTTCTTGTTCTGAACA 981

GC3_gi|20339610|gb|AF386598.1 AACAACGAGGCATCACTTTCCGACCGCTCTCACAGTTCCTCTTTTGAACG 981

PF3_gi|164562184|gb|EU348846.1 AACAACGTGGCATTACATTCCGACCGCTCTCCCAATTCCTCTTTTGAACA 981

CG1_gi|27762429|gb|AY116366.1 AACAACGAGGCCTAACCTTTCGCCCCATGACCCAATTTCTATTTTGAACA 981

GA1_gi|25140372|gb|AY116004.1 AACAACGTGGCCTTACATTTCGACCCCTTACGCAATTTTTATTTTGAACT 981

L_cephalus1_gi|12657413|emb|AJ AACAACGAGGACTAACTTTCCGCCCCGTAACTCAATTCCTATTCTGAACC 981

L_cephalus2_gi|41057843|gb|AY5 AACAACGAGGACTAACTTTCCGCCCCGTAACTCAATTCCTATTCTGAACC 981

L_cepahlus3_gi|269997201|gb|GU AACAACGAGGACTAACTTTCCGCCCCGTAACTCAATTCCTATTCTGAACC 981

C_nasus1_gi|18460951|gb|AY0264 AACAACGAGGACTAACTTTCCGCCCAGTGACCCAATTCCTATTCTGAACC 981

L_souffia1_gi|29691926|emb|AJ5 AACAA--------------------------------------------- 914

R_rutilus1_gi|207113302|gb|FJ0 AACAACGAGGACTAACTTTCCGCCCTGTGACACAATTTTTATTCTGAACC 981

B_bjoerkna1_gi|133872335|gb|EF AGCAACGAGGACTAACTTTCCGTCCTATAACACAATTCTTATTCTGAACC 981

A_brama2_gi|549466030|gb|KF552 --------------------------------------------------

B_bjoerkna2_gi|2281719|emb|Y10 AACAACGAGGACTAACTTTCCGCCCTATAACGCAATTCTTATTTTGAACC 981

L_leuciscus1_gi|307090186|gb|H AACAACGAGGACTAACTTTTCGACCTGTAACCCAATTCCTATTCTGAACC 981

L_leuciscus3_gi|2281727|emb|Y1 AGCAACGAGGACTAACTTTTCGACCTGTGACCCAATTCCTATTCTGAACC 981

A_alburnus1_gi|386367699|gb|JQ AACAACGAGGACTAACTTTCCGCCCCGTGACACAATTCCTATTTTGAACC 981

A_alburnus2_gi|85679841|gb|DQ3 AACAACGAGGACTAACTTTCCGCCCCGTGACGCAATTCCTATTTTGAACC 966

L_delineatus1_gi|2281725|emb|Y AACAACGAGGACTAACCTTCCGTCCCGTGACCCAATTCCTATTCTGAACC 981

S_erythrophthalmus1_gi|2140109 AGCAGCGAGGACTAACTTTCCGCCCCGTGACTCAATTTTTATTCTGAACC 981

A_bipunctatus2_gi|2281713|emb| AACAACGGGGACTAACTTTCCGCCCGCTAACACAATTCCTATTCTGAACC 981

A_bipunctatus_gi|5832982|gb|AF AACAACGAGGACTAACTTTCCGCCCTTTGACACAATGCCTATTCTGAACC 981

P_phoxinus1_gi|209968201|gb|EU AACAACGAGGATTAACTTTCCGCCCATTAACCCAATTCTTATTCTGAACC 981

P_phoxinus2_gi|168203857|gb|EU AGCAACGAGGACTAACTTTCCGCCCATTAACCCAATTTTTATTCTGAACC 981

T_tinca1_gi|325169898|gb|HM167 AACAACGAGGACTTACATTCCGCCCAATCACTCAATTCTTATTCTGAACC 981

T_tinca2_gi|444488022|gb|JX974 AACAACGAGGACTTACATTCCGCCCAATCACTCAATTCTTATTCTGAACC 988

G_gobio1_gi|37904500|gb|AY4265 AACAACGAGGACTAACCTTCCGCCCCCTAACACAATTCTTATTCTGAACC 981

B_barbus1_gi|462648905|gb|KC46 AACAACGGGGACTAACCTTCCGCCCAATCACCCAGTTCCTGTTCTGAACC 981

C_carpio1_gi|68532783|dbj|AB15 AACAACGAGGACTAACATTCCGCCCCATCACCCAATTCCTATTCTGAACC 981

R_amarus1_gi|636792944|dbj|AB3 AACAACGCGGACTAACCTACCGTCCAATCACTCAATTCTTATTTTGAACA 981

B_barbatula2_gi|73671964|gb|DQ AACAGCGAGGGCTGACATTCCGACCAATTACTCAACTCCTCTTCTGAACC 981

C_taenia1_gi|62912083|gb|AY940 AACAACGAGGCCTAGCATTCCGACCAATCACTCAATTCCTCTTTTGAACC 967

M_fossilis1_gi|117959984|gb|DQ AACAACGAAGCTTAACATTCCGACCAATCACCCAATTTCTCTTTTGAGCC 981

A_anguilla1_gi|159461951|gb|EU AACAACGAGGACTTACATTCCGACCTGCCTCCCAACTACTATTCTGAATT 981

T_thymallus_gi|408833805|gb|JX AACAACGAGGCCTAACTTTCCGACCACTCACCCAATTCTTGTTCTGAACC 981

S_trutta1_gi|1001096|dbj|D5840 AGCAACGCGGACTAACCTTTCGCCCCCTAACCCAATTCTTATTCTGAACC 981

E_lucius1_gi|325610655|gb|HM17 AGCAGCGCGGAATCACTTTCCGCCCCCTTACCCAATTACTATTTTGACTC 981

L_lota1_gi|77386019|gb|DQ17405 AACAACGAGGCTTGACATTCCGCCCACTCACTCAAGCACTGTTTTGAGTT 981

L_planeri1_gi|261824331|gb|GQ2 AACAACGAGGCATTCAATTTCGTCCGCTCGCCCAAGTTACATTTTGGATT 993

NM_gi|671723846|gb|KF549990.1 CTAGTGGCAGACGTAATAATCCTAACCTGAATTGGCGGCATACCAGTAGA 995

NM_gi|168485493|gb|EU331236.1 CTAGTGGCAGACGTAATAATCCTAACCTGAATTGGCGGCATACCAGTAGA 1031

NF_gi|257220486|gb|GQ444442.1 CTAGTGGCAGACGTAATGATCCTAACCTGAATTGGTGGCATACCAGTTGA 1031

NF_gi|671723852|gb|KF549993.1 CTAGTAGCAGACGTAGTCATCCTAACCTGAATTGGCGGCATGCCAGTCGA 995

BG_gi|526850801|gb|KF415509.1 CTAGTAGCAGATGTAGCAATCCTTACCTGAATTGGGGGAATACCAGTAGA 1031

BG_gi|188988554|gb|EU444667.1 CTAGTAGCAGATGTAGCAATCCTTACCTGAATCGGGGGAATACCAGTAGA 1031

PK_gi|589911366|gb|KC886260.1 CTAGTAGCAGATGTAGCAATCCTCACCTGAATTGGAGGAATGCCAGTAGA 1007

PM_gi|526851063|gb|KF415640.1 CTAGTTGCAGACGTAGCAATCCTTACCTGAATTGGAGGCATACCAGTAGA 1031

PM_gi|188988552|gb|EU444666.1 CTAGTAGCAGATGTGGCAATCCTTACCTGAATTGGGGGGATGCCAGTAGA 1031

G_niger_gi|526850949|gb|KF4155 TTAGTGGCCGACGTCATCATCCTCACCTGAATCGGCGGCATACCCGTCGA 1031

P_minutus_gi|237638806|gb|FJ52 CTCGTGGCTGATGTGCTCATTCTAACCTGAATCGGTGGCATGCCGGTCGA 1031

GC3_gi|20339610|gb|AF386598.1 CTAATTGCAGACGTTGCTATCCTCACCTGAATTGGAGGAATACCAGTCGA 1031

PF3_gi|164562184|gb|EU348846.1 TTAATCGCAGATGTTGTTATCCTTACCTGAATTGGAGGAATACCTGTAGA 1031

CG1_gi|27762429|gb|AY116366.1 CTCATCGCAGACGTCGCCATTCTCACATGAATTGGAGGCATGCCCGTCGA 1031

GA1_gi|25140372|gb|AY116004.1 CTTATTGCAGATGTAGCCATTCTTACCTGAATTGGCGGTATACCCGTTGA 1031

L_cephalus1_gi|12657413|emb|AJ CTAGTTGCAGATATATTTATCCTGACATGAATTGGGGGCATACCTGTAGA 1031

L_cephalus2_gi|41057843|gb|AY5 CTAGTTGCAGATATATTTATCCTGACATGAATTGGGGGCATACCTGTAGA 1031

L_cepahlus3_gi|269997201|gb|GU CTAGTTGCAGATATATTTATCCTGACATGAATTGGGGGCATACCTGTAGA 1031

C_nasus1_gi|18460951|gb|AY0264 CTGGTTGCAGATATATTTATTTTGACATGAATCGGGGGTATACCCGTAGA 1031

L_souffia1_gi|29691926|emb|AJ5 --------------------------------------------------

R_rutilus1_gi|207113302|gb|FJ0 CTAGTTGCAGATATATTTATCTTAACATGAATCGGAGGCATGCCCGTAGA 1031

B_bjoerkna1_gi|133872335|gb|EF CTAGTTGCAGACATGGTCATTCTAACATGAATTGGAGGCATACCCGTAGA 1031

A_brama2_gi|549466030|gb|KF552 --------------------------------------------------

B_bjoerkna2_gi|2281719|emb|Y10 TTAGTTGCAGACATAATCATCCTGACATGAATCGGAGGCATACCCGTAGA 1031

L_leuciscus1_gi|307090186|gb|H TTAATTGCAGATATAATTATCTTGACATGAATTGGGGGCATACCCGTAGA 1031

L_leuciscus3_gi|2281727|emb|Y1 TTAATTGCAGATATAATTATCTTAACATGAATTGGAGGCATACCCGTAGA 1031

A_alburnus1_gi|386367699|gb|JQ CTAGTCGCAGATATGATTATCTTAACATGAATTGGGGGCATGCCTGTAGA 1031

A_alburnus2_gi|85679841|gb|DQ3 CTAGTCGCAGATATGATTATCTTAACATGAATTGGGGGCATGCCCGTAGA 1016

L_delineatus1_gi|2281725|emb|Y CTAGGTGCAGATATGATCATCTTGACATGAATTGGAGGCATACCCGTAGA 1031

S_erythrophthalmus1_gi|2140109 CTGGTTGCAGACATAATTATCCTAACATGAATTGGGGGCATGCCCGTAGA 1031

A_bipunctatus2_gi|2281713|emb| CTAGTTGCAGATATAATTATTTTAACATGAATTGGGGGCATACCTGTAGA 1031

A_bipunctatus_gi|5832982|gb|AF CTCGTTGCAGATATGATTATCTTAACATGAATTGGGGGCATACCCGTAGA 1031

P_phoxinus1_gi|209968201|gb|EU CTACTAGCAGATATAGTCATTTTGACATGAATTGGGGGCATACCCGTAGA 1031

P_phoxinus2_gi|168203857|gb|EU CTTGTAGCAGATATGCTTATTTTAACATGAATTGGAGGCATACCTGTAGA 1031

T_tinca1_gi|325169898|gb|HM167 TTGGTAGCAGATATAGTAATCTTAACATGAATCGGTGGTATACCTGTAGA 1031

T_tinca2_gi|444488022|gb|JX974 TTGGTAGCGGATATAGTAATCTTAACATGAATCGGCGGTATACCTGTAGA 1038

G_gobio1_gi|37904500|gb|AY4265 CTCGTAGCAGACATACTTATCCTAACATGAATTGGAGGCATGCCGGTAGA 1031

B_barbus1_gi|462648905|gb|KC46 CTAGTAGCAGATATAATTATCTTAACATGAATCGGGGGTATACCAGTAGA 1031

C_carpio1_gi|68532783|dbj|AB15 CTAGTAGCGGACATAATTATCCTAACATGAATTGGAGGCATACCAGTAGA 1031

R_amarus1_gi|636792944|dbj|AB3 TTAGTGGCAGATATAATTATTCTGACATGGATTGGAGGCATACCCGTAGA 1031

B_barbatula2_gi|73671964|gb|DQ TTGGTCGCGGACATACTTATTTTAACGTGAATCGGGGGCATACCCGTAGA 1031

C_taenia1_gi|62912083|gb|AY940 CTAGTTGCAGACATACTTATCCTTACATGAATTGGAGGAATACCAGTAGA 1017

M_fossilis1_gi|117959984|gb|DQ TTAGTGGCAGACATACTTATCCTAACATGAATTGGGGGCATGCCGGTAGA 1031

A_anguilla1_gi|159461951|gb|EU TTAGTAGCAGATATACTAGTACTAACATGAATCGGAGGAATACCAGTAGA 1031

T_thymallus_gi|408833805|gb|JX CTAGTCGCAGACATACTCATCCTCACCTGAATTGGAGGCATACCTGTAGA 1031

S_trutta1_gi|1001096|dbj|D5840 CTAGTAGCAGACATACTGATCCTCACCTGAATTGGGGGGATACCTGTAGA 1031

E_lucius1_gi|325610655|gb|HM17 TTGGTAGCAGATATATTAATCTTAACATGAATTGGAGGAATACCCGTTGA 1031

L_lota1_gi|77386019|gb|DQ17405 CTCGTCGCAGACATACTTGTCTTAACATGAATTGGCGGGGTACCAGTAGA 1031

L_planeri1_gi|261824331|gb|GQ2 CTGATTGCTGATCTAGCGCTACTCACTTGACTAGGGGGAGAACCAGCTGA 1043

NM_gi|671723846|gb|KF549990.1 AGACCCCTATGTGATGATCGGACAGCTAGCCTCAATATTGTACTTTTCCA 1045

NM_gi|168485493|gb|EU331236.1 AGACCCCTATGTAATAATCGGACAGCTAGCCTCAATATTGTACTTTTCCA 1081

NF_gi|257220486|gb|GQ444442.1 GGATCCATATGTTTTAATCGGACAAGTCGCCTCTGTATTATACTTCTCCA 1081

NF_gi|671723852|gb|KF549993.1 AGACCCATATGTTTTAATCGGACAACTAGCCTCTGTTTTATACTTCTCCA 1045

BG_gi|526850801|gb|KF415509.1 ACACCCCTATATTATTATTGGTCAAATTGCTTCAGTGCTATACTTTTCCA 1081

BG_gi|188988554|gb|EU444667.1 ACACCCCTATATTATTATTGGTCAAATTGCTTCAGTGCTATACTTTTCCA 1081

PK_gi|589911366|gb|KC886260.1 ACACCCCTATATCATCATCGGTCAAATTGCTTCAGTGCTATACTTCTCAA 1057

PM_gi|526851063|gb|KF415640.1 ACATCCTTATATTATTATTGGGCAAATTGCCTCAGTACTATACTTTTCCC 1081

PM_gi|188988552|gb|EU444666.1 ACACCCCTATATTATTATCGGACAAATTGCCTCAGCACTTTACTTTTCTA 1081

G_niger_gi|526850949|gb|KF4155 GCACCCCTATGTTATCATTGGACAAATCGCATCCGTATTGTACTTCTCCA 1081

P_minutus_gi|237638806|gb|FJ52 GCACCCCTACATCATCATCGGACAGCTCGCGTCCCTCCTCTACTTCTCCA 1081

GC3_gi|20339610|gb|AF386598.1 ACACCCTTTCATCATCATTGGCCAAATCGCATCTTTCCTATATTTCTTCC 1081

PF3_gi|164562184|gb|EU348846.1 ACACCCCTTCATCATTATTGGCCAAGTCGCATCTTTCTTGTACTTCTCCC 1081

CG1_gi|27762429|gb|AY116366.1 ACACCCCTTTATTATCATTGGCCAAATTGCATCTCTCCTATACTTCTTTC 1081

GA1_gi|25140372|gb|AY116004.1 ACACCCCTTCATCATTATTGGACAAGTTGCATCCGTACTTTACTTCTCTT 1081

L_cephalus1_gi|12657413|emb|AJ ACACCCATATATTATCATTGGCCAAGTCGCATCCATTCTATACTTTGCGC 1081

L_cephalus2_gi|41057843|gb|AY5 ACACCCATATATTATCATTGGCCAAGTCGCATCCATTCTATATTTTGCAC 1081

L_cepahlus3_gi|269997201|gb|GU ACACCCATACATTATCATTGGCCAAGTCGCATCCATTCTATATTTTGCAC 1081

C_nasus1_gi|18460951|gb|AY0264 ACACCCATACATTGTTATTGGCCAAGTCGCATCCATCCTATACTTTGCAC 1081

L_souffia1_gi|29691926|emb|AJ5 --------------------------------------------------

R_rutilus1_gi|207113302|gb|FJ0 ACACCCATATATCATTATTGGCCAAGTTGCATCCATTCTATACTTTGCAC 1081

B_bjoerkna1_gi|133872335|gb|EF ACACCCATATATTATTATTGGCCAAGTCGCATCCATTCTATACTTTGCAC 1081

A_brama2_gi|549466030|gb|KF552 --------------------------------------------------

B_bjoerkna2_gi|2281719|emb|Y10 ACACCCATATATTATTATTGGCCAAGTCGCATCCATTTTATATTTTGCAC 1081

L_leuciscus1_gi|307090186|gb|H ACACCCATATGTCATTATTGGCCAAGTCGCATCCGTTCTATACTTTGCAC 1081

L_leuciscus3_gi|2281727|emb|Y1 GCACCCATATGTCATTATTGGCCAAATCGCATTCGTTCTATACTTTGCAC 1081

A_alburnus1_gi|386367699|gb|JQ GCACCCATACATTATTATTGGTCAGGTCGCATCCGTCCTATACTTTGCAC 1081

A_alburnus2_gi|85679841|gb|DQ3 GCACCCATACATTATTATTGGTCAGGTCGCATCCGTCCTATACTTTGCAC 1066

L_delineatus1_gi|2281725|emb|Y GCACCCGTATGTTATTATCGGTCAAGGCGCATCCATCCTATACTTTGCAC 1081

S_erythrophthalmus1_gi|2140109 GCACCCGTACATTGTTATTGGTCAAGTCGCATCCATCTTATACTTTGCAC 1081

A_bipunctatus2_gi|2281713|emb| GCACCCGTACATTATTATTGGCCAAATTGCATCAATTTTATACTTTGCTC 1081

A_bipunctatus_gi|5832982|gb|AF GCACCCGTACATTATTATTGGCCAAATTGCATCAATTTTATACTTTGCAC 1081

P_phoxinus1_gi|209968201|gb|EU ACACCCATATATTATTATTGGTCAAGTAGCGTCAATTTTATACTTTGCAC 1081

P_phoxinus2_gi|168203857|gb|EU GCACCCATACATTATTATTGGCCAAGTAGCGTCGATTTTATACTTTGCAC 1081

T_tinca1_gi|325169898|gb|HM167 ACACCCATATATTATCATTGGTCAGATTGCATCAATTCTATACTTCGCAC 1081

T_tinca2_gi|444488022|gb|JX974 ACACCCATATATCATCATTGGTCAAATTGCATCAATTTTATACTTCGCAC 1088

G_gobio1_gi|37904500|gb|AY4265 ACATCCATATGTTGTCATCGGCCAAGTCGCTTCAATTCTGTATTTTGCAC 1081

B_barbus1_gi|462648905|gb|KC46 ACACCCATTCATCATCATCGGACAAATCGCATCCGTCCTATACTTCGCAC 1081

C_carpio1_gi|68532783|dbj|AB15 ACATCCCTTCATCATTATTGGACAAATTGCATCCGTCCTATACTTCGCAC 1081

R_amarus1_gi|636792944|dbj|AB3 GCACCCATACGTTATTATTGGGCAAATTGCGTCAGTGCTGTACTTCGCAC 1081

B_barbatula2_gi|73671964|gb|DQ ACACCCCTTTATTATCATCGGGCAAATCGCATCCGCCCTATATTTTGCGC 1081

C_taenia1_gi|62912083|gb|AY940 ACACCCATTCATTATTATTGGACAACTTGCGTCTGTTCTCTATTTTACAC 1067

M_fossilis1_gi|117959984|gb|DQ ACATCCATTTGTCATCATCGGGCAACTCGCATCCCTCCTATACTTTTTAT 1081

A_anguilla1_gi|159461951|gb|EU ACATCCGTACATTATCATCGGCCAAGTAGCATCAGTACTTTATTTTTCCC 1081

T_thymallus_gi|408833805|gb|JX ACACCCATTTATCATCATCGGTCAAGTCGCCTCTGTAATCTATTTCACTA 1081

S_trutta1_gi|1001096|dbj|D5840 ACACCCATTCATTATCATCGGTCAAGTTGCCTCTGTAATTTACTTTACTA 1081

E_lucius1_gi|325610655|gb|HM17 ACACCCCTTTATTATTATTGGACAAGTCACATCCGTAATTTATTTTGCCA 1081

L_lota1_gi|77386019|gb|DQ17405 ACACCCTTTCATCATCATTGGACAAGTAGCATCCGTCCTGTACTTCTCAT 1081

L_planeri1_gi|261824331|gb|GQ2 ACACCCATTTATTTTAATAACACAAATTGCATCAACAGTGTACTTTATAA 1093

NM_gi|671723846|gb|KF549990.1 TCTTTCTCGGCTTATTCCCCATAGTGAGCTGACTAGAGAACAAACTTTTA 1095

NM_gi|168485493|gb|EU331236.1 TCTTTCTCGGCTTATTCCCCATAGCGAGCTGACTAGAGAACAAACTTTTA 1131

NF_gi|257220486|gb|GQ444442.1 TCTTTCTCGGCCTATTCCCCATAGCGGGCTGGCTAGAGAACAAACTTTTA 1131

NF_gi|671723852|gb|KF549993.1 TCTTTCTCGTATTCTTCCCAATAGCCGGCTGACTAGAGAACAAACTTTTA 1095

BG_gi|526850801|gb|KF415509.1 TTTTTCTCGTCCTATTTCCTATTGTAGGCTGACTAGAAAATAAATTTTTA 1131

BG_gi|188988554|gb|EU444667.1 TTTTTCTCGTCCTATTTCCTATTGTAGGCTGACTAGAAAATAAATTTTTA 1131

PK_gi|589911366|gb|KC886260.1 TTTTTCTCGTCCTATTCCCTATTGTAGGCTGACTAGAAAATAAATTTTTA 1107

PM_gi|526851063|gb|KF415640.1 TCTTCCTAGTCCTATTTCCTATTGTAGGATGACTAGAAAACAAACTCTTA 1131

PM_gi|188988552|gb|EU444666.1 TTTTTCTTTTCCTATTTCCCGTTGTAGGATGACTAGAAAATAAACTTTTA 1131

G_niger_gi|526850949|gb|KF4155 TTTTTCTCCTCCTTTTCCCCACAGCTGGGTGGCTAGAGAACAAGATTCTA 1131

P_minutus_gi|237638806|gb|FJ52 TCTTCCTCGCCCTCTTTCCCCTCGCCGGGCTCGCGGAGAACAAACTTCTT 1131

GC3_gi|20339610|gb|AF386598.1 TATTCCTCGTTCTCGCCCCACTAGCAGGATGAGTCGAAAACAAGGCCCTC 1131

PF3_gi|164562184|gb|EU348846.1 TTTTCCTCGTCCTCACGCCACTAGCAGGATGGGCCGAAAACAAAGCCCTC 1131

CG1_gi|27762429|gb|AY116366.1 TTTTCTTAGCCTTATTCCCCCTAGCAGGCTGAATAGAAAACAAAGCCTTA 1131

GA1_gi|25140372|gb|AY116004.1 TATTCCTAGTTTTATACCCAGGAGCAGCAGTAATAGAGAACAAAATGCTT 1131

L_cephalus1_gi|12657413|emb|AJ TCTTCCTCGTCCTTGTCCCGCTAGCAGGATGAGTCGAAAATAAAGCACTG 1131

L_cephalus2_gi|41057843|gb|AY5 TCTTCCTCATCCTTGTCCCACTAGCAGGATGGGTGGAAAATAAAGCATTA 1131

L_cepahlus3_gi|269997201|gb|GU TCTTCCTCATCCTTGTCCCACTAGCAGGATGAGTGGAAAATAAAGCATTA 1131

C_nasus1_gi|18460951|gb|AY0264 TATTCCTCATTCTTGTCCCACTAGCAGGATGGGTGGAAAATAAAGCATTG 1131

L_souffia1_gi|29691926|emb|AJ5 --------------------------------------------------

R_rutilus1_gi|207113302|gb|FJ0 TTTTCCTCGTTCTTGTCCCGCTAGCAGGATGAGCGGAAAATAAAGCATTG 1131

B_bjoerkna1_gi|133872335|gb|EF TCTTCCTCATTCTTATTCCACTAGCAGGGTGAATGGAAAACAAAGCATTG 1131

A_brama2_gi|549466030|gb|KF552 --------------------------------------------------

B_bjoerkna2_gi|2281719|emb|Y10 TCTTCCTCATTCTTATCCCGCTAGCAGGATGAATAGAGAACAAAGCACTG 1131

L_leuciscus1_gi|307090186|gb|H TCTTCCTCGTTCTTGTCCCACTAGCAGGATGGGTCGAAAATAAAGCATTG 1131

L_leuciscus3_gi|2281727|emb|Y1 TCTTCCTCGTTCTTGTTCCACTAGCGGGGTGAGTCGAGAATAAAGCATTG 1131

A_alburnus1_gi|386367699|gb|JQ TCTTCCTTATCCTTATTCCACTAGCAGGGTTAATAGAGAATAAAGCATTG 1131

A_alburnus2_gi|85679841|gb|DQ3 TCTTCCTTATCCTT------------------------------------ 1080

L_delineatus1_gi|2281725|emb|Y TCTTCCTGATTCTTATCCCACTAGCAGGATTAATGGAAAATAAAGCATTG 1131

S_erythrophthalmus1_gi|2140109 TCTTCCTTATTCTTATTCCACTAGCAGGATTAATGGAAAATAAAGCATTG 1131

A_bipunctatus2_gi|2281713|emb| TTTTTCTCGTTCTTGCCCCACTAGCAGGATGAGTAGAAAATAAAGCACTG 1131

A_bipunctatus_gi|5832982|gb|AF TTTTTCTCGTTCTTGCCCCGCTAGCAGGATGAATAGAAAATAAAGCACTG 1131

P_phoxinus1_gi|209968201|gb|EU TCTTTCTCATCCTCACACCACTGGCAGGATGGCTAGAGAATAAGACATTA 1131

P_phoxinus2_gi|168203857|gb|EU TCTTTCTCATCCTCACACCACTGGCAGGATGGCTAGAGAATAAGACATTA 1131

T_tinca1_gi|325169898|gb|HM167 TTTTCCTTGTTTTTGCCCCCCTCGCAGGATGACTGGAAAATAAAGCACTG 1131

T_tinca2_gi|444488022|gb|JX974 TTTTCCTTGTTCTCGCCCCCCTCGCAGGATGACTGGAAAATAAAGCATTG 1138

G_gobio1_gi|37904500|gb|AY4265 TTTTCCTTGTCCTTGTCCCAATGGCAGGCTGACTGGAAAATAAAGCATTA 1131

B_barbus1_gi|462648905|gb|KC46 TATTCCTCATTCTCATTCCACTGGCAGGATGGTTAGAAAATAAAGCACTA 1131

C_carpio1_gi|68532783|dbj|AB15 TATTCCTCATTTTTATGCCACTAGCAGGATGGTTAGAAAATAAAGCACTA 1131

R_amarus1_gi|636792944|dbj|AB3 TTTTCCTCGTCCTCGCGCCATTAGCCGGATGACTAGAAAATAAAGCATTA 1131

B_barbatula2_gi|73671964|gb|DQ TATTCTTAGTCTTTATGCCAATGGCAGGATGACTAGAGAATAAAGCGCTA 1131

C_taenia1_gi|62912083|gb|AY940 TATTTCTAGTACTAATCCCACTGGCGGGATGACTAGAAAACAAGGCATTA 1117

M_fossilis1_gi|117959984|gb|DQ TATTCCTGGCTCTAATCCCACTGGCGGGATGGCTAGAAAACAAGGCAATA 1131

A_anguilla1_gi|159461951|gb|EU TATTCCTGGTGCTAAACCCCTTAGTCGGCTGACTAGAAAACAAAGTAATA 1131

T_thymallus_gi|408833805|gb|JX TCTTCCTAATTTTGGCCCCGCTAGCCGGATGAGCCGAAAACAAAGCCCTC 1131

S_trutta1_gi|1001096|dbj|D5840 TCTTCCTAGTTCTCGCCCCCTTGGCTGGCTGGGCTGAGAATAAAGCTCTT 1131

E_lucius1_gi|325610655|gb|HM17 TCTTCCTACTCCTCGCCCCCCTGGCCGGCTGATTAGAGAACAAAACCCTA 1131

L_lota1_gi|77386019|gb|DQ17405 TATTCCTAGTATTATTTCCCCTTGCAGGAATAACTGAAAATAAAGCCCTT 1131

L_planeri1_gi|261824331|gb|GQ2 TTTTTATTTTAATCTTTCCAATTCTAGGTCGCCTAGAAAA---------- 1133

NM_gi|671723846|gb|KF549990.1 TTA----------------------------------------------- 1098

NM_gi|168485493|gb|EU331236.1 TTAACATAACTGCACTAGTAGCTCAGCGCCAGAGCGCCGGCCTTGTAAGC 1181

NF_gi|257220486|gb|GQ444442.1 TTAACATAG----------------------------------------- 1140

NF_gi|671723852|gb|KF549993.1 TTA----------------------------------------------- 1098

BG_gi|526850801|gb|KF415509.1 TTAAACT------------------------------------------- 1138

BG_gi|188988554|gb|EU444667.1 TTAAACT------------------------------------------- 1138

PK_gi|589911366|gb|KC886260.1 TTAA---------------------------------------------- 1111

PM_gi|526851063|gb|KF415640.1 CTGAACT------------------------------------------- 1138

PM_gi|188988552|gb|EU444666.1 TTAAACT------------------------------------------- 1138

G_niger_gi|526850949|gb|KF4155 TTCAGCGCCT---------------------------------------- 1141

P_minutus_gi|237638806|gb|FJ52 AAACTAGCCT---------------------------------------- 1141

GC3_gi|20339610|gb|AF386598.1 GGATGATTC----------------------------------------- 1140

PF3_gi|164562184|gb|EU348846.1 GGA----------------------------------------------- 1134

CG1_gi|27762429|gb|AY116366.1 GGATGAACTT---------------------------------------- 1141

GA1_gi|25140372|gb|AY116004.1 GAATGAACA----------------------------------------- 1140

L_cephalus1_gi|12657413|emb|AJ AAATGAGCC----------------------------------------- 1140

L_cephalus2_gi|41057843|gb|AY5 AAATGAGCC----------------------------------------- 1140

L_cepahlus3_gi|269997201|gb|GU AAATGAGCCT---------------------------------------- 1141

C_nasus1_gi|18460951|gb|AY0264 AAATGAGCC----------------------------------------- 1140

L_souffia1_gi|29691926|emb|AJ5 --------------------------------------------------

R_rutilus1_gi|207113302|gb|FJ0 AAATGAGCC----------------------------------------- 1140

B_bjoerkna1_gi|133872335|gb|EF AAATGAGCCTGCCCTAGTAGCTTAGTTTTAAAGCATCGGT---------- 1171

A_brama2_gi|549466030|gb|KF552 --------------------------------------------------

B_bjoerkna2_gi|2281719|emb|Y10 AAATGAGCC----------------------------------------- 1140

L_leuciscus1_gi|307090186|gb|H AAATGAGCC----------------------------------------- 1140

L_leuciscus3_gi|2281727|emb|Y1 AAATGAGCC----------------------------------------- 1140

A_alburnus1_gi|386367699|gb|JQ AAATGAGCTT---------------------------------------- 1141

A_alburnus2_gi|85679841|gb|DQ3 --------------------------------------------------

L_delineatus1_gi|2281725|emb|Y AAATGAGCT----------------------------------------- 1140

S_erythrophthalmus1_gi|2140109 AAATGAGCC----------------------------------------- 1140

A_bipunctatus2_gi|2281713|emb| AAATGAGCC----------------------------------------- 1140

A_bipunctatus_gi|5832982|gb|AF AAATGAGCCT---------------------------------------- 1141

P_phoxinus1_gi|209968201|gb|EU AAATGAGCC----------------------------------------- 1140

P_phoxinus2_gi|168203857|gb|EU AAATGAGCC----------------------------------------- 1140

T_tinca1_gi|325169898|gb|HM167 AAATGAGCTT---------------------------------------- 1141

T_tinca2_gi|444488022|gb|JX974 AAATGAGC------------------------------------------ 1146

G_gobio1_gi|37904500|gb|AY4265 AAATGAGCTT---------------------------------------- 1141

B_barbus1_gi|462648905|gb|KC46 GAATGAGCTT---------------------------------------- 1141

C_carpio1_gi|68532783|dbj|AB15 AAATGAGCTT---------------------------------------- 1141

R_amarus1_gi|636792944|dbj|AB3 AAATGAGCTT---------------------------------------- 1141

B_barbatula2_gi|73671964|gb|DQ GAATGAGCT----------------------------------------- 1140

C_taenia1_gi|62912083|gb|AY940 GAATGAGCT----------------------------------------- 1126

M_fossilis1_gi|117959984|gb|DQ GAATGAGC------------------------------------------ 1139

A_anguilla1_gi|159461951|gb|EU AACTGATACG---------------------------------------- 1141

T_thymallus_gi|408833805|gb|JX GAATGAGCCT---------------------------------------- 1141

S_trutta1_gi|1001096|dbj|D5840 GAATGAACC----------------------------------------- 1140

E_lucius1_gi|325610655|gb|HM17 GAATAA-------------------------------------------- 1137

L_lota1_gi|77386019|gb|DQ17405 GAATGAAACT---------------------------------------- 1141

L_planeri1_gi|261824331|gb|GQ2 --------------------------------------------------

NM_gi|671723846|gb|KF549990.1 -----------------------

NM_gi|168485493|gb|EU331236.1 CGGACGCCGGAGGTTAAACCCCT 1204

NF_gi|257220486|gb|GQ444442.1 -----------------------

NF_gi|671723852|gb|KF549993.1 -----------------------

BG_gi|526850801|gb|KF415509.1 -----------------------

BG_gi|188988554|gb|EU444667.1 -----------------------

PK_gi|589911366|gb|KC886260.1 -----------------------

PM_gi|526851063|gb|KF415640.1 -----------------------

PM_gi|188988552|gb|EU444666.1 -----------------------

G_niger_gi|526850949|gb|KF4155 -----------------------

P_minutus_gi|237638806|gb|FJ52 -----------------------

GC3_gi|20339610|gb|AF386598.1 -----------------------

PF3_gi|164562184|gb|EU348846.1 -----------------------

CG1_gi|27762429|gb|AY116366.1 -----------------------

GA1_gi|25140372|gb|AY116004.1 -----------------------

L_cephalus1_gi|12657413|emb|AJ -----------------------

L_cephalus2_gi|41057843|gb|AY5 -----------------------

L_cepahlus3_gi|269997201|gb|GU -----------------------

C_nasus1_gi|18460951|gb|AY0264 -----------------------

L_souffia1_gi|29691926|emb|AJ5 -----------------------

R_rutilus1_gi|207113302|gb|FJ0 -----------------------

B_bjoerkna1_gi|133872335|gb|EF -----------------------

A_brama2_gi|549466030|gb|KF552 -----------------------

B_bjoerkna2_gi|2281719|emb|Y10 -----------------------

L_leuciscus1_gi|307090186|gb|H -----------------------

L_leuciscus3_gi|2281727|emb|Y1 -----------------------

A_alburnus1_gi|386367699|gb|JQ -----------------------

A_alburnus2_gi|85679841|gb|DQ3 -----------------------

L_delineatus1_gi|2281725|emb|Y -----------------------

S_erythrophthalmus1_gi|2140109 -----------------------

A_bipunctatus2_gi|2281713|emb| -----------------------

A_bipunctatus_gi|5832982|gb|AF -----------------------

P_phoxinus1_gi|209968201|gb|EU -----------------------

P_phoxinus2_gi|168203857|gb|EU -----------------------

T_tinca1_gi|325169898|gb|HM167 -----------------------

T_tinca2_gi|444488022|gb|JX974 -----------------------

G_gobio1_gi|37904500|gb|AY4265 -----------------------

B_barbus1_gi|462648905|gb|KC46 -----------------------

C_carpio1_gi|68532783|dbj|AB15 -----------------------

R_amarus1_gi|636792944|dbj|AB3 -----------------------

B_barbatula2_gi|73671964|gb|DQ -----------------------

C_taenia1_gi|62912083|gb|AY940 -----------------------

M_fossilis1_gi|117959984|gb|DQ -----------------------

A_anguilla1_gi|159461951|gb|EU -----------------------

T_thymallus_gi|408833805|gb|JX -----------------------

S_trutta1_gi|1001096|dbj|D5840 -----------------------

E_lucius1_gi|325610655|gb|HM17 -----------------------

L_lota1_gi|77386019|gb|DQ17405 -----------------------

L_planeri1_gi|261824331|gb|GQ2 -----------------------
