# Supplementary material for: An eDNA Assay to Monitor a Globally Invasive Fish Species from Flowing Freshwater
Source: PLoS One. 2016 Jan 27;11(1):e0147558. doi: 10.1371/journal.pone.0147558 (PMC4729461; doi:10.1371/journal.pone.0147558)

Modified eDNA sampling device

Materials as indicated in the technical drawings are stainless steel (1.4305), POM (Polyoxymethylene), and NBR O-ring's (Nitrile Rubber).

Additional items needed:

- screw nut M5
- screw nut M8
- spring dia. 14 -18mm, length 25 -35mm
- O-Ring 10 x 3mm
- O-Ring 30.2 x 3mm
- bowden cable 1.5mm

The center-hole in the screw-cap is 41mm wide.

The device is hand-operated by a bowden cable, which opens and closes the valve.
For opening and closing the valve, it is possible to adapt a handbrake from a bicycle, or to build a custom-made knob.

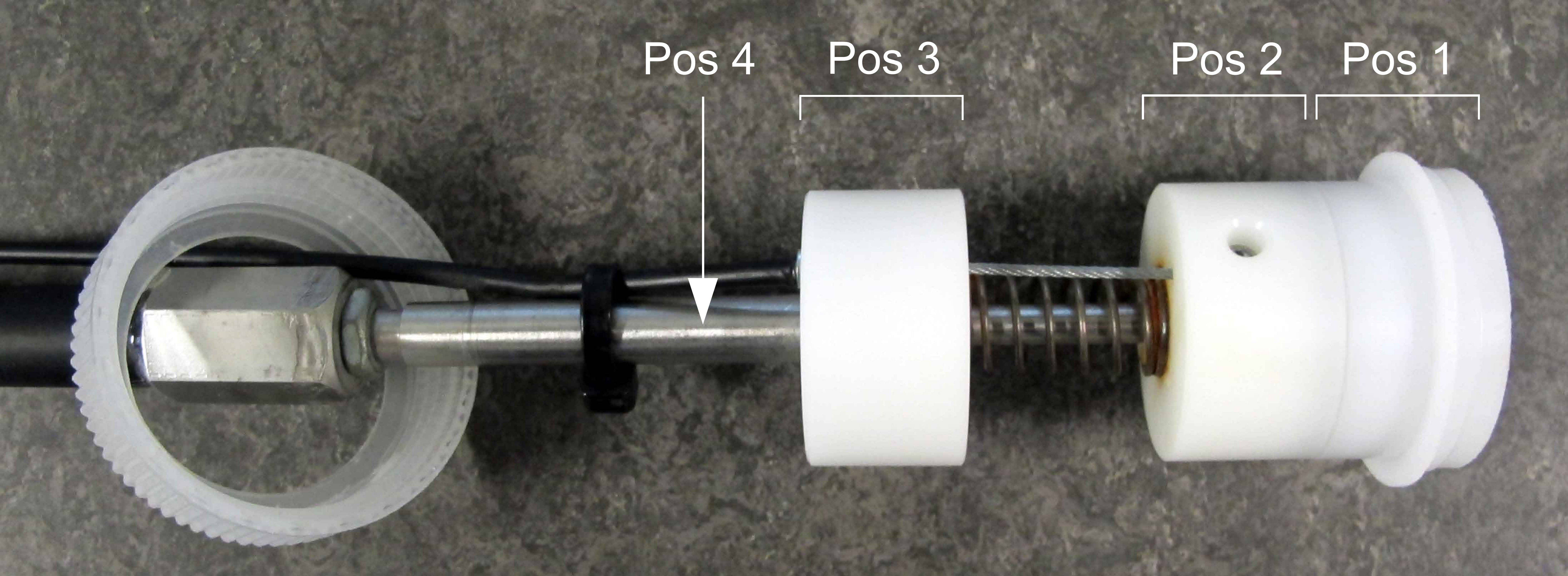
Pos 4
Pos 3
Pos 2
Pos 1

Pos. 4

Axle

1,4305

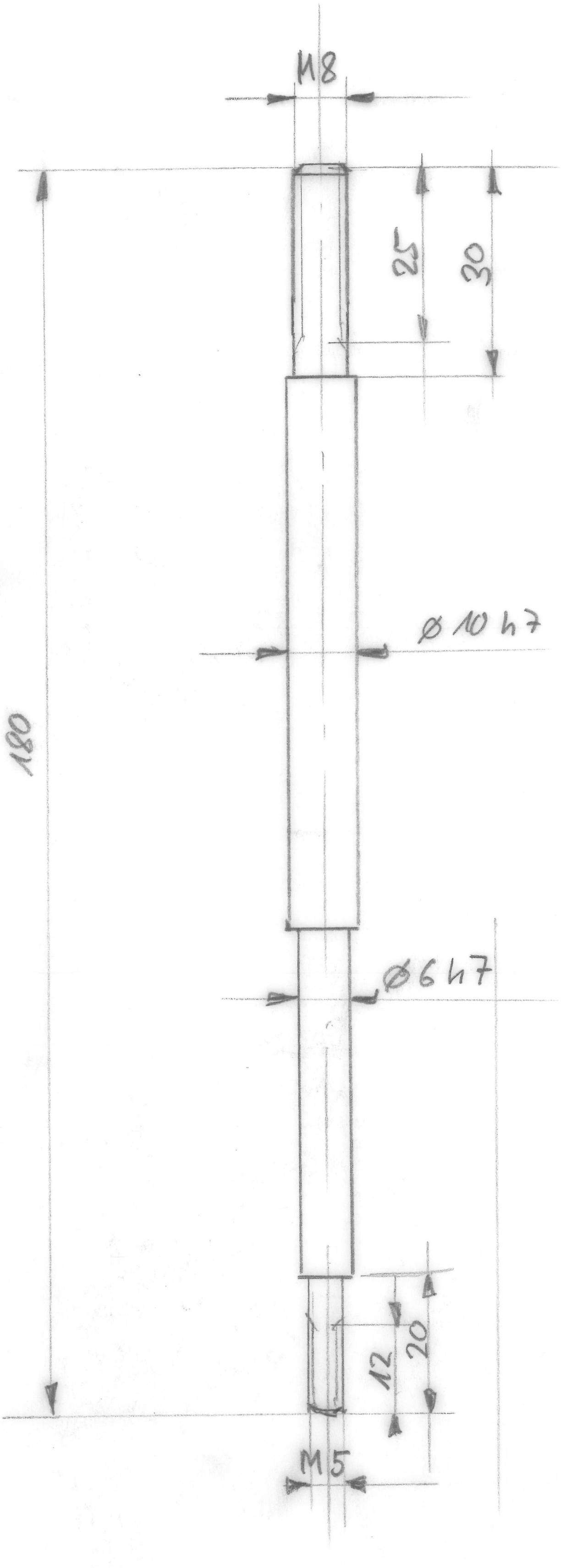

Pos. 2
Valve Cap
POM

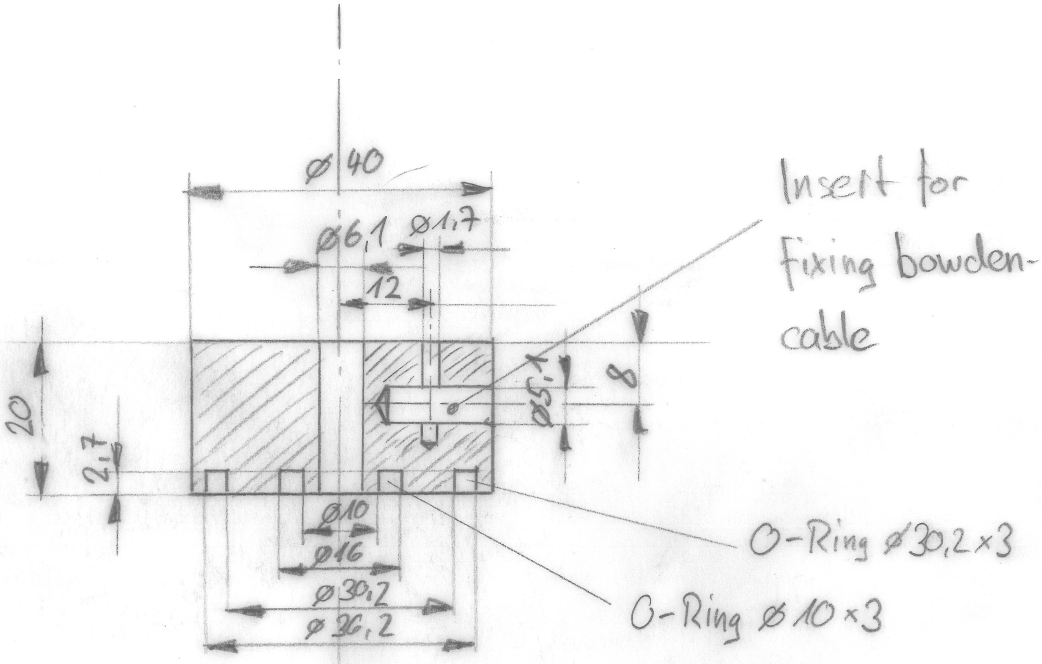

Pos. 1
Valve Body
POM

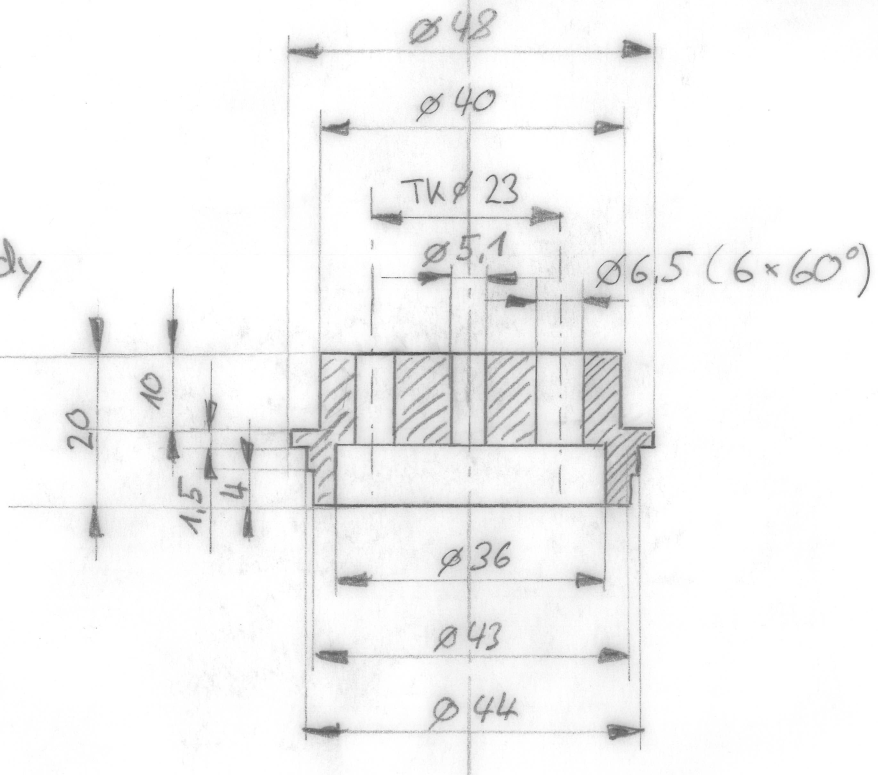

Pos. 3

Headplate

POM

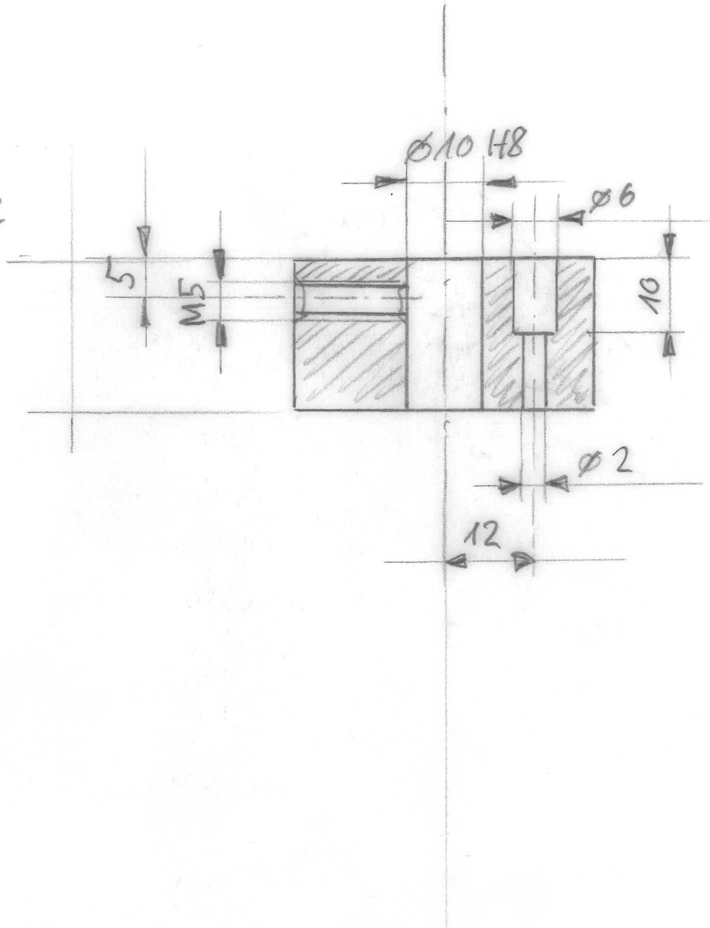

Supplement: S2 Supplementary Material — (PDF) [file pone.0147558.s003.pdf]
