# Supplementary material for: An eDNA Assay to Monitor a Globally Invasive Fish Species from Flowing Freshwater
Source: PLoS One. 2016 Jan 27;11(1):e0147558. doi: 10.1371/journal.pone.0147558 (PMC4729461; doi:10.1371/journal.pone.0147558)
Supplement: S3 Supplementary Material — (DOCX) [file pone.0147558.s004.docx]

**Extraction Protocol Tests**

Supplementary Material

Adrian-Kalchhauser and Burkhardt-Holm, “An eDNA assay to monitor a globally invasive fish species from flowing freshwater.”

**Method 1**

**Ethanol Precipitation with subsequent extraction with Qiagen Blood and Tissue Kit as in Ficetola et al., 2008**

**Sample collection, storage and precipitation**

- 500 ml water sample
- bottle kept on ice until processing in lab
- distribute 15 ml water aliquots in 50 ml Falcon tubes
- Add 33 ml 100% ethanol and 1.5 ml 3M sodium acetate to each 15 ml water sample
- mix, precipitate at -80°C overnight

**DNA Extraction (with Qiagen Blood and Tissue Kit – Prod. Nr. 69504)**

- Centrifuge samples at 5500 g for 35 minutes at 6°C to pellet DNA
- Carefully discard supernatant (DNA pellet may not be visible)
- Re-suspend pellet in 360 µl ATL Buffer (from Kit) and 40 µl Proteinase K (600 mAU/ml)
- Pipet into an 1.5 ml Eppendorf tube
- Lyse on a shaking heating block at 56°C (750 RPM) overnight
- Add 400 µl AL Buffer and vortex for 10 seconds at 2000 RPM
- Add 400 µl 100% ethanol and vortex for 10 seconds at 2000 RPM
- Transfer to DNA spin-column (place in a collection tube)
- Centrifuge at 8000 RPM for 1 minute and discard flow-through
- Add 500 µl AW1 Buffer to pin-column (place in a new collection tube), centrifuge at 8000 RPM for 1 minute and discard flow-through
- Add 500 µl AW2 Buffer to spin-column (in new collection tube), centrifuge at 14000 RPM for 3 minutes and discard flow-through
- Place spin-column in an 1.5 ml Eppendorf tube and pipet 100 µl AE Elution buffer directly onto the membrane. Incubate sample for 3 minutes
- Centrifuge sample at 8000 RPM for 1 minute, measure DNA concentration with Nano-Drop and store DNA at -80°C.

**Method 2**

**Filtration with subsequent extraction with Qiagen Blood and Tissue Kit as in Deiner et al, 2014**

**Sample collection**

- Collect 500 ml water
- Keep bottle on ice until processing in the lab

**Filtration (as in Deiner et al., 2014)**

- Place a 0.7 µm Glass fibre filter (Whatman, Prod-Nr. WHA1825025, 25mm diameter) in a filter housing (Swinnex EMD – Millipore, Prod-Nr. SX0002500)
- Attach filter housing to a disposable 20 ml syringe (VWR, Prod-Nr. 720-2521), remove plunger
- Pour water into the syringe and insert plunger
- Push water through filter by hand (flow rate ≈ 1 ml per 10 seconds) and collect flow-through in a glass beaker
- Refill syringe and repeat
- Remove filter from filter housing with sterile tweezers
- Place filter in a 1.5 ml Eppendorf tube
- Use 3 filters for each 500 ml water sample

**DNA Extraction for both filtration methods (with Qiagen Blood and Tissue Kit – Prod. Nr. 69504)**

- Add 360 µl ATL Buffer and 40 µl Proteinase K (600 mAU/ ml) to each tube
- Lyse on a shaking heating block at 56°C (750 RPM) overnight
- Add 400 µl AL Buffer and vortex for 10 seconds at 2000 RPM
- Add 400 µl 100% ethanol and vortex for 10 seconds at 2000 RPM
- Transfer to DNA spin-column (place in a collection tube)
- Centrifuge at 8000 RPM for 1 minute and discard flow-through
- Add 500 µl AW1 Buffer to pin-column (place in a new collection tube), centrifuge at 8000 RPM for 1 minute and discard flow-through
- Add 500 µl AW2 Buffer to spin-column (in new collection tube), centrifuge at 14000 RPM for 3 minutes and discard flow-through
- Place spin-column in an 1.5 ml Eppendorf tube and pipet 100 µl AE Elution buffer directly onto the membrane. Incubate sample for 3 minutes
- Centrifuge sample at 8000 RPM for 1 minute, measure DNA concentration with Nano-Drop and store DNA at -80°C.

**Method 3**

**CTAB extraction method with ethanol precipitation as in Renshaw et al., 2014**

**Sample collection**

- Collect 500 ml water
- Keep bottle on ice until processing in the lab

**Filtration (as in Deiner et al., 2014)**

- Place a 0.7 µm Glass fibre filter (Whatman, Prod-Nr. WHA1825025, 25mm diameter) in a filter housing (Swinnex EMD – Millipore, Prod-Nr. SX0002500)
- Attach filter housing to a disposable 20 ml syringe (VWR, Prod-Nr. 720-2521), remove plunger
- Pour water into the syringe and insert plunger
- Push water through filter by hand (flow rate ≈ 1 ml per 10 seconds) and collect flow-through in a glass beaker
- Refill syringe and repeat
- Remove filter from filter housing with sterile tweezers
- Place filter in a 1.5 ml Eppendorf tube
- Use 3 filters for each 500 ml water sample

**Lysis**

- Add 450 ml CTAB Lysis Buffer (Axonlab, Prod-Nr. A 4150.0500) to each Eppendorf tube
- Incubate each tube on a heating block (no shaking) at 50°C for 40 minutes and then store Eppendorf tubes at -80°C overnight

**DNA Extraction and Precipitation**

- Heat Eppendorf tubes containing filters at 65°C for 10 minutes on a heating block
- Squeeze filters with sterile tweezers before removing them from tubes (to extract as much DNA as possible from them)
- Add 450 µl of Phenol-Chloroform-Isoamyl alcohol (25:24:1; pH 8.0; Axonlab, Prod-Nr. A 0944.0100) and vortex for 10 seconds at 2000 RPM
- Centrifuge at 10’000 RPM for 5 minutes at 6°C, pipet off supernatant and transfer it to a new 1.5 ml Eppendorf tube
- Add 450 µl of Chloroform-Isoamyl alcohol (24:1; Axonlab, Prod-Nr. A 1935.0100) and vortex for 10 seconds at 2000 RPM
- Centrifuge at 10’000 RPM for 5 minutes at 6°C, pipet off supernatant and transfer it to a new 1.5 ml Eppendorf tube (all steps done at RT)
- Add 40 µl of 5M NaCl and 900 µl of cold 100% ethanol to each tube
- Precipitate at -80° overnight
- Centrifuge samples at 10’000 RPM, 30 minutes, 4°C
- Remove supernatant and add 900 µl 70% ethanol; centrifuge at 10’000 RPM, 30 minutes, 4°C
- Remove supernatant and air dry samples for 15 minutes
- Resuspend DNA in 100 µl AE Buffer (from Qiagen DNeasy Extraction Kit), measure concentration with Nano-Drop and store DNA at -80°C
