# Supplementary material for: An eDNA Assay to Monitor a Globally Invasive Fish Species from Flowing Freshwater
Source: PLoS One. 2016 Jan 27;11(1):e0147558. doi: 10.1371/journal.pone.0147558 (PMC4729461; doi:10.1371/journal.pone.0147558)
Supplement: S4 Supplementary Material — (DOCX) [file pone.0147558.s005.docx]

**Full and detailed protocol**

Supplementary Material

Adrian-Kalchhauser and Burkhardt-Holm, “An eDNA assay to monitor a globally invasive fish species from flowing freshwater.”

**Field sampling**

**Items needed**

- water sampler, tip covered with sterile plastic bag, held in place by Velcro bands
- 500 ml Nalgene wide-mouth bottle (autoclaved; Faust Laborbedarf, Prod-Nr. 9.103.055) (1 bottle per water sample)
- Sterile plastic gloves (1 pair for each site)
- Zip-loc or plastic bag for disposables
- Styrofoam box with ice
- 1 roll of multi-purpose tissue for cleaning
- Distilled water (at least 2 litres)
- 1 litre of sodium hypochlorite (for example, a 1:5 dilution of CL00.1410 from Chem-Lab, 13% active chlorine)
- Water-proof marker
- Permissions from harbor authorities, safety vests

**Procedure**

- Wear gloves!!
- Remove Velcro bands from sampler
- Extend sampler to full length
- Remove plastic bag from mouth-piece
- Open 500 ml Nalgene wide-mouth bottle, attach to sampler, and screw close the sampler cover
- Insert sampler into water and push down until you hit the bottom
- Pull cable to open cover and let Nalgene bottle be completely filled (check for air bubbles)
- Retract sampler
- Carefully remove bottle from sampler, close screw cap and put it back on ice
- Insert sampler mouth-piece into a bottle with sodium hypochlorite, shake, remove. Wrap with tissue and cover with plastic bag. Remove sodium hypochlorite by rinsing with distilled water or in the river at next site.
- Repeat procedure for additional samples. Use a new pair of gloves at each site.

**DNA extraction**

**Items needed**

- Sterile plastic gloves
- Container with ice
- Water samples on ice
- 1.5 ml tubes (3 per 500 ml water sample, pre-labelled)
- Small glass petri dishes (1 for each sample)
- 0.7 µm Glass fibre filters (Whatman, Prod-Nr. WHA1825025, 25 mm diameter) (3 per 500 ml water sample)
- filter housing (Swinnex EMD – Millipore, Prod-Nr. SX0002500) (1 per 500 ml water sample)
- 20 ml syringe (VWR, Prod-Nr. 720-2521) (1 per 500 ml water sample)
- 3 sterile tweezers
- Ethanol 70% to clean surfaces
- 1x 100 ml glass beaker with sodium hypochlorite to decontaminate tweezers
- 1x 100 ml glass beaker with distilled water to rinse tweezers before next use
- 1x 1000 ml glass or plastic beaker to collect filtered water
- Qiagen DNeasy Blood and Tissue Kit Prod-Nr. 69504

**water filtration**

- Wear gloves!
- Clean surfaces with ethanol, cover area with one tissue, place glass petri dish to hold filter housing during operations onto tissue.
- Place filter in filter housing with sterile tweezers and attach cover.
- Open first 500 ml Nalgene bottle. Start with negative control, work your way from the supposedly negative samples to the positive controls.
- Draw 20 ml water into a fresh syringe (or remove plunger and pour 20 ml into syringe).
- Attach syringe to filter housing.
- Press water through filter over a 1000 ml glass beaker.
- Filter ca. 170 ml water per filter, use three filters for every 500 ml sample.
- Remove filter housing cover and carefully fold filter in half using tweezers. Put tweezers in sodium hypochlorite to decontaminate and use a new pair for the next filter.
- Put folded filters in a labelled 1.5 ml tube and keep on ice.
- Repeat procedure for the other three filters. Put each filter into a separate tube.
- Repeat procedure for all samples.
  - Use new tissues and a new glass petri dish for every sample!
  - Wear fresh gloves for every new bottle!
  - Use new tweezers for new bottle!
  - Use new filter housing for every fresh bottle!
  - Use new syringe for every fresh bottle!

**DNA extraction and purification**

- Move samples from filtration area to molecular biology lab.
- Wear gloves!
- Clean surfaces!
- Add 360µl ATL Lysis Buffer and 40 µl Proteinase K to every tube.
- Extract DNA from filters on a shaking heating block at 56°C with 750 RPM overnight.
- After overnight lysis, remove filter from tube with tweezers, squeeze out liquid, and discard filters. Use fresh tweezers for every new water sample.
- Add 400 µl AL Buffer to the sample, vortex for 10 seconds at 2000 RPM.
- Add 400 µl 100% ethanol to tube, vortex for 10 seconds at 2000 RPM.
- Pipet 700 µl of one of the three aliquots per water sample in a Mini-Spin column, centrifuge at 8000 RPM for 60 seconds. Discard flow-through. Re-fill column with the next 700 µl, until all liquid that belongs to the same water sample has gone through a single column.
- Place Mini-Spin column in a new collection tube and add 500 µl AW1 Buffer.
- Centrifuge at 8000 RPM for 60 seconds.
- Discard collection tube and place spin column in a new collection tube.
- Add 500 µl AW2 Buffer.
- Centrifuge at 14000 RPM for 3 minutes.
- Discard collection tube and place spin column in a labelled 1.5 ml tube.
- Pipet 50 µl AE Buffer (or less / more, for increased concentrations or increased amounts) directly onto the membrane and incubate at room temperature for 3 minutes.
- Centrifuge tubes with spin column at 8000 RPM for 60 seconds.
- Discard Spin-Columns and close tubes.
- Unless used immediately, store extracted DNA at -80°C.

**Amplification**

**Items needed**

- Sterile plastic gloves
- Illustra PCR Beads (Fisher Scientific Schweiz; Prod-Nr. 10678095)
- Ultra pure water
- eDNA Primer, 10 µM
- Extracted DNA
- Positive control (5 ng/µl)
- BSA (Bovine Serum Albumin; BioConcept; Prod-Nr. B9000S)
- Container with ice
- TAE buffer
- Agarose
- Gel dye, for example Gel Red
- Loading dye
- DNA ladder, for example Solis Biodyne, 07-11-00050

**eDNA Master Mix**

- Wear gloves!
- Clean surfaces!
- Work under flow-hood!
- Label the tube strips
- Make Mastermix for your number of samples + 10% (20 samples 🡪 22x Mastermix):
- 19.75 µl H2O ultrapure
- 1.25 µl BSA
- 0.5 µl Forward Primer
- 0.5 µl Reverse Primer
- Distribute 22 µl per tube
- Add 3 µl of extracted DNA to total volume of 25 µl. You may want to use less if you suspect inhibition, in that case, adjust water volume accordingly.
- Use 1 µl DNA (5 ng conc.) for positive control 🡪 adjust H2O content to 21.75 µl for positive control

Use ultrapure water as negative control.

- Make sure all tubes are fully closed, flick and spin them.

**Touchdown PCR**

- Place the PCR tubes in a PCR machine.
- Start the following PCR program:

| Temperature | Time | Cycles | Phase |
| --- | --- | --- | --- |
|  |  |  |  |
| 95°C | 5’ | 1 | Denature |
| 95°C | 1’ | 15 | Denature |
| 65°C (-1°C per cycle) | 30’’ |  | Initial Annealing |
| 72°C | 15’’ |  | Extension |
| 95°C | 30’’ | 35 | Denature |
| 50°C | 30’’ |  | Final Annealing |
| 72°C | 15’’ |  | Extension |
| 72°C | 7’ | 1 | Final Extension |
| 4°C | ∞ |  | Cooling |

**Agarose Gel Electrophoresis**

- Prepare a 2.5% agarose Gel using 100 ml 1x TAE and 2.5 g Agarose
- Melt in microwave, add 4 µl Gel-Red, mix, pour
- Let agarose solidify for at least 30 minutes
- Add loading dye to the samples
- Load 1 µl 100 bp ladder (Solis Biodyne, 07-11-00050)
- Load the same amount of PCR in all wells. Avoid spillover, spillover creates false positives. You may want to leave one well empty between loaded lanes.
- Run electrophoresis for 35 minutes.
